# Characterization of the melanopsin gene (*Opn4x*) of diurnal and nocturnal snakes

**DOI:** 10.1186/s12862-019-1500-6

**Published:** 2019-08-28

**Authors:** Einat Hauzman, Venkatasushma Kalava, Daniela Maria Oliveira Bonci, Dora Fix Ventura

**Affiliations:** 1 Departamento de Psicologia Experimental, Instituto de Psicologia, Universidade de São Paulo, Av. Professor Mello Moraes, 1721, Bloco A - Sala D9. Butantã, São Paulo, SP 05508-030 Brazil; 2 Instituto Israelita de Ensino e Pesquisa Albert Einstein, São Paulo, Brazil; 3 Christian Brothers University, Memphis, TN USA

**Keywords:** Melanopsin, Photopigment, Snakes, Retina, Circadian rhythm, dN/dS

## Abstract

**Background:**

A number of non-visual responses to light in vertebrates, such as circadian rhythm control and pupillary light reflex, are mediated by melanopsins, G-protein coupled membrane receptors, conjugated to a retinal chromophore. In non-mammalian vertebrates, melanopsin expression is variable within the retina and extra-ocular tissues. Two paralog melanopsin genes were classified in vertebrates, *Opn4x* and *Opn4m*. Snakes are highly diversified vertebrates with a wide range of daily activity patterns, which raises questions about differences in structure, function and expression pattern of their melanopsin genes. In this study, we analyzed the melanopsin genes expressed in the retinas of 18 snake species from three families (Viperidae, Elapidae, and Colubridae), and also investigated extra-retinal tissue expression.

**Results:**

Phylogenetic analysis revealed that the amplified gene belongs to the *Opn4x* group, and no expression of the *Opn4m* was found. The same paralog is expressed in the iris, but no extra-ocular expression was detected. Molecular evolutionary analysis indicated that melanopsins are evolving primarily under strong purifying selection, although lower evolutionary constraint was detected in snake lineages (ω = 0.2), compared to non-snake *Opn4x* and *Opn4m* (ω = 0.1). Statistical analysis of selective constraint suggests that snake phylogenetic relationships have driven stronger effects on melanopsin evolution, than the species activity pattern. In situ hybridization revealed the presence of melanopsin within cells in the outer and inner nuclear layers, in the ganglion cell layer, and intense labeling in the optic nerve.

**Conclusions:**

The loss of the *Opn4m* gene and extra-ocular photosensitive tissues in snakes may be associated with a prolonged nocturnal/mesopic bottleneck in the early history of snake evolution. The presence of melanopsin-containing cells in all retinal nuclear layers indicates a globally photosensitive retina, and the expression in classic photoreceptor cells suggest a regionalized co-expression of melanopsin and visual opsins.

**Electronic supplementary material:**

The online version of this article (10.1186/s12862-019-1500-6) contains supplementary material, which is available to authorized users.

## Background

For their survival, animals depend on the ability to capture and process information from the surrounding environment. Information acquired through light processing channels is captured by photoreceptor cells, whose photopigments absorb photons and lead to the generation of neural responses to light. Responses to light by photoreceptor cells may trigger circuits dedicated to visual processing and image formation or may activate neural circuits with non-visual functions, such as circadian rhythm control, pupil light reflex, and skin color changes [1]. The visual process is initiated by visual photopigments, opsins and rhodopsins, coupled to a retinal chromophore, located in rods and cones. Non-visual responses to light rely on the activity of a number of opsin classes, for instance, encephalopsins, parapinopsins, and melanopsins [2–6]. Non-visual opsins may be located in the retina and iris, and in extra-ocular photosensitive tissues, such as skin, pineal gland, brain, and liver, depending on the opsin group and vertebrate class [7].

Opsins are membrane receptors that belong to the G-protein-coupled receptor family, with seven transmembrane domains, characterized by their ability to bind a vitamin A-based chromophore, usually the 11-cis-retinal, via a Schiff base linkage in a conserved Lysine residue, in the seventh transmembrane α-helix. Absorption of a photon by the chromophore causes its photoisomerization from 11-cis to all-trans form, which leads to conformational changes of the opsin, allowing a G-protein to bind and activate the phototransduction cascade within the photoreceptor cell [8–10]. This process leads to the closure or opening of ion channels in the plasma membrane, depending on the photoreceptor cell type, and the photoreceptor’s electrical response to light [11, 12].

In the 1990s, the discovery of a new photopigment expressed in dermal melanophores of the frog *Xenopus laevis* [5] boosted new investigations for the understanding of the functions and the possible relationship of these photopigments with non-visual responses to light. This newly discovered photopigment, named melanopsin, was then identified in the inner retinas of mammals [6]. Studies with knockout mice models revealed the role of this photopigment in the circadian rhythm control [13–16] and following studies showed that melanopsins are responsible for other non-visual responses to light, such as pupillary light reflex, melatonin suppression, and skin color changes [17–27].

Comparative studies on the melanopsin gene have shown the presence of two paralogs within vertebrate genomes, the *Xenopus*-like (*Opn4x*) and the mammalian-like (*Opn4m*) [28], which were originated from a duplication event in the early history of vertebrates evolution [29]. In mammals, only the *Opn4m* gene is expressed in a subset of intrinsically photosensitive retinal ganglion cells (ipRGCs) [30]. Non-mammalian vertebrates may express both *Opn4m* and *Opn4x* genes, with varied tissue expression patterns [5, 28, 31–38]. The loss of the *Opn4x* paralog in the mammalian genome and the loss of extra-ocular photosensitive tissues was associated with a “nocturnal bottleneck” in the early history of mammalian evolution [39]. On the other hand, in teleost fish, later duplication events led to new melanopsin paralogs expressed in different classes of retinal neurons, and with distinct functional properties [40, 41].

The role of the Opn4m photopigment in mediating photoentrainment, circadian rhythm regulation, melatonin suppression and pupillary light reflex, has been demonstrated in mammals [14, 16–18, 20, 23, 42, 43]. However, the specific functions of each melanopsin paralog in non-mammalian vertebrates still need extensive investigation in the context of its complex expression pattern. For instance, in chicken, variations of *Opn4x* and *Opn4m* expression levels and location within the retina were observed during different stages of development and throughout the day [44]. Whereas *Opn4m* expression is restricted to a subpopulation of ganglion cells during all life stages, *Opn4x* expression is limited to ipRGCs and the optic nerve only at early embryonic stages, and later it is expressed in horizontal cells, with daily rhythmic expression levels in the mature retina [34, 35, 44, 45].

In reptiles, melanopsin expression has been characterized in a few species, including the freshwater red ear turtle *Trachemys scripta elegans*, which expresses the *Opn4m* in the retina [46], the ruin lizard *Podarcis sicula*, which expresses the *Opn4x* in the retina, lateral eye and brain [47], and in sea snakes, which express the *Opn4x* in the skin [48]. In the Elapidae sea-snakes *Aipysurus laevis* and *A. tenuis*, the expression of melanopsin in the skin was associated to dermal phototaxis of the tail, a behavior observed in this group of marine snakes [48]. Genome data [49–51] and eye transcriptome analysis [52], have indicated that snakes have lost the *Opn4m* gene prior to the origin of Serpentes [50] and had only kept the *Opn4x* paralog.

The suborder Serpentes comprises a highly diversified group of vertebrates and represents a valuable model to investigate specific functions and expression patterns of photosensitive proteins, which may contribute to the understanding of the evolution of vertebrate circadian timing regulation. The diversity of the group, with more than 3700 living species [53] exhibits a number of adaptations to their ecological niches, which include distinct daily activity patterns [54–57]. These adaptations raise intriguing questions about selective pressure acting on specific genes related to circadian activity control. In this study, we isolated the melanopsin gene of diurnal and nocturnal snakes from different families, determined its phylogenetic position, and characterized its tissue-specific expression pattern, and retinal location. We also investigated selection patterns of molecular evolution in a phylogenetic framework, using several models to detect selection signals in the protein level. Our results bring new light and increase the knowledge on the evolution of melanopsins and photosensory systems in reptiles.

## Results

### Gene identification and phylogenetic position of melanopsin of snakes

Partial sequences of the melanopsin gene (~ 1400 bp) of 18 snake species (Additional file 1: Table S1) were successfully amplified with the primers designed based on snake melanopsin sequences. No amplification was observed in PCRs performed with primers designed based on the *Opn4m*. All sequences have been deposited at GenBank (accession numbers: MN241125- MN241142) (Additional file 1: Table S1).

The amplified fragments possessed the general features of melanopsins and the G-protein-coupled receptor proteins. Alignment of the amino acid sequences reveals some conserved key features, including a Lysine residue (284 K), located in the seventh transmembrane domain, that acts as the retinal attachment site through a Schiff base [9], and a Tyrosine located in the equivalent position of the Glutamate Schiff base counterion of visual opsins (Y92) (Additional file 2). Other conserved features include a Glutamate (E) at residue 161, which serve as possible counterion site [58, 59], two Cysteine (C) residues at sites 89 and 167 for disulfide bond formation, and the D113/R114/Y115 motif in the third transmembrane for G protein binding [60] (Additional file 2).

**Fig. 1:**
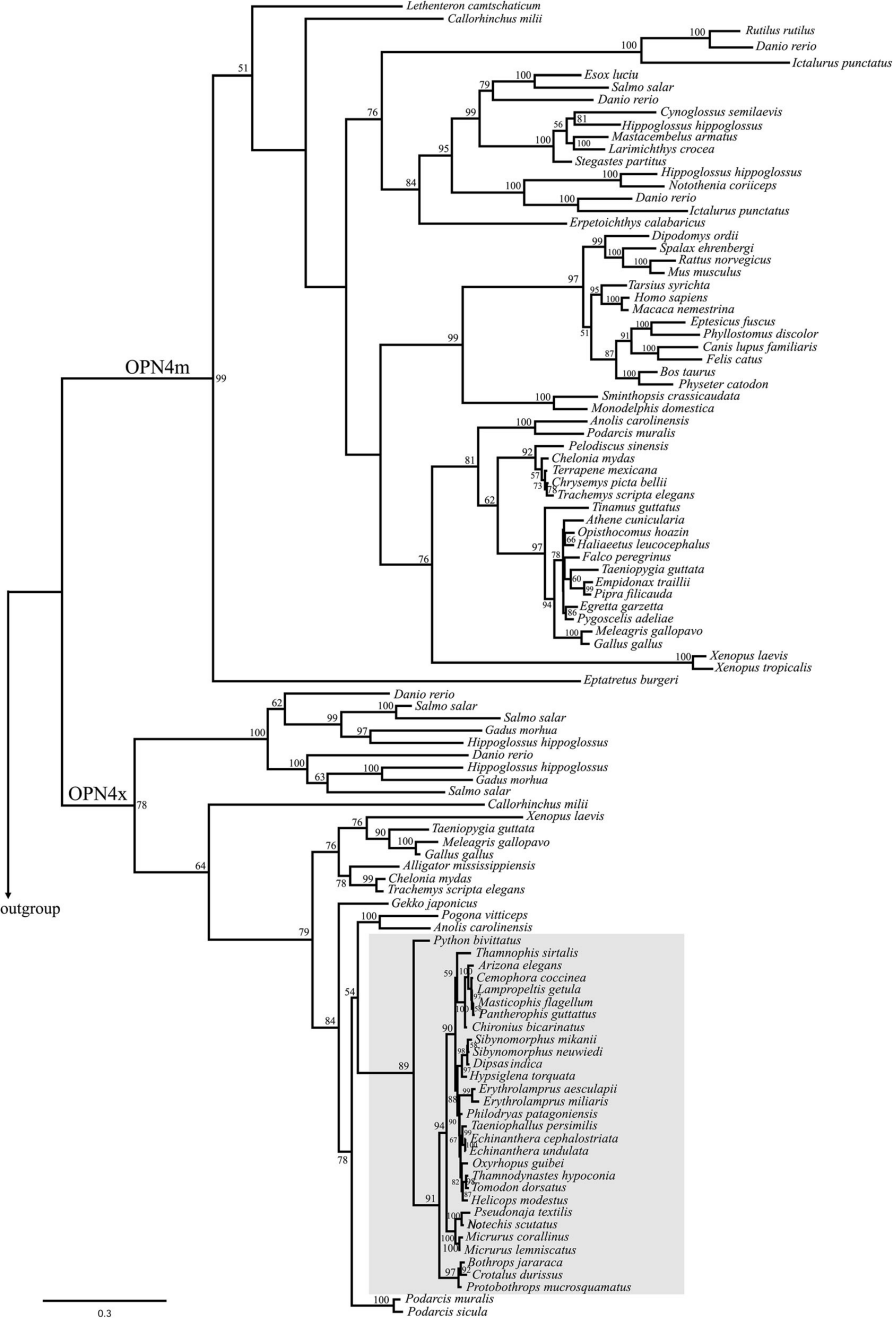
Phylogenetic reconstruction of vertebrate melanopsin genes *Opn4m* and *Opn4x*, by Maximum Likelihood (ML). Snake melanopsin sequences (gray box) correspond to the *Opn4x* gene. The amphioxus melanopsin sequence (GenBank: Q4R1I4) was used as outgroup (not shown). ML bootstrap supports are indicated for each resolved node. The scale bar represents the number of nucleotide substitutions per site

The final alignment used for phylogenetic reconstruction was 2397 base pairs long. ML reconstruction recovered the monophyly of the two melanopsin genes, *Opn4m* and *Opn4x* (bootstrap supports: 99 and 78, respectively), and grouped the melanopsins of snakes within the *Opn4x* group, with high bootstrap support (89) (Fig. 1). The melanopsin tree was highly consistent with snake phylogenies [61] and recovered the monophyly of the three caenophidian families sampled, Viperidae, Elapidae, and Colubridae. In most cases, the topology of internal branches were highly consistent with species phylogeny, with only exception for internal branches of the Colubrinae subfamily [61] (Fig. 1).

### Statistical analysis of molecular evolution

**Fig. 2:**
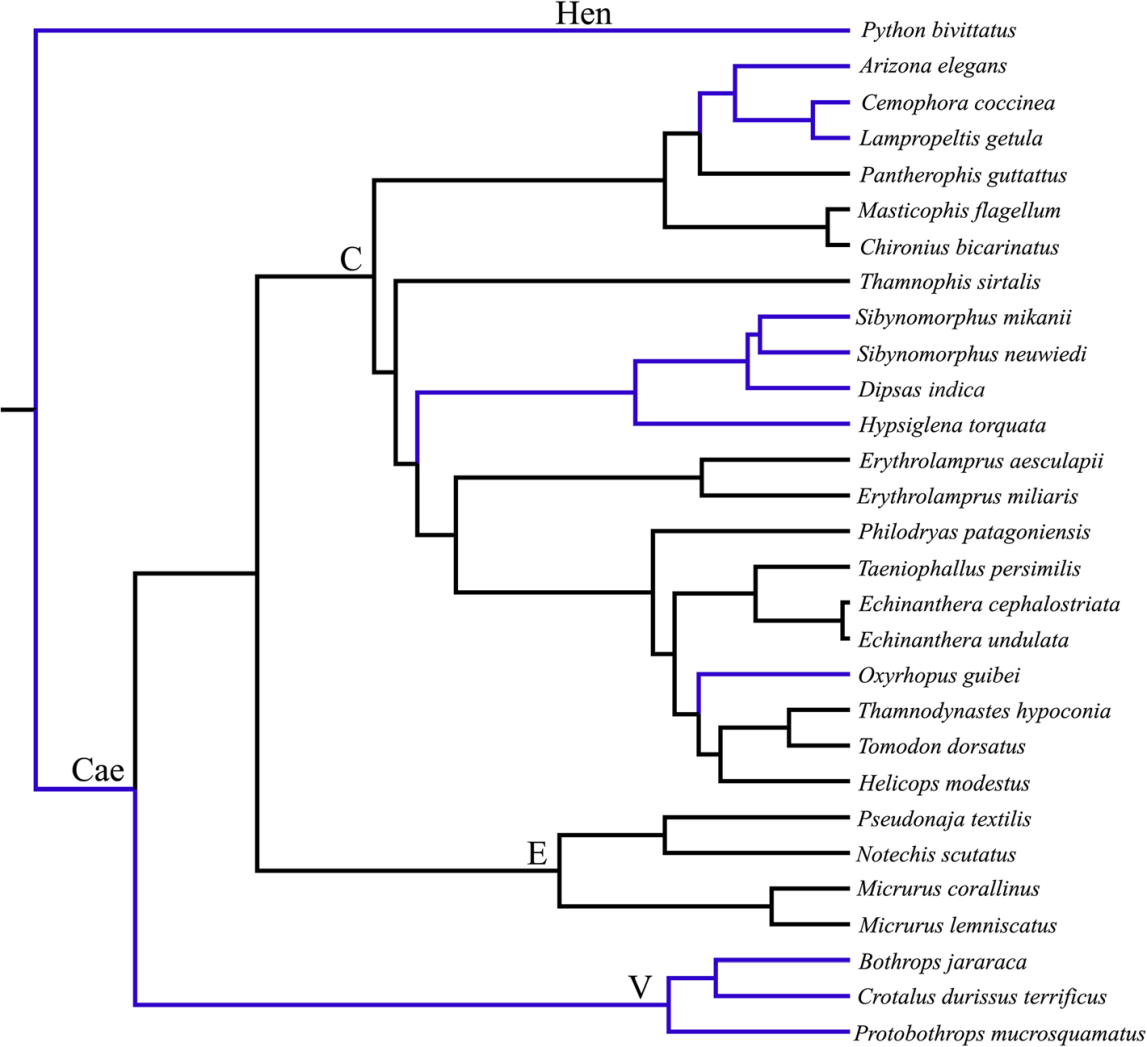
Tree topology used in codon-based likelihood models (random-site, branch-site and CmC models). Nocturnal species are represented by blue lines [56, 57, 62]. Hen, Henophidia; Cae, Caenophidia, V, Viperidae, E, Elapidae, C, colubridae

We investigated the patterns of selection in snake melanopsins using codon-based likelihood models to estimate d_N_/d_S_. For branch model analysis, we used a broad data set with 107 sequences including the *Opn4m* and *Opn4x* genes from different vertebrates, to estimate the overall selective pattern of melanopsins (Additional file 1: Table S2; Sequences alignment: Additional file 3). For random site, branch-site and clade models, we used a smaller data set containing 29 snake melanopsin coding sequences, 18 of which generated in this study (Fig. 2; Sequences alignment: Additional file 4).

Statistical analysis of branch models showed an overall low ω rate under the null model (ω = 0.107), which indicates a strong evolutionary constraint on the melanopsin genes (Table 1). Likelihood ratio tests (LRTs) showed that the two-partition model, with independent ω values for *Opn4m* (ω = 0.097) and *Opn4x* (ω = 0.125), and the three-partition model, with independent ω values for *Opn4m*, non-snakes *Opn4x* (ω = 0.114), and snakes *Opn4x* (ω = 0.204), were significantly better than the null (1ω) model (Table 1).

**Table 1:** Parameter estimates and log likelihood values under branch models for the melanopsin genes *Opn4m* and *Opn4x*

Branch Model	np	ln L	κ	Parameters	BIC		LRT	df	P
1ω	213	−28,548.3	2.0	ω = 0.107	57,624.8	–			
2ω	214	−28,539.9	2.0	OPN4m: ω = 0.097; OPN4x: ω = 0.125	57,601.5	1ω	17	1	0.000
3ω	215	−28,529.4	2.0	OPN4m: ω = 0.097; OPN4x: ω = 0.11; Snakes-OPN4x: ω = 0.20	57,591.9	2ω	21	1	0.000
						1ω	38	2	0.000

*np* number of parameters, *ln L* ln likelihood, *κ* transition/transversion ratio, *BIC* Bayesian information criterion, *LRT* likelihood ratio test, *df* degrees of freedom

The LRT comparison of random-site models showed significant difference between M0 and M3 and between M7/M8 and M8a/M8 models (Table 2), indicating that relative rates of substitution are variable among sites. Models M2a, M3 and M8 had a significantly better fit than the alternative models (*p* < 0.05), and several sites were indicated by BEB as under positive selection (Additional file 1: Table S3).

**Table 2:** Likelihood ratio tests for Random-Sites Models of Snake *Opn4x* Species Tree

Model	np	ln L	κ	Parameters		LRT	df	p
M0	57	− 6516.8	3.4	ω_0_ = 0.40				
M1a	58	− 6417.8	3.4	ω_0_ = 0.08 (p_0_ = 0.68); ω_1_ = 1.0 (0.32)				
M2a	60	− 6411.7	3.5	ω_0_ = 0.1 (p_0_ = 0.69); ω_1_ = 1.0 (p_1_ = 0.26); ω_2_ = 2.45 (p_2_ = 0.05)	M1a	12.2	2	0.002
M3	61	− 6409.6	3.5	ω_0_ = 0.0 (p_0_ = 0.4); ω_1_ = 0.43 (p_1_ = 0.48); ω_2_ = 1.93 (p_2_ = 0.12)	M0	214.4	4	0.000
M7	58	− 6421.1	3.4	*p* = 0.17; q = 0.28				
M8a	59	−6417.7	3.4	p_0_ = 0.69, *p* = 1.57, q = 14.77 (p_1_ = 0.31, ω = 1.0)				
M8	60	− 6410.1	3.5	p_0_ = 0.91, *p* = 0.36, q = 0.96 (p_1_ = 0.09, ω = 2.09)	M7	22.1	2	0.000
					M8a	15.2	1	0.000

*np* number of parameters, *ln L* ln likelihood, *κ* transition/transversion ratio, *LRT* likelihood ratio test, *df* degrees of freedom

We used clade models to investigate the effects of species phylogeny and daily activity pattern on the evolution of snake melanopsins. Different CmC partition models were implemented to test whether diurnal or nocturnal activities may have driven divergences in melanopsin evolution. LRTs showed that all implemented partitions considering both phylogeny and daily activity pattern were better than the null model, M2a_rel (Table 3). Two different partition models were tested to investigate the effects of daily activity pattern, a two-partition which isolates diurnal snakes from all nocturnal lineages, and a three-partition which separated the henophidian snake *Python bivitattus* in one clade, and diurnal and nocturnal caenophidian species in independent foreground clades (Fig. 2). LRT comparisons indicated that the three-partition model, was significantly better than the two-partition model, and diurnal lineages had lower d_N_/d_S_ value (ω = 0.06) compared to nocturnal (ω = 0.2) (Table 3). According to LRT and BIC comparisons, the best fitting model overall was the phylogenetic four-partition model which isolates each snake family in a separate foreground branch, with lower ω value for Colubridae snakes (ω = 0.03) (Table 3). The results from CmC models suggest that although daily activity pattern seems to apply different constrains on the evolution of melanopsin gene in snakes, with stronger signals of purifying selection in diurnal lineages, snakes phylogenetic relationships have driven stronger effects on *Opn4x* evolution.

**Table 3:** Clade Model Tests (CmC) for divergence partitioned by snakes phylogeny and by daily activity pattern

Model	np	ln L	κ	Parameters	BIC		LRT	df	P
M2_rel	60	−6417.6	3.38	ω_0_ = 0.00 (p_0_ = 0.24); ω_1_ = 1.0 (p_1_ = 0.30); ω_2_ = 0.15 (p_2_ = 0.46)	12,996.9	–			
CmC: 4 part (phylo)	63	− 6399.2	3.36	ω_0_ = 0.00 (p_0_ = 0.26); ω_1_ = 1.0 (p_1_ = 0.31); ω_2_ = 0.19 (p_2_ = 0.45); Hen: ω = 0.58; Vip: ω = 0.34; Elap: ω = 0.19; Col: ω = 0.03 (*p* = 0.44)	12,968.2	M2_rel	36.8	3	0.000
						3 part (phylo)	5.36	1	0.021
						3 part (D x N)	11.7	1	0.001
						2 part (phylo)	16.9	2	0.000
CmC: 3 part (phylo)	62	− 6401.9	3.38	ω_0_ = 0.03 (p_0_ = 0.4); ω_1_ = 1.0 (p_1_ = 0.3); Hen: ω = 0.84; Vip: ω = 0.5; Col+Elap: ω = 0.03 (p = 0.3)	12,970.9	M2_rel	31.5	2	0.000
						2 part (phylo)	11.5	1	0.001
						2 part (D x N)	14.3	1	0.000
CmC: 2 part (phylo)	61	− 6407.6	1.75	ω_0_ = 0.00 (p_0_ = 0.29); ω_1_ = 1.0 (p_1_ = 0.30); Hen: ω = 0.63; Caen: ω = 0.11 (*p* = 0.46)	12,979.7	M2_rel	19.9	1	0.000
CmC 3 part (D x N)	62	− 6404.8	3.37	ω_0_ = 0.00 (p_0_ = 0.26); ω_1_ = 1.0 (p_1_ = 0.29); Hen: ω_2_ = 0.56; N: ω = 0.2; D: ω = 0.06 (*p* = 0.45)	12,977.3	M2_rel	25.1	2	0.000
						2 part (phylo)	5.7	1	0.017
						2 part (D x N)	7.7	1	0.004
CmC 2 part (D x N)	61	− 6408.9	3.38	ω_0_ = 0.00 (p_0_ = 0.19); ω_1_ = 1.0 (p_1_ = 0.3); N: ω_2_ = 0.24; D: ω = 0.05 (*p* = 0.51)	12,982.2	M2_rel	17.4	1	0.000

*np* number of parameters, *ln L* ln likelihood, *κ* transition/transversion ratio, *BIC* Bayesian information criterion, *LRT* likelihood ratio test, *df* degrees of freedom, *phylo* phylogenetic partition, *D* diurnal, *N* nocturnal, *Hen* Henophidia, *Caen* Caenophidia, *Vip* Viperidae, *Elap* Elapidae, *Col* Colubridae

To investigate for positive selective sites in specific clades, we used branch-site models, isolating independently diurnal and nocturnal caenophidian lineages in foreground clades, and the three families within the Caenophidia group, Viperidae, Elapidae, and Colubridae. Based on the results from CmC models, we also performed branch-site models isolating diurnal and nocturnal snakes from the Colubridae family, to investigate specific divergent selection in this particular group. The LRT and BIC comparisons indicated that the model designating primarily diurnal snake lineages as foreground branch was slightly better than the null model (*p* = 0.048; Additional file 1: Table S4), and BEB analysis indicated only two sites under positive selection (ω_2_ = 3.3, p_2_ + p_3_ = 0.02; Additional file 1: Table S4). In addition, branch-site model considering the Colubridae family as foreground branch was better than the null model (*p* < 0.05; ω_2_ = 3.7, p_2_ + p_3_ = 0.05; Additional file 1: Table S4), such as the model designating diurnal Colubridae snakes as foreground branch (*p* = 0.003; ω_2_ = 4.2, p_2_ + p_3_ = 0.03; Additional file 1: Table S4).

### Expression pattern and melanopsins location within the retinas

**Fig. 3:**
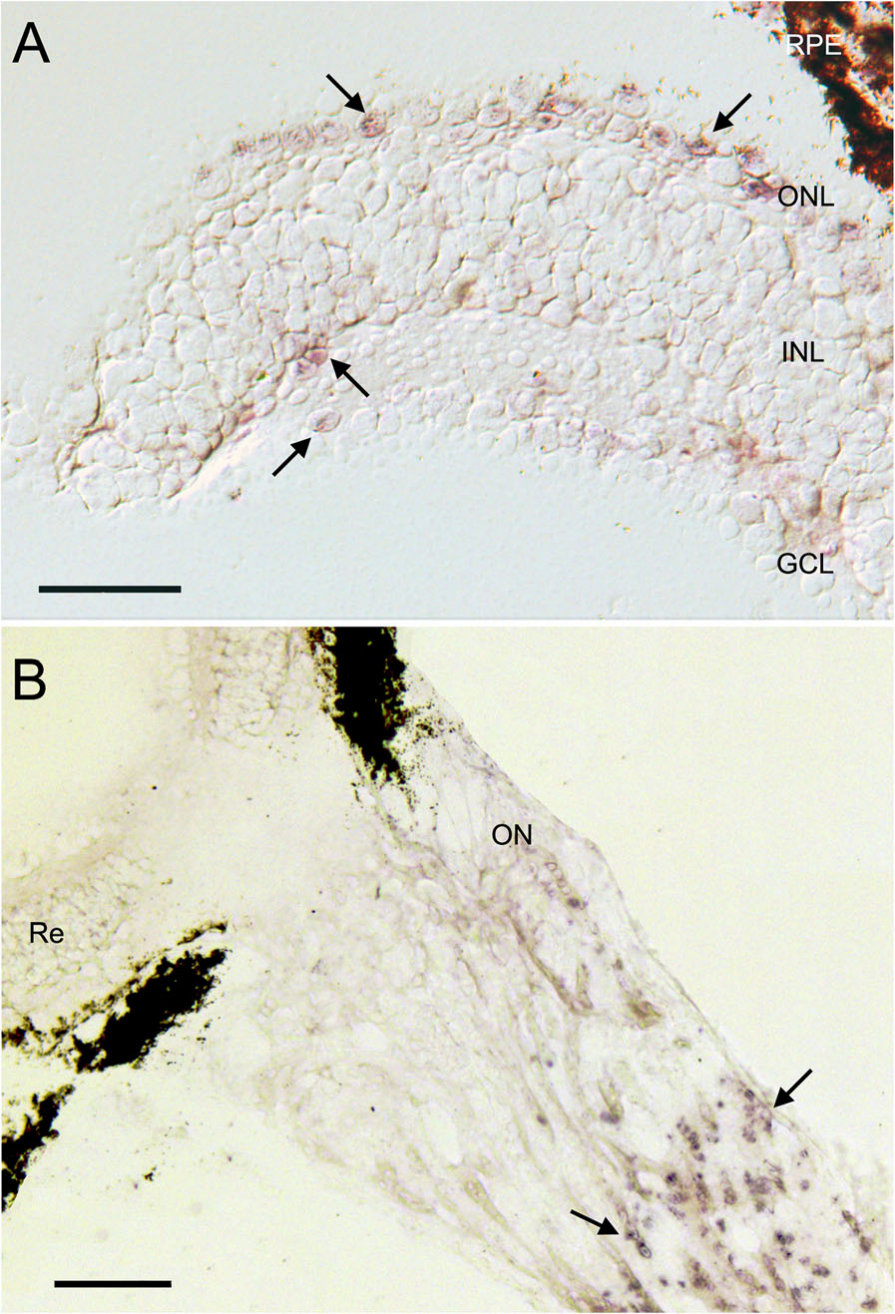
In situ hybridization using ‘antisense’ ‘DIG’ labeled cRNA probes in retinal sections of the diurnal colubrid *Tomodon dorsatus*. **a** Expression of melanopsin mRNA in the outer nuclear layer (ONL), in the inner nuclear layer (INL), and ganglion cell layer (GCL) (arrows). **b** Intense melanopsin expression in the optic nerve (ON) (arrows). No labeling was observed in the retinal sections incubated with ‘sense’ probes (not shown). RPE, retinal pigment epithelium; Re, retina. Scale bars: A = 50 μm, B = 100 μm

Analysis of extra-retinal melanopsin expression revealed the expression of the same melanopsin gene in the iris of three colubrid species, *T. dorsatus*, *S. neuwiedi*, and *O. guibei*. No expression was detected in extra-ocular tissues. In situ hybridization revealed melanopsin expression in the retinal outer nuclear layer, i.e., photoreceptors nuclei, in the inner nuclear layer, in contact with the inner plexiform layer, and in the ganglion cell layer, indicating the presence of ipRGCs (Fig. 3a). Labeling was observed in the retinal edge, but not in retinal core regions. Intense labeling was observed in the optic nerve (Fig. 3b).

## Discussion

This study provides new evolutionary information on the melanopsin gene family and specifically characterizes the *Opn4x* gene and its expression pattern in the retinas of snakes from three different families. The highly diverse class of reptiles is still misrepresented in studies on visual and non-visual photopigments; our analysis provides new information on the characteristics and variability of photoreceptor genes in this group.

The phylogenetic analysis showed that the melanopsin gene of snakes belongs to the *Opn4x* group. The tree topology was highly consistent with snake phylogeny [61]. The branch of snakes formed a monophyletic clade with those of other squamate reptiles. The molecular evolutionary analysis indicates that the melanopsin gene is under evolutionary constraint in snake lineages. Borges et al. [29] found that amino acid substitutions in melanopsins of vertebrates are mainly under negative selection (ω = 0.17), which indicates the important physiological role of this protein for the organism fitness [29]. However, in our analysis, we found evidence for lower functional constraint in snakes, with higher d_N_/d_S_ ratio (ω = 0.204), compared to non-snakes *Opn4x* (ω = 0.114) and *Opn4m* (ω = 0.097) (Table 1). This more relaxed evolutionary pressure in snake *Opn4x* is intriguing. The melanopsin paralog *Opn4m* seems to be absent in the snake genome [49–51], and one would expect that the presence of only one melanopsin copy would imply stronger constraints in the evolution of the remaining gene, to preserve the structure and function of this protein, necessary for a number of physiological functions driven by responses to light.

Previous studies investigated the expression of visual opsin genes in snakes [62–67]. Three classes of visual opsin genes are expressed in retinas of most snakes studied so far: the rhodopsin gene *Rh1*, the short wavelength sensitive opsin gene *Sws1*, and the long wavelength sensitive opsin gene *Lws* [63]. The *Rh2* and *Sws2* genes, sensitive to middle and short wavelengths, are not expressed in snake retinas, and it was suggested that they might have been lost in the ancestor of extant snakes [49, 50, 62–64]. This loss was attributed to nocturnal conditions of ancestral lineages [50–52, 63, 64], as suggested for mammals, which passed through a nocturnal/mesopic bottleneck [39]. In crocodilians, the loss of two cone opsin genes, *Sws1* and *Rh2*, and inactivating mutations found in *Opn4m*, were also associated with long periods of adaptation to dim-light conditions in ancestral lineages [68].

Snakes also seem to have lost other non-visual opsin genes [49–52], and extra-ocular photosensitive tissues, as observed in our analysis, which we may also associate to nocturnal conditions of ancestral lineages [50, 52]. A nocturnal/mesopic bottleneck in the early history of snake evolution may explain the absence of extra-ocular melanopsin expression, analogous to what has been suggested for mammals [29, 69]. However, in the Elapidae sea snakes *Aipysurus laevis* and *A. tenuis*, two non-visual photopigments were identified from skin transcriptome analysis, the melanopsin gene *Opn4x* and the neuropsin gene *Opn5* [48]. The expression of these opsins may be associated with dermal phototaxis in this group of snakes [48], a behavior commonly observed in elongated aquatic vertebrates [70–72]. In our analysis no expression of melanopsin genes were detected in the skin or other extra-ocular tissues.

Our statistical analysis of molecular evolution indicated that species relationships have driven stronger evolutionary constraint on the evolution of melanopsins in snakes, than the species circadian rhythm. Previous studies identified relevant evolutionary features on the visual opsins of snakes, related to the species circadian activity pattern, and different evolutionary constraints were verified among diurnal and nocturnal species [62, 65–67]. Diurnal colubrids have pure cone retinas, with no typical rod-like photoreceptor [39, 73–77], but express the rhodopsin RH1 photopigment, usually expressed in rods [62, 65–67]. Some studies showed evidence that a group of small single cones of diurnal colubrids expresses the RH1 photopigment [65–67], with a considerable blue shift in the absorption peak. Those adaptations reveal the relevance of the circadian activity in the structure, function, and evolution of photopigments of snakes.

Clade models and branch-site models indicated that diurnal activity may have influenced the magnitude of selection acting on the melanopsin gene of snakes, and random site model pointed a number of sites under positive selection (Additional file 1: Tables S3, S4). Among those, residue 307, located in the C-terminal region, close to the seventh transmembrane domain, seems to diverge among diurnal and nocturnal snakes. In all diurnal species, this residue is occupied by an Arginine (R307), while a Lysine (K307) is found in most nocturnal snakes, including nocturnal colubrids from the Dipsadinae subfamily, and the viperids *Bothrops jararaca* and *Protobothrops mucrosquamatus* (Additional file 2). The viperid *Crotalus durissus* had a Threonine (T307), and primarily nocturnal colubrids from the subfamily Colubrinae (*Lampropeltis getula*, *Arizona elegans* and *Cemophora coccinea*) had R307, as found in diurnal species. Among the elapids, all considered diurnal species, only the fossorial coral snake *Micrurus corallinus* had K307, while the aquatic *M. lemniscatus* and the terrestrials *Notechis scutatus* and *Pseudonaja textilis* had R307. Both coral snakes, *M. corallinus* and *M. lemniscatus*, have diurnal activity, but diverge in the daily activity pattern observed in captivity, with a so-called unimodal pattern displayed by *M. lemniscatus*, with peak activity in the morning, and a bimodal pattern in *M. corallinus*, with peak activities in the morning and afternoon (Banci et al., manuscript in preparation). Those differences in activity pattern and habitat use may imply fine adjustments of the circadian entrainment, with variations in the expression levels and functions of the proteins associated with illuminance perception. Based on those observations, we suggest that residue 307 might be an interesting site for investigating specific mechanisms of melanopsin signaling and deactivation, in an attempt to search for divergences, which can ultimately drive differential activity patterns in snakes.

The melanopsin expression in all retinal nuclear layers of *T. dorsatus*, the outer and inner nuclear layers, and the ganglion cell layer, is curious and raises questions on specific functions and signaling pathways and projections of these photosensitive cells. We observed a large number of cones expressing melanopsin only in the retinal edge, close to the *ora serrata*, which suggests a regionalized co-expression of melanopsin and visual opsins. To our knowledge, this is the first study to report melanopsin expression in a cone population in non-teleost vertebrates. In teleost fishes, melanopsins are expressed in all retinal layers, including the photoreceptor layer [40, 41]. Davies et al. [40] suggested that the expression of the *OPN4m-2* in cones might be related to light-dependent adaptations that extend the working range of these photoreceptors, by mediating the sustention of cytoplasmic calcium levels under bright light. Those studies described the expression of five Opn4 isoforms across all retinal neuron types of larvae and adult zebrafish [40, 41], indicating that a globally photosensitive retina would provide adaptation to a dynamic environmental light. In birds, both melanopsin paralogs *Opn4m* and *Opn4x* are expressed in different retinal cells with varied expression along the development [44, 45], which indicates distinct specific functions of the two melanopsins. In chicken retina, *Opn4m* is expressed only in a subpopulation of ganglion cells during all development stages, whereas *Opn4x* is expressed in ganglion cells in early development stages, and later it is rhythmically expressed in horizontal cells [34, 35, 44], indicating highly complex mechanisms for light detection, processing, and transmission to the brain [44]. In snakes, the melanopsin expression pattern, and distribution within the retina of diurnal and nocturnal species still has to be further investigated in order to understand the mechanisms underlying the control of daily activity pattern among the different species.

In conclusion, our analyses provide new insights into the evolution of the melanopsin genes in reptiles. Our results indicate that snakes have lost the *Opn4m* paralog and some species have lost extra-ocular photosensitive tissues, and we attribute this to a prolonged nocturnal/mesopic bottleneck in the early history of snake evolution. We also observed that the melanopsin gene is evolving under strong evolutionary constraint in snakes, and that daily activity pattern does not seem to have strong influence on the molecular evolution of melanopsin gene in snakes. Other features of the melanopsin function may be associated with the diversity of behavior regarding the daily activity patterns of the different species.

## Methods

### Sample information

Single specimens of 18 snake species from the Colubridae (*n* = 14), Elapidae (*n* = 2), and Viperidae (n = 2) families were provided by the Butantan Institute (Additional file 1: Table S1). Snakes were euthanized with a lethal injection of 30 mg/kg of sodium thiopental (Thionembutal). Individuals were kept under natural light conditions and were collected during the morning time. Animal procedures were in accordance with ethical principles of animal management and experimentation of the Brazilian Animal Experiment College (COBEA), and were approved by the Ethics Committee of Animal Research of the Butantan Institute, São Paulo, Brazil (777/10) and the Psychology Institute, University of São Paulo, Brazil (9,635,070,717; 1,805,090,417).

### RNA extraction, polymerase chain reaction (PCR), sequence alignment and gene tree reconstruction

Eyes were enucleated and preserved in RNAlater® (Life Technologies, Carlsbad, CA, USA), at 4 °C. To investigate extra-retinal expression samples of iris, brain, liver, skeletal muscle and skin were collected from three colubrid species, *Tomodon dorsatus*, *Sibynomorphus mikanii* and *Oxyrhopus guibei*. Total RNA extraction and complementary DNA synthesis were performed as described previously [67].

Primers were designed using Primer 3 (v.0.4.0) [78], based on the predicted melanopsin sequences of the snakes *Python bivittatus* and *Ophiophagus hanna* (GenBank: XM_007429400.2 and AZIM01000714.1). Additionally, more specific primers were designed to amplify the melanopsin sequences of the species sampled in this study, based on the initial sequencing results (Additional file 1: Table S5). A pair of primers was also designed based on the *Opn4m* sequence of the red ear turtle *Trachemys scripta elegans* (GenBank: HM197714.1).

The procedures for polymerase chain reactions (PCRs), PCR products purification and DNA sequencing were performed as described previously [67]. Resulting sequences were visualized and aligned with BioEdit v7.0.9 [79] and Geneious v.9.1.3 (GeneMatters Corp.), using the iterative method of global pairwise alignment (MUSCLE and ClustalW) [80, 81]. The alignment included the 18 sequences generated in this study and 88 melanopsin sequences from other vertebrates obtained from GenBank (Additional file 1: Table S2). The melanopsin sequence of the lancelet (*Branchiostoma belcheri*) was used as outgroup (GenBank number: AB205400). Maximum Likelihood (ML) reconstruction was performed on the codon-match nucleotide alignment, using Garli v2.0 [82], and statistical support was estimated by non-parametric bootstrap [83], as described previously [67]. The model TVMef+I + G was determined as the best-fit model of substitution for codon positions 1, 2, and 3, using Partition-Finder v.1.1.1 [84], and was used for ML reconstruction.

### Statistical analysis of molecular evolution

To investigate the presence and type of selection acting on the melanopsin gene of diurnal and nocturnal snakes we applied a codon-based method, using the Codeml program from PAML v.4.7 [85]. We estimated the ratio of nonsynonymous (dN) to synonymous (dS) substitutions using random-sites models, branch models, branch-site models, and clade (CmC) models [85–88]. The dN/dS ratio (ω) indicates the type and magnitude of selection, where ω < 1 indicates purifying selection, ω ~ 1 indicates neutral evolution, and ω > 1 indicates positive selection [85, 89]. Branch, branch-site and clade models allow appointing specific branches of interest as “foreground” and compare their ω rates with the ω estimated for the “background” branches [85]. Branch-site and CmC models were applied to test for divergence along specific branches of the tree [87]. Branch model allows only a single class of sites, and thus tests for average differences between clades. CmC allows two classes of sites across the tree to evolve conservatively (0 < ω < 1) and neutrally (ω = 1), while a third site class is free to evolve differently among two or more partitions. The CmC null model, M2a_rel, does not allow ω to diverge in the foreground clade [88]. Branch-site and random-sites models allow ω to vary among codon sites and to detect sites potentially under positive selection.

Likelihood ratio tests (LRTs) were used to compare competing models of evolution. The LRT statistic was computed as 2log likelihood difference between the two models and was tested against the χ^2^ distribution, where the degrees of freedom equal the difference between the numbers of parameters in the two nested models [85]. Bayesian information criterion (BIC) was also used to compare the models, and was computed as − 2 l + K log n, where K is the number of estimated parameters and n is the sample size. The lowest BIC score indicated the best model [90–92].

Branch model was applied to a broad data set including *Opn4x* and *Opn4m* melanopsin sequences from different vertebrate groups (Fig. 1; Additional file 1: Table S2; Sequences alignment: Additional file 3) to investigate the overall evolutionary patterns of *Opn4x* and *Opn4m* genes. The null branch model assumes the same ω ratio for all branches of the tree. We applied a two-partition model that assumes a ω_0_ for the *Opn4m* and a ω_1_ for the *Opn4x*, and a three-partition model, that assumes a ω_0_ for *Opn4m*, a ω_1_ for *Opn4x*, and a ω_2_ for the snake branch.

The random-site, branch-site and CmC models were applied to a smaller data set containing only the melanopsin sequences of snakes (Additional file 4). For those analyses, we used a snake phylogenetic tree congruent with that published previously (Fig. 2) [61]. Random-sites (M0, M1a, M2a, M2a_rel, M3, M7, M8a, and M8) [85, 88] were used to determine the overall selective patterns and to test for heterogeneous selection pressure among codon sites across all branches of the snake tree. LRT comparisons between random site models were used to test for variation in ω among sites (M3 vs. M0) and the presence and proportion of positively selected sites (M2a vs. M1a, M8 vs. M7, and M8 vs. M8a) [85, 93]. When LRTs were significant for positive selection, Bayes Empirical Bayes (BEB) was used to estimate posterior probabilities for site classes and identify amino acid sites under positive selection [85].

CmC models were designed to test two different hypothesis on the evolution of melanopsin of snakes, one based on the species phylogeny, and the other based on the species daily activity pattern (Fig. 2). For the phylogenetic hypothesis, we applied the following partition tests: a two-partition (Henophidian x Caenophidian snakes), a three-partition (Henophidian x Viperdiae x Colubridae+Elapidae) and a four-partition model (Henophidian x Viperdiae x Colubridae x Elapidae). For daily activity pattern hypothesis, we tested a two-partition model, isolating all diurnal and all nocturnal species in separated clades, and compared to a three-partition model, which isolated the henophidian species *Python bivitatus* in a separate clade (Henophidia x Diurnal x Nocturnal), in order to test for divergences among the nocturnal henophidian and caenophidian lineages. Nested models were compared using LRT, and BIC was used to compare non-nested models, and determine whether diurnal or nocturnal habits display greater influence on melanopsin divergence than species relationships.

To analyze whether diurnal or nocturnal snake lineages might have experienced positive selection on any codon site, we used branch-site models [85] and implemented Model A (Model = 2, NSsites = 2), as an extension of the site-specific “neutral” model (M1) of Nielsen and Yang [94]. We also investigated for positive selected sites specifically in each snake family (Viperidae, Elapidae and Colubridae), by running two-partition branch-site models isolating each family as a foreground branch. The null models are the same as for Model A but with ω_2_ fixed at 1 for foreground branches. The proportions p_0_ and p_1_, as well as the ratio ω_2_, were estimated by maximum likelihood [93, 94].

### RNA in situ hybridization

Whole eyes of two individuals of the diurnal *T. dorsatus*, were collected and fixed in 4% paraformaldehyde (PFA) diluted in phosphate-buffered saline (PBS) (pH 7.2) treated with diethyl pyrocarbonate (DEPC) (Sigma-Aldrich, Darmstadt, Germany), for 12 h, at 4^o^ C. Eyes were cryoprotected in 30% sucrose solution diluted in PBS-DEPC for 72 h, embedded in OCT (Tissue Tek Sakura Finetek Europe) and cryosectioned at -25^o^ C. Radial sections at 12-μm thicknesses were obtained in cryostat (CM1100 Leica, Nussloch, Germany) and collected onto Superfrost Plus microscope slides (VWR, UK).

Antisense and sense digoxigenin (DIG)-labelled RNA probes were synthesized by in vitro transcription using the SP6/T7 DIG RNA Labelling Kit (Roche, UK), following manufacturer’s instruction. For antisense RNA probes, the T7 RNA polymerase promoter sequence 5′ TAATACGACTCACTATAGGGAGA was added to the reverse primer, and for sense probes, to the forward primer. Sense probes were used as a control for nonspecific staining. cDNA of *T. dorsatus* was used as PCR templates for generating 750 bp DIG-labeled RNA probes, synthesized with the primer pair OPN4_Oph_Fw2–5′ TCCTGGTCTCCGTATGCT and OPN4_Oph_Rv2–5′ GCCTTCCTTTGAGTAACAGGT. Amplicons were verified in a 1.2% agarose gel.

Retinal sections were post-fixed in PFA%-DEPC for 5 min, permeabilized with proteinase K (0.1 μg/ml) diluted in TE buffer (pH 8)-DEPC, for 15 s at room temperature (RT), washed in TE buffer for 15 s and prehybridized with ULTRAhyb® Ultrasensitive Hybridization Buffer (Ambion, UK) for 1 h, at hybridization temperature. Slides were incubated with DIG-labelled riboprobes, diluted in preheated ULTRAhyb® Ultrasensitive Hybridization Buffer (1 μg/ml), covered with hybridization coverslip (Thermo Fischer Scientific) overnight, at 72^o^ C, in a humidified chamber. Coverslips were removed in pre-heated saline-sodium citrate buffer (0.2 x SSC), and slides were washed three times in 0.2 x SSC at 72^o^ C, and then twice in TBST buffer, at RT. Slides were blocked with 10% normal goat serum (NGS) (Sigma-Aldrich), diluted in TBST for 1 h and incubated overnight at 4^o^ C, in a humid chamber, with anti-digoxigenin antibody conjugated to alkaline phosphate (AP) (Roche, UK) (diluted 1:1000 in 1% NGS in TBST buffer). Hybridized probes were detected using the BCIP/NBT (Roche). Slides were mounted in 50% glycerol diluted in PBS and visualized in a Leica DM6B microscope (Leica Microsystems, Wetzlar, Germany). Images were obtained with a 40x (0.8 NA) objective, using the software LAS X Core (Leica Microsystems, Wetzlar, Germany).

## Additional files

Additional file 1: **Tables S1-S5.** (DOCX 31 kb)

Additional file 2: Amino acid alignment of the melanopsin sequences of snakes and the lizard *Podarcis sicula* (GenBank: DQ013043.2). Horizontal bars show the location of the seven transmembrane domains predicted for the snake *Python bivitattus* (GenBank: XM_007429400.1). The gray boxes indicate the common features of melanopsins. (TIF 4171 kb)

Additional file 3: Nucleotide alignment of the *Opn4x* and *Opn4m* melanopsin sequences used for branch model analysis. (PDF 43 kb)

Additional file 4: Nucleotide alignment of melanopsin sequences of snakes used for random-site, branch-site and CmC models. (PDF 21 kb)

## Data Availability

Sequences generated in this study were deposit at the GenBank® NIH genetic sequence database (accession numbers MN241125- MN241142).

## Abbreviations

BEB: Bayes Empirical Bayes
BIC: Bayesian information criterion
DEPC: Diethyl pyrocarbonate
DIG: Digoxigenin
dN: Nonsynonymous substitutions
dS: Synonymous substitutions
ipRGC: Intrinsically photosensitive retinal ganglion cells
ISH: In situ hybridization
LRT: Likelihood ratio test
ML: Maximum Likelihood
NGS: Normal goat serum
*Opn4m*: Mammalian-like melanopsin gene
*Opn4x*: Xenopus-like melanopsin gene
PBS: Phosphate-buffered saline
PCR: Polymerase chain reaction
PFA: Paraformaldehyde
RT: Room temperature
SSC: Sodium citrate buffer
