# Supplementary material for: Characterization of the melanopsin gene (Opn4x) of diurnal and nocturnal snakes
Source: BMC Evol Biol. 2019 Aug 28;19:174. doi: 10.1186/s12862-019-1500-6 (PMC6714106; doi:10.1186/s12862-019-1500-6)
Supplement: Supplementary file 1 — Tables S1-S5. (DOCX 31 kb) [file 12862_2019_1500_MOESM1_ESM.docx]

**Characterization of the melanopsin gene (*Opn4x*) of diurnal and nocturnal snakes.**

Hauzman, E.; Kalava, V; Bonci, D.M.O; Ventura, D.F.

**Additional File 1: Tables S1-S5.**

Table S1. Snake species sequenced in this study and respective GenBank accession numbers.

| **Family** | **Subfamily** | **Species** | **GenBank Acesison Number** |
| --- | --- | --- | --- |
| Colubridae | Colubrinae | *Chironius bicarinatus* | MN241126 |
|  | Dipsadinae | *Dipsas indica* | MN241128 |
|  |  | *Sibynomorphus mikanii* | MN241138 |
|  |  | *Sibynomorphus neuwiedi* | MN241139 |
|  |  | *Erythrolamprus aesculapii* | MN241131 |
|  |  | *Erythrolamprus miliaris* | MN241132 |
|  |  | *Oxyrhopus guibei* | MN241136 |
|  |  | *Helicops modestus* | MN241133 |
|  |  | *Echinanthera cephalostriata* | MN241129 |
|  |  | *Echinanthera undulata* | MN241130 |
|  |  | *Taeniophallus persimilis* | MN241140 |
|  |  | *Philodryas patagoniensis* | MN241137 |
|  |  | *Tomodon dorsatus* | MN241142 |
|  |  | *Thamnodynastes hypoconia* | MN241141 |
| Elapidae |  | *Micrurus corallinus* | MN241134 |
|  |  | *Micrurus lemniscatus* | MN241135 |
| Viperidae |  | *Bothrops jararaca* | MN241125 |
|  |  | *Crotalus durissus terrificus* | MN241127 |

| **Gene** | **Vertebrate group** | **Species** | **Comon Name** | **GenBank Acesison Number** |
| --- | --- | --- | --- | --- |
| *Opn4m* | Agnatha | *Eptatretus burgeri* | Inshore hagfish | AB932627.1 |
|  |  | *Lethenteron camtschaticum* | Arctic lamprey | AB932626.1 |
|  | Fish | *Callorhinchus milii* | australian ghostshark | NM_001292116.1 |
|  |  | *Cynoglossus semilaevis* | tonguefish | XM_008321406.1 |
|  |  | *Danio rerio* | zebrafish | BC162681.1 |
|  |  |  |  | AY078161.1 |
|  |  |  |  | GQ925716.1 |
|  |  | *Erpetoichthys calabaricus* |  | XM_028793387.1 |
|  |  | *Esox lucius* | northern pike | XM_010870243.1 |
|  |  | *Gadus morhua* | atlantic cod | AF385823.1 |
|  |  | *Hippoglossus hippoglossus* | Atlantic halibut | KF941289.1 |
|  |  |  |  | KF941290.1 |
|  |  | *Ictalurus punctatus* | channel catfish | NM_001200264.1 |
|  |  |  |  | NM_001200310.1 |
|  |  | *Larimichthys crocea* | yellow croaker | XM_027275886.1 |
|  |  | *Mastacembelus armatus* | tire track eel | XM_026309362.1 |
|  |  | *Notothenia coriiceps* | black rockcod | XM_010777649.1 |
|  |  | *Rutilus rutilus* | common roach | AY226847.1 |
|  |  | *Salmo salar* | Atlantic salmon | NM_001279057.1 |
|  |  | *Stegastes partitus* | bicolor damselfish | XM_008288334.1 |
|  | Amphibians | *Xenopus laevis* | african clawed frog | DQ384639.1 |
|  |  | *Xenopus tropicalis* | western clawed frog | XM_002937570.4 |
|  | Reptiles | *Anolis carolinensis* | anole lizard | XM_008119111.1 |
|  |  | *Chelonia mydas* | green sea turtle | XM_007069593.1 |
|  |  | *Chrysemys picta bellii* | painted turtle | XM_005293970.1 |
|  |  | *Pelodiscus sinensis* | Chinese softshell turtle | XM_014578738.1 |
|  |  | *Podarcis muralis* | common wall lizard | XM_028728454.1 |
|  |  | *Terrapene mexicana* | box turtle | XM_024210165.2 |
|  |  | *Trachemys scripta elegans* | red-eared turtle | HM197714.1 |
|  | Birds | *Athene cunicularia* | burrowing owl | XM_026851875.1 |
|  |  | *Egretta garzetta* | little egret | XM_009649218.1 |
|  |  | *Empidonax traillii* | willow flycatcher | XM_027895937.1 |
|  |  | *Falco peregrinus* | peregrine falcon | XM_027785703.1 |
|  |  | *Gallus gallus* | red jungle fowl | EU124630.1 |
|  |  | *Haliaeetus leucocephalus* | bald eagle | XM_010567879.1 |
|  |  | *Meleagris gallopavo* | Wild turkey | XM_019617472.1 |
|  |  | *Opisthocomus hoazin* | hoatzin | XM_009935095.1 |
|  |  | *Pipra filicauda* | wire-tailed manakin | XM_027734622.1 |
|  |  | *Pygoscelis adeliae* | adélie penguin | XM_009334644.1 |
|  |  | *Taeniopygia guttata* | zebra finch | EU327185.1 |
|  |  | *Tinamus guttatus* | white-throated tinamou | XM_010212296.1 |
|  | Mammals | *Bos taurus* | cattle | NM_001192399.1 |
|  |  | *Canis lupus familiaris* | dog | XM_005619440.1 |
|  |  | *Dipodomys ordii* | ord's kangaroo rat | XM_013032062.1 |
|  |  | *Eptesicus fuscus* | big brown bat | XM_028156936.1 |
|  |  | *Felis catus* | cat | AY382594.2 |
|  |  | *Homo sapiens* | human | NM_033282.3 |
|  |  | *Macaca nemestrina* | pig-tailed macaque | XM_011765997.1 |
|  |  | *Monodelphis domestica* | gray short-tailed opossum | XM_001377332.3 |
|  |  | *Mus musculus* | mouse | NM_013887.2 |
|  |  | *Phyllostomus discolor* | pale spear-nosed bat | XM_028512736.1 |
|  |  | *Physeter catodon* | sperm whale | XM_007120486.3 |
|  |  | *Rattus norvegicus* | brown rat | AY072689.1 |
|  |  | *Sminthopsis crassicaudata* | fat-tailed dunnart | DQ383281.1 |
|  |  | *Spalax ehrenbergi* | palestinian blind mole-rat | AM748539.1 |
|  |  | *Tarsius syrichta* | philippine tarsier | XM_008060098.1 |

Table S2. Species used for phylogenetic analysis and for PAML branch models and respective GenBank accession numbers.

| **Gene** | **Vertebrate group** | **Species** | **Comon Name** | **GenBank Acesison Number** |
| --- | --- | --- | --- | --- |
|  | Fish | *Callorhinchus milii* | australian ghostshark | NM_001292191.1 |
| *Opn4x* |  | *Danio rerio* | zebrafish | GQ925718.1 |
|  |  |  |  | NM_001258223.1 |
|  |  | *Hippoglossus hippoglossus* | Atlantic halibut | KF941292.1 |
|  |  |  |  | KF941293.1 |
|  |  | *Salmo salar* | Atlantic salmon | NM_001279053.1 |
|  |  |  |  | JN210544.1 |
|  |  |  |  | NM_001279055.1 |
|  | Amphibians | *Xenopus laevis* | african clawed frog | NM_001085674.1 |
|  | Reptiles | *Alligator mississippiensis* | American alligator | XM_019488093.1 |
|  |  | *Anolis carolinensis* | anole lizard | XM_008111056.1 |
|  |  | *Arizona elegans* | Glossy Snake | SRP132105 |
|  |  | *Chelonia mydas* | green sea turtle | XM_027825443.1 |
|  |  | *Cemophora coccinea* | Scarlet snake | SRP132105 |
|  |  | *Gekko japonicus* | Schlegel's Japanese gecko | XM_015418685.1 |
|  |  | *Hypsiglena torquata* | night snake | SRP132105 |
|  |  | *Lampropeltis getula* | eastern kingsnake | SRP132105 |
|  |  | *Masticophis flagellum* | whip snake | SRP132105 |
|  |  | *Notechis scutatus* | Tiger snakes | XM_026672626.1 |
|  |  | *Pantherophis guttatus* | Corn snake | SRP132105 |
|  |  | *Podarcis sicula* | ruin lizard | DQ013043.2 |
|  |  | *Podarcis muralis* | common wall lizard | XM_028744000.1 |
|  |  | *Pogona vitticeps* | bearded dragon | XM_020798658.1 |
|  |  | *Pseudonaja textilis* | eastern brown snake | XM_026697858.1 |
|  |  | *Protobothrops mucrosquamatus* | brown spotted pit viper | XM_015815691.1 |
|  |  | *Python bivittatus* | burmese python | XM_007429400.1 |
|  |  | *Thamnophis sirtalis* | garter snake | XM_014063405.1 |
|  |  | *Trachemys scripta elegans* | red-eared turtle | JN815264.1 |
|  | Birds | *Gallus gallus* | red jungle fowl | NM_204625.2 |
|  |  | *Meleagris gallopavo* | Wild turkey | FJ483933.2 |
|  |  | *Taeniopygia guttata* | zebra finch | EU327186.1 |

Table S3. Positive selected sites under Random-Site Models M2a and M8 of the Snake *Opn4x* Species Tree.

| Model | np | ln L | κ | Parameters | Positive Selected Sites |
| --- | --- | --- | --- | --- | --- |
| M2a | 60 | -6,411.7 | 3.5 | ω_0_ = 0.1 (p_0_ = 0.69)  ω_1_ = 1.0 (p_1_ = 0.26)  ω_2_ = 2.45 (p_2_ = 0.05) | 230Q (0.8), 270I (0.8), 306R (0.8), 320A (0.8), 327L (0.7), 338M (0.9), 352D (0.7), 380L (0.8), 400I (0.8), 423K (1.0), 456E (0.7), 459M (0.8) |
| M8a | 59 | -6,417.7 | 3.4 | p_0_ = 0.69, p = 1.57, q = 14.77 (p_1_ = 0.31, ω = 1.0) | 46K (0.9), 79H (0.7), 87T (0.8), 177A (0.7), 230Q (0.9), 270I (0.9), 306R (0.9), 307K (0.8), 320A (0.9), 327L (0.9), 329N (0.7), 338M (1.0), 340S (0.8), 345I (0.8), 350S (0.7), 352D (0.9), 380L (0.9), 400I (0.9), 412S (0.8), 423K (1.0), 456E (0.8), 457K (0.7), 459M (0.9), 470Q (0.8), 485R (0.8), 494I (0.8) |

np, number of parameters; ln L, ln likelihood; κ, transition/transversion ratio.

Table S4. Results from Branch-site model analyses on the snakes *Opn4x* species tree

| Branch-Site models | np | Ln L | Parameters | BIC | Null | LRT | df | p | Positive Selected Sites |
| --- | --- | --- | --- | --- | --- | --- | --- | --- | --- |
| BrS_Alt  Diurnal | 60 | -6,415.9 | ω_0_ = 0.09 (p_0_ = 0.68), ω_1_ = 1.0 (p_1_ = 0.3); ω_2_ = 3.3 (p_2_ + p_3_ = 0.02) | 12,993.5 | BrS_null | 3,0 | 1 | 0.048 | 423K (0.7), 494I (0.86) |
| BrS_Null Diurnal | 59 | -6,417.8 | ω_0_ = 0.08 (p_0_ = 0.68), ω_1_ = 1.0 (p_1_ = 0.32); ω_2_ = 1.0 (p_2_ + p_3_ = 0.00) | 12,994.7 |  |  |  |  |  |
| BrS_Alt  Nocturnal | 60 | -6,412.8 | ω_0_ = 0.04 (p_0_ = 0.56), ω_1_ = 1.0 (p_1_ = 0.24); ω_2_ = 1.0 (p_2_ + p_3_ = 0.198) | 12,987.5 | BrS_null | 0.0 | 1 | 1.0 | - |
| BrS_Null Nocturnal | 59 | -6,412.8 | ω_0_ = 0.04 (p_0_ = 0.56), ω_1_ = 1.0 (p_1_ = 0.24); ω_2_ = 1.0 (p_2_ + p_3_ = 0.198) | 12,984.8 |  |  |  |  |  |
| BrS_Alt  Viperidae | 60 | -6,416.3 | ω_0_ = 0.07 (p_0_ = 0.59), ω_1_ = 1.0 (p_1_ = 0.28); ω_2_ = 1.0 (p_2_ + p_3_ = 0.13) | 12,994.3 | BrS_null | 0.0 | 1 | 1.0 |  |
| BrS_Null Viperidae | 59 | -6,416.3 | ω_0_ = 0.07 (p_0_ = 0.59), ω_1_ = 1.0 (p_1_ = 0.28); ω_2_ = 1.0 (p_2_ + p_3_ = 0.13) | 12,991.6 |  |  |  |  |  |
| BrS_Alt  Elapidae | 60 | -6,416.2 | ω_0_ = 0.08 (p_0_ = 0.67), ω_1_ = 1.0 (p_1_ = 0.31); ω_2_ = 3.6 (p_2_ + p_3_ = 0.02) | 12,994.1 | BrS_null | 1.0 | 1 | 0.31 |  |
| BrS_Null Elapidae | 59 | -6,416.7 | ω_0_ = 0.08 (p_0_ = 0.63), ω_1_ = 1.0 (p_1_ = 0.29); ω_2_ = 1.0 (p_2_ + p_3_ = 0.08) | 12,992.4 |  |  |  |  |  |
| BrS_Alt  Colubridae | 60 | -6,405.4 | ω_0_ = 0.1 (p_0_ = 0.69), ω_1_ = 1.0 (p_1_ = 0.26); ω_2_ = 3.7 (p_2_ + p_3_ = 0.05) | 12,972.5 | BrS_null | 24.9 | 1 | 0.000 | 79H (0.8), 230Q (0.9), 306R (0.95), 320A (0.8), 338M (0.97), 350S (0.8), 423K (1.0), 457K (0.9), 459M (0.9) |
| BrS_Null Colubridae | 59 | -6,417.8 | ω_0_ = 0.09 (p_0_ = 0.68), ω_1_ = 1.0 (p_1_ = 0.32); ω_2_ = 1.0 (p_2_ + p_3_ = 0.00) | 12,994.7 |  |  |  |  |  |
| BrS_Alt  Diurnal_Col | 60 | -6,413.5 | ω_0_ = 0.09 (p_0_ = 0.68), ω_1_ = 1.0 (p_1_ = 0.29); ω_2_ = 4.2 (p_2_ + p_3_ = 0.03) | 12,988.8 | BrS_null | 8.6 | 1 | 0.003 | 256Y (0.75), 457K (0.9), 459M (0.8) |
| BrS_Null Diurnal_Col | 59 | -6,417.8 | ω_0_ = 0.09 (p_0_ = 0.68), ω_1_ = 1.0 (p_1_ = 0.32); ω_2_ = 1.0 (p_2_ + p_3_ = 0.00) | 12,994.7 |  |  |  |  |  |
| BrS_Alt  Noct_Col | 60 | -6,415.9 | ω_0_ = 0.08 (p_0_ = 0.67), ω_1_ = 1.0 (p_1_ = 0.31); ω_2_ = 4.4 (p_2_ + p_3_ = 0.02) | 12,993.5 | BrS_null | 3.3 | 1 | 0.069 | 290N (0.8), 318I (0.7), 338M 0.7 |
| BrS_Null Noct_Col | 59 | -6,417.5 | ω_0_ = 0.08 (p_0_ = 0.66), ω_1_ = 1.0 (p_1_ = 0.31); ω_2_ = 1.0 (p_2_ + p_3_ = 0.03) | 12,994.1 |  |  |  |  |  |

np, number of parameters; ln L, ln likelihood; BIC, Bayesian information criterion, LRT, likelihood ratio test; df, degrees of freedom.

Table S5. Primers used to amplify the melanopsin partial coding sequences of snakes.

| Primers | Sequence | Annealing Temperature |
| --- | --- | --- |
| OPN4_Pyt_Fw1 | 5’ TTGGTTGTATTGGAATTATAGGG | 57.1 |
| OPN4_Pyt_Rv1 | 5’ CACAAGCATATGGAGACCAG | 56.7 |
| OPN4_Pyt_Fw2 | 5’ CTGGCTATGCCAGGATCT | 56.2 |
| OPN4_Pyt_Rv2 | 5’ GGTGAAGCCTTCCTTTGA | 56.2 |
| OPN4_Oph_Fw1 | 5’ GCTCAGGCAAAGAGCAAT | 56.6 |
| OPN4_Oph_Rv1 | 5’ CGGAGACCAGGATACAACA | 57.0 |
| OPN4_Oph_Fw2 | 5’ TCCTGGTCTCCGTATGCT | 56.6 |
| OPN4_Oph_Rv2 | 5’ GCCTTCCTTTGAGTAACAGGT | 57.5 |
| OPN4_Lmil_Fw1 | 5’ TCTCCTTGTCCTCTATGCATTTTAC | 60.0 |
| OPN4_Lmil_Rv1 | 5’ GTTCTCCTGTAACTTGGGTGAATAA | 59.8 |
| OPN4_Lmil_Fw2 | 5’ GTAGCCATTGTGGTTTACGTTGTAT | 60.4 |
| OPN4_Lmil_Rv2 | 5’ CAGCGTTGTCTTCCTCTATATTTTC | 59.7 |
| OPN4_Dips_Fw1 | 5’ TTGGAATTATAGGGAATCTCCTTG | 59.7 |
| OPN4_Dips_Rv1 | 5’ AGCATATGGAGACCAGGATACAA | 59.9 |
| OPN4_Dips_Fw2 | 5’ GCTGGTTATGCCAGAATCTTAAC | 59.2 |
| OPN4_Dips_Rv2 | 5’ CAGAGTGGTTTCTGAGGTAGGAA | 59.8 |
| OPN4m_Ts_Fw | 5’ GAACGCTGGGTAACTTCCTG | 59.7 |
| OPN4m_Ts_Rv | 5’ TGGAACGTCTGGTTGAGAGA | 59.4 |
