# Supplementary figures and images for: Characterization of the melanopsin gene (Opn4x) of diurnal and nocturnal snakes
Source: BMC Evol Biol. 2019 Aug 28;19:174. doi: 10.1186/s12862-019-1500-6 (PMC6714106; doi:10.1186/s12862-019-1500-6)

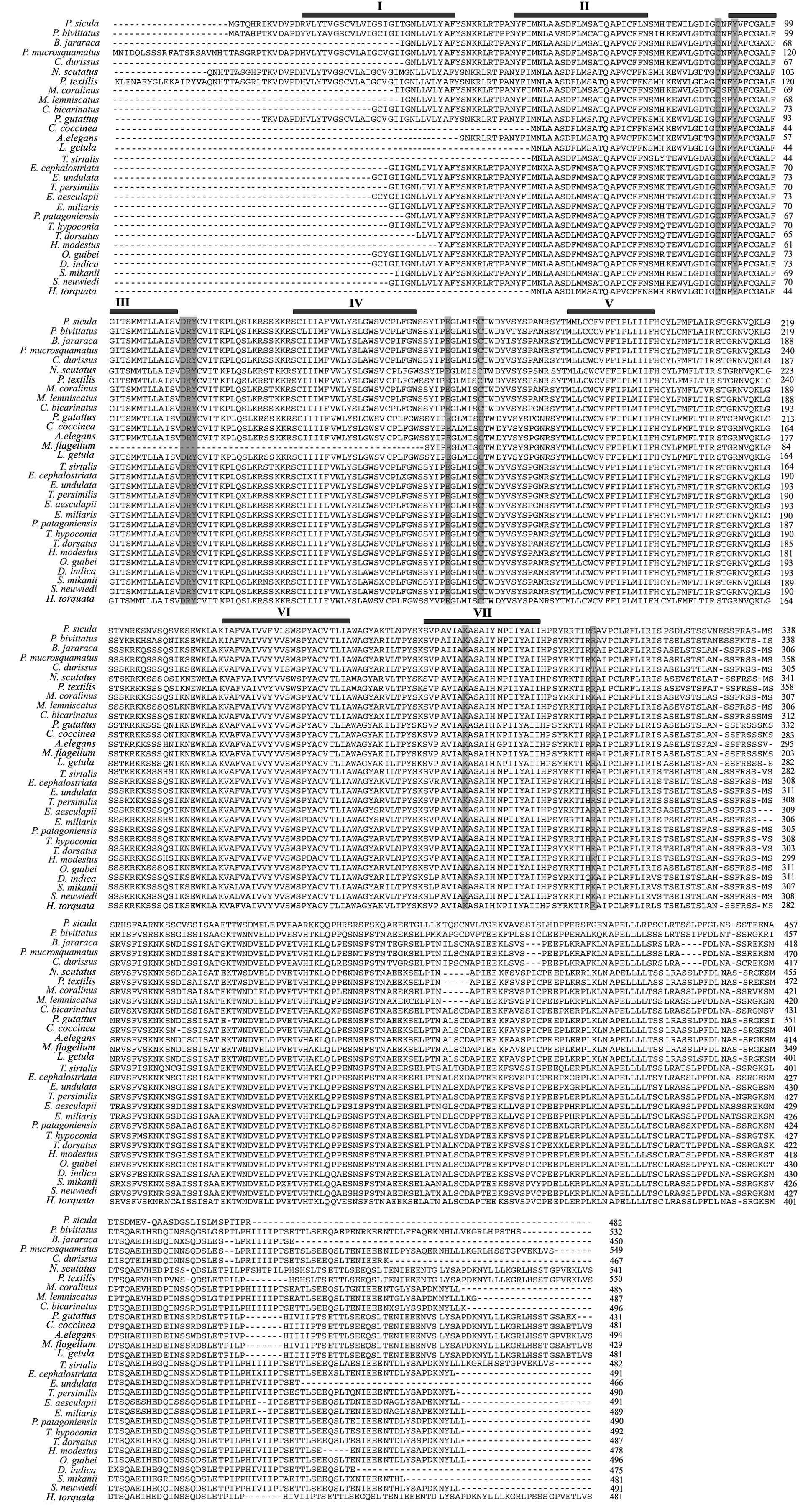

Supplement: Supplementary file 2 — Amino acid alignment of the melanopsin sequences of snakes and the lizard Podarcis sicula (GenBank: DQ013043.2). Horizontal bars show the location of the seven transmembrane domains predicted for the snake Python bivitattus (GenBank: XM_007429400.1). The gray boxes indicate the common features of melanopsins. (TIF 4171 kb) [file 12862_2019_1500_MOESM2_ESM.tif]
