# Supplementary material for: Characterization of the melanopsin gene (Opn4x) of diurnal and nocturnal snakes
Source: BMC Evol Biol. 2019 Aug 28;19:174. doi: 10.1186/s12862-019-1500-6 (PMC6714106; doi:10.1186/s12862-019-1500-6)
Supplement: Supplementary file 3 — Nucleotide alignment of the Opn4x and Opn4m melanopsin sequences used for branch model analysis. (PDF 43 kb) [file 12862_2019_1500_MOESM3_ESM.pdf]

Hauzman et al. Additional File 3

```
               ....|....| ....|....| ....|....| ....|....| ....|....| ....|....| ....|....| ....|....| ....|....| ....|....| ....|....|
                        10         20         30         40         50         60         70         80         90        100        110
Felis000       CtGgGcAcAg TgAtCcTgCt GgTgGgGcTc AcGgGgAtTc TgGgCaAtCt GaTgGtCaTc TaTaCtTtCt GcAgG----- ---------- ---------- ----------
Gallus001      GTGGGGACGT GTGTTCTTGT TATTGGCTCC ATTGGCATCA TAGGAAACCT CCTAGTTCTC TATGCATTTT ACAGC----- ---------- ---------- ----------
Gallus002      ATTGGAACAG TCATTCTTAT CGTGGGGATC ACAGGAACTC TGGGTAATTT CCTGGTCATC TATGCTTTCT GCAGG----- ---------- ---------- ----------
Gadus003       GTGGCTTTCT TCGTCTCCGT CATCGGCACC CTGGGCGTCA CGGGGAACGC CCTCGTGCAG TTCGCCTTCT ACAGC----- ---------- ---------- ----------
Gadus_004      ATCGCCTTCT TCGTGCTCAT CATCGGAGTG GTCGGTGTGA CCGGCAACGC CCTGGTCATG TACGCCTTTT TGTGT----- ---------- ---------- ----------
Sminth005      ATTGGTGCTA CCATCTTGGT TGTGGGGTTC ACAGGAGTGC TGGGGAACCT ACTGGTCATC TACACCTTCT GCAGG----- ---------- ---------- ----------
Homo006  C     TGGGCACAGT GATCTTGCTG GTGGGACTCA CGGGGATGCT GGGCAACCTG ACGGTCATCT ATACCTTCTG CAGG------ ---------- ---------- ----------
Mus007  CT     AGGCACGGTG ATCCTGCTGG TGGGACTCAC AGGGATGCTG GGCAATCTGA CGGTCATCTA CACCTTCTGC AGG------- ---------- ---------- ---------A
Spalax008      CTGGGCACAG TGATCCTGCT GGTGGGGCTC ACGGGGATGC TGGGCAATCT GATAGTCATC TATACCTTCT GCAGG----- ---------- ---------- ----------
Monode009      ATTGGTGCTA CCATCCTGGC TGTGGGCTTC ACAGGAGTGC TGGGGAACCT GTTGGTCATC TACACATTCT GCAGG----- ---------- ---------- ----------
Rattus010      CTAGGCACGG TGATCCTGCT GGTGGGACTC ACAGGGATGC TGGGTAACCT GACAGTCATC TACACCTTCT GCAGG----- ---------- ---------- ----------
Podarc011      GTAGGCTCCT GTGTTCTTGT CATTGGTTCT ATTGGAATCA CAGGAAATCT TCTTGTCCTC TATGCATTTT ACAGC----- ---------- ---------- ----------
Trache012      ATTGGAACTG TCATTCTGGT GGTGGGGATC ACCGGAACGC TGGGTAACTT CCTGGTCATC TATGCGTTCT GCAGG----- ---------- ---------- ----------
Xenopu013      ATTGGGAGCT TCATACTGAT AATCGGTTCT GTGGGAATTA TAGGAAACAT GTTGGTTCTC TATGCATTTT ACAGA----- ---------- ---------- ----------
Danio014       ATTGGTGCCG TTATCCTCAC AGTTGGCATT ACTGGCATGC TGGGCAACTT CCTGGTCATC TATGCATTCA GCAGG----- ---------- ---------- ----------
Danio_015      ATCGGCTCTG TTATCTTAAT AGTCGGCATC ACCGGAGTGA TTGGAAATGC ACTGGTGGTC TATGTATTCT GCCGG----- ---------- ---------- ----------
Anolis016      ATTGGGGCTG TCATTCTTGT AGTGGGAATC ACAGGAACTC TTGGCAACCT TCTAGTCATC TATGTTTTTT TCAGG----- ---------- ---------- ----------
Bos017  CT     GGGCACGGTG ATCCTGCTGG TGGGACTTAC GGGGATGCTG GGAAATCTGA CGGTCATCTA TACCTTCTGC AGG------- ---------- ---------- ---------A
Callor018      TTTGGCAGCT GCATTATTGT GATTGGCACC ATTGGAGTCT TGGGGAATCT CCTGGTACTT TATGCAGTTT ACAGC----- ---------- ---------- ----------
Canis019       CTGGGCACCG TGATCCTGCT GGTGGGGCTC ACGGGGATGC TGGGCAACCT GATGGTCATC TATACCTTCT GCAGG----- ---------- ---------- ----------
Cynogl020      ATTGGCTCCG TCATCCTCGC CATCGGCATT ACCGGCATGG TTGGCAACTT CCTTGTCATT TATGCCTTCA GCAGG----- ---------- ---------- ----------
Dipodo021      CTGGGCACGG TGATCCTGCT GGTGGGGCTC ACGGGGATGC TGGGAAATCT GACCGTCATC TATACCTTTT GCAGG----- ---------- ---------- ----------
Esox022  A     TAGGGGCCGT CATCCTGACT ATTGGCATCA CTGGTATGGT GGGGAACTTT CTGGTCATCT ATGCCTTCAG CAGG------ ---------- ---------- ----------
Haliae023      ATTGGAGTAG TCATTCTTAT AGTGGGGATC ACAGGAACTC TGGGTAATTT CCTGGTCATC TATGCTTTCT GCAGG----- ---------- ---------- ----------
Macaca024      CTGGGCACAG TGATCTTGCT GGTGGGACTC ACGGGGATGC TGGGCAACCT GACGGTCATC TATACTTTCT GCAGGTCT-- -GTGCTTCAT GGAGTCACTG TGATGATGCA
Nototh025      ATTGGTGTGG TCATCCTGAT CGTTGGCATC ACGGGCATGC TGGGAAACCT CCTGGTTATA TATGCTTTCT GCAGG----- ---------- ---------- ----------
Pygosc026      ATTGGAGTAG TCATTCTTAT AGTGGGGATC ACAGGAACTC TGGGTAATTT TCTGGTCATC TATGCTTTCT GCAGG----- ---------- ---------- ----------
Rutilu027      ATCGGCGCTG TCATTCTAAT AGTTGGCATC ACCGGAGTGA TTGGAAACGC ACTGGTGATC TACGTGTTCT GCCGG----- ---------- ---------- ----------
Taenio028      ---------- ---------- ---------- ---------- ---------- ---------- ---------- ---------- ---------- ---------- ----------
Taenio029      ---------- ---------- ---------- ---------- ---------- ---------- ---------- ---------- ---------- ---------- ----------
Tarsiu030      CTGGGCACAG TGATCTTGCT GGTTGGGCTC ACAGGGATGC TGGGCAACCT GACAGTCATC TATACCTTCT GCAGGTGC-- -CTGGTTGGT GGTGTCACTA TGATGATTCA
Tinamu031      ATTGGAGGCG TCATTCTTGC CGTGGGAATC ACTGGGACTC TGGGCAATTT CTTGGTCATC TACACTTTCT GCAGG----- ---------- ---------- ----------
Trache032      ---------- ---------- ---------- ---------- ---------- ---------- ---------- ---------- ---------- ---------- ----------
Xenopu033      GTTGGTGCTG TTATCCTTGC AGTAGGAATC ACAGGCATGC TGGGAAATTT TCTAGTCATC TATGCTTTCT GCAGA----- ---------- ---------- ----------
Bjar_O034      ---------- ---------- ---------- ---------A TAGGGAATCT CCTTGTCCTC TATGCATTTT ACAGC----- ---------- ---------- ----------
Cbic_O035      ---------- ---------- --TTGGTTGT ATTGGAATTA TAGGGAATCT CCTTGTCCTC TATGCATTTT ACAGC----- ---------- ---------- ----------
Cdur_O036      ---------- ---------- ---------- ---------- --GGGAATCT CCTTGTCCTC TATGCATTTT ACAGC----- ---------- ---------- ----------
Dips_O037      ---------- ---------- --TTGGTTGT ATTGGAATTA TAGGGAATCT CCTTGTCCTC TATGCATTTT ACAGC----- ---------- ---------- ----------
Eceph_038      ---------- ---------- ---------- -TTGGAATTA TAGGGAATCT CATTGTCCTC TATGCATTTT ACAGC----- ---------- ---------- ----------
Eund_O039      ---------- ---------- --TTGGTTGT ATTGGAATTA TAGGGAATCT CATTGTCCTC TATGCATTTT ACAGC----- ---------- ---------- ----------
Ery_OP040      ---------- ---------- --TTGGTTGT TATGGAATTA TAGGGAATCT CCTTGTCCTC TATGCATTTT ACAGC----- ---------- ---------- ----------
Hel_OP041      ---------- ---------- ---------- ---------- ---------- ---------C TATGCATTTT ACAGC----- ---------- ---------- ----------
Lmil_O042      ---------- ---------- ---------- --TGGAATTA TAGGGAATCT CCTTGTCCTC TATGCATTTT ACAGC----- ---------- ---------- ----------
Mcor_O043      ---------- ---------- ---------- -----AATTA TAGGGAATCT CCTTGTCCTC TATGCATTTT ACAGC----- ---------- ---------- ----------
Mlem_O044      ---------- ---------- ---------- ---------A TAGGGAATCT CCTTGTCCTC TATGCATTTT ACAGC----- ---------- ---------- ----------
Oguib_045      ---------- ---------- ---TGGTTGT TATGGAATTA TAGGGAATCT CCTTGTCCTC TATGCATTTT ATAGC----- ---------- ---------- ----------
Arizon046      ---------- ---------- ---------- ---------- ---------- ---------- ---------- ---------- ---------- ---------- ----------
Cemoph047      ---------- ---------- ---------- ---------- ---------- ---------- ---------- ---------- ---------- ---------- ----------
Hypsig048      ---------- ---------- ---------- ---------- ---------- ---------- ---------- ---------- ---------- ---------- ----------
Lampro049      ---------- ---------- ---------- ---------- ---------- ---------- ---------- ---------- ---------- ---------- ----------
Pgutat050      --AGGCTCTT GTGTTCTGGC CATTGGTTGT GTTGGAATTA TAGGAAATCT CCTTGTACTC TATGCATTTT ACAGC----- ---------- ---------- ----------
Mastic051      ---------- ---------- ---------- ---------- ---------- ---------- ---------- ---------- ---------- ---------- ----------
Ppat_O052      ---------- ---------- ---------- ---------- --GGGAATCT CCTTGTCCTC TATGCATTTT ACAGC----- ---------- ---------- ----------
Smik_O053      ---------- ---------- ---------- ----GAATTA TAGGGAATCT CCTTGTCCTC TATGCATTTT ACAGC----- ---------- ---------- ----------
Snew_O054      ---------- ---------- ---------- -ATGGAATTA TAGGGAATCT CCTTGTCCTC TATGCATTTT ACAGC----- ---------- ---------- ----------
Tae_OP055      ---------- ---------- ---------- --TGGAATTA TAGGGAATCT CATTGTCCTC TATGCATTTT ACAGC----- ---------- ---------- ----------
Tham_O056      ---------- ---------- ---------- --AGGAATTA TAGGGAATCT CATTGTCCTC TATGCATTTT ACAGC----- ---------- ---------- ----------
Tdor_O057      ---------- ---------- ---------- ---------- ------ATCT CCTTGTCCTC TATGCATTTT ACAGC----- ---------- ---------- ----------
Anolis058      ---------- ---------- ---------- ---------- ---------- ---------- ---------- ---------- ---------- ---------- ----------
Pogona059      ---GGCTGGC TGGCCGGCCG GCCGGCATCT GGGTTGGCGC GAGCAGCATG GCAGACCCCG GGGCGCATGA GCAGC----- ---------- ---------- ----------
Protob060      GTAGGCTCTT GTGTCCTGGC CATTGGTTGT GTTGGAATTA TAGGAAATCT CCTTGTCCTC TATGCATTTT ACAGC----- ---------- ---------- ----------
Thamno061      ---------- ---------- ---------- ---------- ---------- ---------- ---------- ---------- ---------- ---------- ----------
Alliga062      GTTGGAACCT GTATTCTTGT AATCGGATCC GTTGGAATTA TAGGCAACCT CTTAGTTATT TATGCATTTT ACAGC----- ---------- ---------- ----------
Athene063      ATCGGAGTAG TCATTCTTAT CGTGGGGATC ACGGGAACTC TGGGCAATTT TCTGGTCATC TATGCTTTCT GCAGG----- ---------- ---------- ----------
Callor064      ATCGGAGCCA CCATCCTGGC CGTGGGCGTG ACCGGGATGG TGGGCAACTT CCTGGTCATC TATGCATTCC TCAGA----- ---------- ---------- ----------
Chelon065      ATTGGAACTG TCATTCTGAT GGTGGGGATC ACCGGAACGC TGGGTAACTT CCTGGTCATC TATGCTTTCT GCAGG----- ---------- ---------- ----------
Chelon066      ACTGGAACCT GTGTTCTTAT AATTGGATCT ATTGGAGTTA CCGGAAACCT TTTGGTTTTT TATGCATTTT ACAGC----- ---------- ---------- ----------
Chryse067      ATTGGAACTG TCATTCTGGT GGTGGGGATC ACCGGAACGC TGGGTAACTT CCTGGTCATC TATGCGTTCT GCAGG----- ---------- ---------- ----------
Danio_068      ATCGGCTCTG TTATCTTAAT AGTCGGCATC ACCGGAGTGA TTGGAAATGC ACTGGTGGTC TATGTATTCT GCCGG----- ---------- ---------- ----------
Danio_069      GTGGCGTTTT TTGTGGCCGT CATCGGGGCT CTAGGAGTGA TCGGAAACCT GCTGGTCATG TACGCCTTCT TTAGT----- ---------- ---------- ----------
Danio_070      ATCGGCTCTG TCATTCTGGC GGTGGGCATC ACCGGCATGG TGGGAAACCT TTTGGTCATG TATGCCTTCT GCAAG----- ---------- ---------- ----------
Danio_071      ATCGCCTTCC TCATCCTCAT CATCGGCACT TTGGGAGTTT CTGGAAACGC TCTGGTCATG TTCGCCTTCT ACAGT----- ---------- ---------- ----------
Egrett072      ATTGGAGTAG TCATTCTTAT AGTGGGGATC ACAGGAACGC TGGGTAATTT TCTGGTCATC TATGCTTTCG GCAGG----- ---------- ---------- ----------
Empido073      ATTGGAATAG TCATTCTTAT AGTGGGGATC ACAGGAACTC TGGGTAATTC CCTGGTCTTC TATGCTTTCT GCAGG----- ---------- ---------- ----------
Eptatr074      ATCGGTGCTG TGGTCCTCAT CATCGGAATA ATTGGCTGCA TCGGAAACGC TGTCGTGATG TTTGCGTTTT GTCGG----- ---------- ---------- ----------
Eptesi075      CTGGGCACAG TGATCCTGCT GGTGGGCCTC ACCGGGATGC TGGGCAATCT GACCGTCATC TATACCTTTT GCAGG----- ---------- ---------- ----------
Erpeto076      ATTGGAGCTG TGATTCTTGC AGTGGGCATC ACAGGCATTC TGGGAAACTT ACTTGTTATA TATGCATTTA GCAGA----- ---------- ---------- ----------
Falco_077      ATTGGAGTAG TCATTCTTAT AGTGGGGATC ACAGGAAGTC TGGGTAATTT CCTGGTCTTC TATGCTTTTT GCAGG----- ---------- ---------- ----------
Gekko_078      GTAGGCTCCT GTGTTCTTGT AATTGGTACT ATTGGAATTA CAGGAAATCT TCTGGTTCTC TATGCATTTT ACAGC----- ---------- ---------- ----------
Hippog080      ATTGGCTCTG TCATCCTCGC CATTGGCATC ACCGGCATGG TGGGAAACTT CCTGGTCATT TACGCCTTCA GCAGG----- ---------- ---------- ----------
Hippog081      ATTGGTGTTG TCATCATGGC TGTGGGCATC ACAGGCATGC TGGGAAACTT CCTGGTCATA TATGCTTTCT GCAGG----- ---------- ---------- ----------
Hippog082      GTGGCCTTCT TTGTCTTCGT GATTGGGACA GTAGGGGTCA CCGGGAATGC CTTGGTCATG TACGCCTTCT TCTGC----- ---------- ---------- ----------
Hippog083      GTTGCTCTGT TCGTCTTCGT CATCGGGACG CTGGGCATCA CCGGCAATGC TCTGGTTATG TTCGCCTTCT ATAGT----- ---------- ---------- ----------
Ictalu084      ATCGGCACAT TTATCTTAAT CATTGGCATC ACTGGCATGG TGGGCAACTT TCTGGTCATG TACGCCTTCT GCAAG----- ---------- ---------- ----------
Ictalu085      ATCGGGTCGG TCATCCTCAT CGTGGGGATC ACAGGCGTAA TAGGAAATGC GCTGGTCATC TATGTGTTCT GCCGT----- ---------- ---------- ----------
Larimi086      ATCGGCTCCG TCATCCTCGC CATCGGCATC ACCGGCATGG TCGGCAACTT CCTTGTCATT TATGCCTTCT GCAGG----- ---------- ---------- ----------
Lethen087      ATCGGCTCGG TGGTCATCGC CATCGGCTTC ACAGGGATCA TCGGCAACTT CCTCGTCATT TACGTCTTCT GCAGG----- ---------- ---------- ----------
Mastac088      ATCGGCTCCG TCATCCTCGC CATCGGCATC ACCGGCTTGG TCGGAAACCT CCTGGTCATA TACGCCTTTA GCAGG----- ---------- ---------- ----------
Meleag089      ATAGGGACGT GCGTTCTTGT TATTGGTTCC ATCGGCATCA TAGGAAACCT CCTGGTTCTC TATGCATTTT ACAGC----- ---------- ---------- ----------
Meleag090      ATTGGAACAG TCATTCTTAT CGTGGGAATC ACAGGAACTC TGGGTAATTT CCTGGTCATC TATGCTTTCT GCAGG----- ---------- ---------- ----------
Notech091      GTAGGCTCTT GTGTTCTGGC CATTGGTTGT GTTGGAATTA TGGGAAATCT CCTTGTCCTC TATGCATTTT ACAGC----- ---------- ---------- ----------
Opisth092      ATTGGAGTAG TCATTCTTAT AGTGGGGATC ACAGGAACGC TGGGCAATTT CCTGGTCATC TATGCTTTCG GCAGG----- ---------- ---------- ----------
Pelodi093      ATTGGAACTG TCATTCTGGT GGTGGGGATC ACCGGAGCAC TGGGTAACTT CCTCGTCATC TATGCTTTCT GCAGG----- ---------- ---------- ----------
Phyllo094      CTAGGCACAG TGGTCCTGCT GGTGGGCCTC ACAGGAATGC TGGGCAATCT GACAGTCATC TATACCTTTT GCAGG----- ---------- ---------- ----------
Physet095      CTGGGCACGG TGATCCTGCT GGTGGGGCTC ACAGGGATGC TGGGAAATCT GACGGTCATC TATACCTTCT GCAGTTGTGA TTTACTTCAT GGAGTCAATG TGATGATTAA
Pipra_096      ATTGGAATAG TCATTCTTAT AGTGGGGATC ACGGGAACTC TGGGTAATTT CCTGGTCTTC TATGCTTTCT GCAGG----- ---------- ---------- ----------
Podarc097      GTAGGCTCCT GTGTTCTTGT CATTGGTTCC ATTGGAATCA CAGGAAATCT CCTTGTCCTC TATGCATTTT ACAGC----- ---------- ---------- ----------
Podarc098      ATCGGGGCTG TCATTCTCGC AGTGGGGATC ACAGGAACTC TTGGTAACCT CCTGGTCATC TTTGCTTTCT GCAGG----- ---------- ---------- ----------
Pseudo099      GTAGGCTCTT GTGTTCTGGC CATTGGTTGT GTTGGAATTA TAGGAAATCT CCTTGTCCTC TATGCATTTT ACAGC----- ---------- ---------- ----------
Python100      GTAGGCTCTT GTGTTCTGGT CATTGGTTGT ATTGGAATTA TAGGGAATCT CCTTGTTCTC TATGCATTTT ACAGC----- ---------- ---------- ----------
Salmo_101      ATAGGGGTAG TCATTCTGGC TATCGGCATC ACCGGCATGG TGGGCAACTT CCTGGTTATC TATGCCTTCA GCAGG----- ---------- ---------- ----------
Salmo_102      GTGGCCTTCT TCGTGTGTAT CATTGGAACA CTGGGGGTGA CAGGGAACGC ATTGGTCATG TTCGCTTTCT GGAGT----- ---------- ---------- ----------
Salmo_103      GTGGCCTGCT TCGTTGTGGT GATCGGAGCG GTGGGGGTCG GTGGGAATGC CCTGGTGATG TACGCCTTCT TCAGT----- ---------- ---------- ----------
Salmo_104      GTGGCCTGTT TTGTCGTGGT GATTGGAGCA GTAGGGGTCG GTGGGAACGC CCTGGTGATG TACGCCTTCT TCAGT----- ---------- ---------- ----------
Stegas105      ---------- ---------- ---------- ---------- ---------- ---------- ---------- --AGG----- ---------- ---------- ----------
Terrap106      ATTGGAACTG TCATTCTGGT GGTGGGGATC ACCGGAACGC TGGGTAACTT CCTGGTCATT TATGCTTTCT GCAGG----- ---------- ---------- ----------
Xenopu107      GTTGGTGCTG TCATCCTTGC AGTGGGAATT ACAGGCATGC TGGGAAATTT TCTAGTCATC TATGCTTTCT GCAGA----- ---------- ---------- ----------


               ....|....| ....|....| ....|....| ....|....| ....|....| ....|....| ....|....| ....|....| ....|....| ....|....| ....|....|
                       120        130        140        150        160        170        180        190        200        210        220
Felis000       -aGcAgAgGa CtCaGgAcAc CtGcCaAcAt GtTcAtTaTc AaCcTcGcGg TcAgTgAcTt TtTcAtGtCc TtCaCcCaGg CcCcTgTcTt CtTcGcCaGc AgCcTcCaTa
Gallus001      -AACAAGAAG CTGAGGACAC CCCAGAACTT CTTTATAATG AATTTGGCTG TGAGTGACTT CCTGATGTCA GCTTCTCAGG CCCCCATATG CTTTATCAAC AGCTTGCACA
Gallus002      -AGTAGGACC CTTCAGAAAC CAGCCAATAT ATTCATCATC AATCTAGCTG TTAGTGACTT CCTTATGTCC ATTACACAGT CTCCAGTATT TTTCACCAAC AGTCTCCATA
Gadus003       -AACAAGAAG CTCCGTAATC TGCCCAACTA CTTCATCATG AACCAGGCGG CGAGCGACTT CCTCATGGCC TTCACCCAGT CGCCCTTCTT CTTCATCAAC TGTCTCAACA
Gadus_004      -AACAAGAAG CTCCGCACTC CTCCCAACTA TTTCATCATG AACTTGGCGG TTAGTGACTT CCTGATGGCC ATCACGCAGT CACCCATCTT CTTCATCAAC TCTCTCTTCA
Sminth005      -AGTCGCAGT CTTCGCACGC CCGCTAATAT GTTCATCATC AACCTCGCCA TCAGTGACTT CTTCATGTCC TTCACCCAAG CCCCTGTGTT TTTCGCCAGC AGCTTGTACG
Homo006  C     AGCAGAAGCC TCCGGACACC TGCCAACATG TTCATTATCA ACCTCGCGGT CAGCGACTTC CTCATGTCCT TCACCCAGGC CCCTGTCTTC TTCACCAGTA GCCTCTATAA
```

```
Mus007  CT   ACAGAGGCCT GCGGACACCA GCAAACATGT TCATCATCAA CCTCGCAGTC AGCGACTTCC TCATGTCAGT CACTCAGGCC CCGGTCTTCT TTGCCAGCAG CCTCTACAAG
Spalax008    -AGCAGAGGC CTTCGGACAC GAGCGAACAT GTTCACCGTC AACCTTGCAG TCAGTGACTT CCTCATGTCC TTCACTCAGG CCCCTGTCTT CTTTGCCAGC AGTCTCTATA
Monode009    -AGCCACAGC CTACGCACAC CAGCCAACAT GTTCATTATC AACCTTGCCA TCAGCGATTT TTTCATGTCC TTCACCCAAG CCCCAGTATT CTTTGCCAGC AGCATGTACA
Rattus010    -AATAGAGGC CTGCGGACAC CGGCAAACAT GCTCATCATC AACCTGGCAG TCAGCGACTT CCTTATGTCG TTCACTCAGG CCCCGGTCTT CTTTGCCAGC AGCCTCTACA
Podarc011    -AACAAGCGT CTGAGGACAC CGGCAAACTA TTTCATAATG AATTTAGCGG CAAGTGATTT TCTGATGTCT GCAACTCAAG CTCCAATCTG CTTTCTCAAC AGCATGCACA
Trache012    -AGTAGAAGC CTTCGAACTC CAGCCAACAT GTTCATCATC AATCTAGCTA TTAGCGACTT CCTAATGTCC ATCACACAGG CTCCAATTTT CTTCACTACC AGTCTCCACA
Xenopu013    -AACAAGAAG CTGAGGACGG CACCCAACTA CTTTATTATA AACTTAGCAA TCAGTGATTT TTTAATGTCA GCAACCCAGG CACCAGTTTG CTTTCTAAGC AGCTTGCACA
Danio014     -AGCCGGACC CTGAGGACCC CCGCCAACTT GTTTATAATA AACCTGGCCA TCACAGATTT CCTCATGTGT GCCACGCAGG CGCCCATCTT CTTTCACCACC AGCATGCATA
Danio_015    -AGCCGTACT CTTCGCACCG CTGGAAACAT GTTTATAGTG AACCTAGCTG TGGCTGATTT CTTAATGTCC GTCACCCAGT CTCCTGTGTT CTTTGCTGCA AGTTTACACA
Anolis016    -ATTCGGGGC CTTCGGACTC CTGCCAACAT GTTCGTTATC AATCTGGCTG TCAGTGATTT TCTAATGGCC ATCACTCAGT GTCCAGTTTT CTTCACCAAC AGCCTCAACA
Bos017  CT   GCAGAGGCCT CAGGACACCT GCCAACATGT TCATTATCAA CCTCGCAGTC AGCGACTTCC TCATGTCCTT CACTCAGGCC CCCGTCTTCT TCGCCAGCAG CCTCTATAAG
Callor018    -GACAAGAAA CTGAGGACGG CACCAAACTT CTTCATAACC AACCTGGCAG TCAGCGACTT CCTGATGTCC GCCTCCCAGT CTCCCGTCTT CTTCGTCAGC AGTTTAAACA
Canis019     -ACCAGAGGC CTCAGGACAC CCTCCAACAT GTTCATCATC AACCTCGCTG TCAGCGACTT CTTCATGTCC TTCACCCAGG CGCCCGTCTT CTTTGCCAGC AGCCTCCATA
Cynogl020    -AGTCGCAGC CTGCGGACCC CGGCCAACAT GTTCATCATC AACCTGGCCG TCACAGACCT GCTGATGTGC CTCACTCAGA CGCCCATCTT CTTCACCACC AGTCTGCACA
Dipodo021    -ACCAGAGGT CTGAGAACCC CCGCCAATAT GTTCATCATC AACCTTGCAG TCAGCGACTT CCTCATGTCC TTCACCCAGG CCCCTGTCTT CTTTGCCAGC AGCCTCCACA
Esox022  A   AGCAGGTCCC TGAAGACTCC AGCCAACATG TTCATCATTA ACCTGGCCAT CACTGATCTC CTGATGTGTC TCACCCAGGC ACCTATTTTC TTCACTACCT CTATGCACAA
Haliae023    -AGTAGGAGC CTTCAGACTC CAGCCAACAT ATTCATCATC AATCTAGCTA TTAGTGACTT CCTGATGTCC ATTACGCAGT CTCCAGTTTT TTTCACCAAC AGCCTCCACA
Macaca024    GAGCAGAGGC CTCCGGACAC CTGCCAACAT GTTCATTATC AACCTCGCCA TCAGCGACTT CCTCATGTCC TTCACCCAGG CCCCTGTCTT CTTCGCCAGC AGCCTCTATA
Nototh025    -AGCCGAAGT TTGCGGACAC CGTCCAACAT TTTCATCATT AACCTGGCAA TCACAGACTT GCTGATGTGT GTTACTCAGA CACCCACCTT CTTCATTAAC AGCATGCACA
Pygosc026    -AGTAGGAGC CTTCAGACTC CAGCCAACGT ATTCATCATC AATCTAGCTA TCAGTGACTT CCTGATGTCC ATTACGCAGT CTCCAGTTTT TTTCACCAAC AGCCTCCACA
Rutilu027    -AGCCGTACT CTTCGCACTG CAGGGAACAT GTTTGTGGTG AACCTGGCTG TGGCTGATTT CTTCATGTCC CTCACTCAGT CGCCCGTGTT CTTTGCAGCC AGCCTGCACA
Taenio028    ---------- ---------- ---------- ---------- ---------- ---------- ---------- ---------- ---------- ---------- ----------
Taenio029    ---------- ---------- ---------- ---------- ---------- ---------- ---------- ---------- ---------- ---------- ----------
Tarsiu030    GAGCAGAGGC CTCCGGACAC CTGCCAACAT GTTCGTCATC AACCTCGCGG TCAGTGACTT CCTCATGTCC TTCACCCAGG CCCCTGTCTT CTTCACCAGC AGTCTCTATA
Tinamu031    -AGTAGGAGC CTTCAAACAC CAGCCAACAT ATTTATCGTG AATCTGGCTA TCAGTGACTT TCTGATGTCC ATAACACAGT CTCCAATTTT TTTTACCAAT TGCCTCCACA
Trache032    ---------- ---------- ---------- ---------- ---------- ---------- ---------- ---------- ---------- ---------- ----------
Xenopu033    -AGTAGGAGT CTCCGATCTC CTGCAAATAT GTTCATCATC AACCTTGCTA TCACTGATTT CTTAATGTCG GTAACTCAGG CACCAGTATT TTTTGCAACT AGTCTGCATA
Bjar_O034    -AACAAGAAG CTGAGAACAC CTGCAAATTA TTTCATTATG AATTTAGCTG CAAGTGACTT TCTGATGTCT GCAACTCAAG CTCCAGTCTG CTTTTTCAAC AGTATGCACA
Cbic_O035    -AACAAGAGG CTGAGGACAC CTGCAAATTA TTTCATCATG AATTTAGCTG CAAGTGACTT TCTGATGTCT GCAACTCAAG CTCCAGTCTG CTTTTTCAAC AGTATGCATA
Cdur_O036    -AACAAGAGG CTGAGGACAC CTGCAAATTA TTTCATTATG AATTTAGCTG CAAGTGACTT TCTGATGTCT GCAACTCAAG CTCCAGTCTG CTTTTTCAAC AGTATGCACA
Dips_O037    -AACAAGAGG CTGAGGACAC CTGCAAATTA TTTCATCATG AATTTGGCTG CAAGTGACTT AATGATGTCT GCAACTCAAG CTCCAATCTG CTTTTTCAAC AGTATGCATA
Eceph_038    -AACAAGAGG CTGAGGACAC CTGCAAATTA TTTCATCATG AATTTRGCTG CAAGTGACTT TATGATGTCT GCAACTCAAG CTCCAGTCTG CTTTTTCAAC AGTATGAAAA
Eund_O039    -AACAAGAGG CTGAGGACAC CTGCAAATTA TTTCATCATG AATTTGGCTG CAAGTGACTT TATGATGTCT GCAACTCAAG CTCCAGTCTG CTTTTTCAAC AGTATGAAAA
Ery_OP040    -AACAAGAGG CTGAGGACAC CTGCAAATTA TTTCATCATG AATTTGGCTG CAAGTGACCT TATGATGTCT GCAACTCAAG CTCCAGTCTG CTTTTTCAAC AGTATGCATA
Hel_OP041    -AACAAGAGG CTGAGGACAC CTGCAAATTA TTTCATCATG AATTTGGCTG CAAGTGACTT TATGATGTCT GCAACTCAAG CTCCAATCTG CTTTTTCAAC AGTATGCAAA
Lmil_O042    -AACAAGAAG CTGAGGACAC CTGCAAATTA TTTCATCATG AATTTGGCTG CAAGTGACTT TATGATGTCT GCAACTCAAG CTCCAGTCTG CTTTTTCAAC AGTATGCATA
Mcor_O043    -AACAAGAGG CTGAGGACAC CTGCAAATTA TTTCATCATG AATTTAGCTG CAAGTGACTT TCTGATGTCT GCAACTCAAG CTCCAGTCTG CTTTTTCAAC AGTATGCATA
Mlem_O044    -AACAAGAAG CTGAGGACAC CTGCAAATTA TTTCATCATG AATTTAGCTG CAAGTGACTT TCTGATGTCT GCAACTCAAG CTCCAGTCTG CTTTTTCAAC AGTATGCATA
Oguib_045    -AATAAGAAG TTGAGGACAC CTGCAAATTA TTTCATCATG AATTTGGCTG CAAGTGACTT TATGATGTCT GCAACTCAAG CTCCAGTCTG CTTTTTCAAC AGTATGCGAA
Arizon046    ------GAGG CTGAGGACAC CTGCAAATTA TTTCATCATG AATTTAGCTG CAAGTGACTT TCTGATGTCT GCAACTCAAG CTCCAGTCTG CTTTTTCAAC AGTATGCATA
Cemoph047    ---------- ---------- ---------- -------ATG AATTTAGCTG CAAGTGACTT TCTGATGTCT GCTACTCAAG CTCCAATCTG CTTTTTCAAC AGTATGCATA
Hypsig048    ---------- ---------- ---------- ------CATG AATTTGGCTG CAAGTGACTT AATGATGTCT GCAACTCAAG CTCCAGTCTG CTTTTTCAAC AGTATGCATA
Lampro049    ---------- ---------- ---------- -------ATG AATTTAGCTG CAAGTGACTT TCTGATGTCT GCAACTCAAG CTCCAGTCTG CTTTTTCAAC AGTATGCATA
Pgutat050    -AACAAGAGG CTGAGGACAC CTGCAAATTA TTTCATCATG AATTTAGCTG CAAGTGACTT TCTGATGTCT GCTACTCAAG CTCCAGTCTG CTTTTTCAAC AGTATGCATA
Mastic051    ---------- ---------- ---------- ---------- ---------- ---------- ---------- ---------- ---------- ---------- ----------
Ppat_O052    -AACAAGAGG CTGAGGACAC CTGCAAATTA TTTCATCATG AATTTGGCTG CAAGTGACTT TATGATGTCT GCAACTCAAG CTCCAGTCTG CTTTTTCAAC AGTATGCATA
Smik_O053    -AACAAGAGG CTGAGGACAC CTGCAAATTA TTTCATCATG AATTTGGCTG CAAGTGACTT AATGATGTCT GCAACTCAAG CTCCAATCTG CTTTTTCAAC AGTATGCATA
Snew_O054    -AACAAGAGG CTGAGGACAC CTGCAAATTA TTTCATCATG AATTTGGCTG CAAGTGACTT AATGATGTCT GCAACTCAAG CTCCAATCTG CTTTTTCAAC AGTATGCATA
Tae_OP055    -AACAAGAGG CTGAGGACAC CTGCAAATTA TTTCATCATG AATTTGGCTG CAAGTGACTT TATGATGTCT GCAACTCAAG CTCCAGTCTG CTTTTTCAAC AGTATGAAAA
Tham_O056    -AACAAGAGG CTGAGGACAC CTGCAAATTA TTTCATCATG AATTTGGCTG CAAGTGACTT AATGATGTCT GCAACTCAAG CTCCAGTCTG CTTTTTCAAC AGCATGCAAA
Tdor_O057    -AACAAGAGG CTGAGGACAC CTGCAAATTA TTTCATCATG AATTTGGCTG CAAGTGACTT AATGATGTCT GCAACTCAAG CTCCAGTCTG CTTTTTCAAC AGTATGCAAA
Anolis058    ---------- ---------- ---------- ---------- ---------- ---------- ---------- ---------- --TCGGCTGT ATTGGAATTA CAGGAAACCT
Pogona059    -AACAAGAGG CTGAGGACTC CACCGAACTA TTTCATAATG AATTTAGCAG CAAGTGATTT CTTGATGTCT GCAACTCAGG CTCCCATATG CTTTCTCAAC AGCATGCACA
Protob060    -AACAAGAGG CTGAGGACAC CTGCAAATTA TTTCATTATG AATTTAGCTG CAAGTGACTT TCTGATGTCT GCAACTCAAG CTCCAGTCTG CTTTTTCAAC AGTATGCACA
Thamno061    ---------- ---------- ---------- -------ATG AATTTAGCTG CAAGTGACTT TCTGATGTCT GCAACACAAG CTCCAGTCTG CTTTTTCAAC AGTCTGTATA
Alliga062    -AACAAGAAG TTGAGGACAC CACAAAACTA CTTCATAATG AATTTGGCTA CAAGTGACTT TCTGATGGCT GCTTCTCAGG CTCCCGTTTC CTTTATCAAC AGCTTGCACA
Athene063    -AGTAGGAGC CTTCAGACTC CCGCCAACAT ATTCATCATC AATCTAGCCA TTAGTGACTT CCTGATGTCC GTTACGCAGT CTCCAGTTTT TTTCACCAAC AGCCTCCACA
Callor064    -AGCAGAAGT CTCCGTACGC CAGCCAACAC CTTCATCATC AACCTTGCAG CCACCGATTT CCTCATGTCG GTCACCCAGT CCCCAATCTT CTTCATCACC AGTATCCACA
Chelon065    -AGTAAGAGC CTTCGAACTC CAGCCAACAT GTTCATCATC AATCTAGCTA TTAGCGACTT CCTAATGTCC ATCACACAGG CTCCAATTTT CTTCACTACC AGTCTCCACA
Chelon066    -AACAAGAAA CTGAGGACAG CGCCAAACTA CTTTATAATG AATTTGGCTG TGAGTGACTT CCTGATGTCT GCTTCTCAGG CCCCCATTTG CTTTATCAAC AGTTTGCACA
Chryse067    -AGTAGGAGC CTTCGAACTC CAGCCAACAT GTTCATCATC AATCTAGCTA TTAGCGACTT CCTAATGTCC ATCACACAGG CTCCAATTTT CTTCACTACC AGTCTCCACA
Danio_068    -AGCCGTACT CTTCGCACCG CTGGAAACAT GTTTATAGTG AACCTAGCTG TGGCTGATTT CTTAATGTCC GTCACCCAGT CTCCTGTGTT CTTTGCAGCC AGTTTACACA
Danio_069    -AACAAGAAG CTGCGGACGC CTCCAAACTA CTTCATCATG AATCTGGCAG TGAGTGACTT CCTCATGGCC ATCACTCAGT CACCCATCTT CTTCATCAAC TGCATGTACA
Danio_070    -AGCCGGAGT TTGAGAACAC CTGCCAACAT GTTCATCATC AATCTTGCTG TCACTGATTT CCTGATGTGT GTCACTCAAA CACCAATCTT CTTCACCACC AGTTTGCACA
Danio_071    -AACAAAAAG TTGCGGAGCC TGCCTAATTA TTTCATCATG AACCTGGCGG TTAGTGATTT CCTCATGGCC ATCACACAGT CTCCAATCTT CTTCATCAAC TGCCTGTATA
Egrett072    -AGTAGGAGC CTTCAGACTC CAGCCAACGT ATTCATCATC AATCTAGCTA TTAGTGACTT CCTGATGTCC ATTACACAGT CTCCAGTTTT TTTCACTAAC AGCCTCCACA
Empido073    -ACTAGGAGC CTTCAGACTC CAGCCAACAT GCTCATCATC AATCTGGCTA TAAGTGACCT CCTGATGTCC ATTACACAGT CTCCAGTTTT TTTCGTCAAC AGCCTCCACA
Eptatr074    -AGCAAACCC CTGCGGACTC CAACCAACAT GCTGATCGTT AACCTGGCTT TCAGTGACTT CATGATGTCC ATTACTCAAG CGCCAGCGTT TTTCGTTGCC AGTCTGCACA
Eptesi075    -AGCAGAGGT CTCAGGACGC CTGCCAACAT GTTCATCATC AACCTTGCGG TCAGCGACTT CCTCATGTGC TTCACGCAGG CCCCCGTGGT CTTCGCCAGC AGCCTCTACA
Erpeto076    -TGTCGCAGT CTGCGCACAC CAGCCAACAT GTTTATCATC AACCTGGCTA TCACTGACTT TCTAATGTCT GTAACACAGG CCCCCATATT CTTCACAACA AGCCTATATA
Falco_077    -AGTAGGAGC CTTCAGACTC CAGCCAACAT ATTCATCATC AATCTAGCTA TTAGTGACTT CCTGATGTCC ATTACGCAGT CTCCAGTTTT TTTCACCAAT AGTCTCCACA
Gekko_078    -AACAAGAAG CTGAGGAGAC CACCAAACTA TTTTATAATG AATTTAGCAG CAAGTGATTT CCTGATGTCT GCAACTCAGG CCCCCATCTG CTTTCTCAAC AGCATGCACA
Hippog080    -AGTCGCAGC CTGAGGACAC CGGCCAATAT GTTCATCATT AACCTGGCGA TCACAGACCT GCTGATGTGC ATCACTCAGA CGCCCATCTT CTTCACCACC AGCATGCACA
Hippog081    -AGCCGGAGC TTGCGGACAC CGTCCAACAT CTTTATCATT AACCTGGCAG TCACAGACCT CCTCATGTGT TTCACCCAGA CGCCCATCTT CTTCCTCACC AGCATGCACA
Hippog082    -AACAAGAAT CTGCGGACGC CACCCAACTT TTTCATCATG AATCTGGCCG TCAGTGACTT CCTCATGGCG ATCACACAGT CGCCCATCTT CTTCGTTAAC TCCCTGTACA
Hippog083    -AACAAGAAG CTTCGTAACC TGCCCAACTA CTTCATCATG AACTTGGCGA TCAGCGACTT CCTCATGGCC TTCACACAGT CGCCCATCTT CTTCATCAAC TGTCTCTACA
Ictalu084    -AGTCGAAGC CTAAGAACAC CAGCCAACAT GTTCATTATT AATCTGGCCA TAACTGATTT CCTGATGTGT GTAACTCAAA CACCCACCTT CTTCATTACC AGCATGCACA
Ictalu085    -AGCCGCCCA CTACGTACAC CAGGGAACAT GTTGGTGGTC AACCTGGCTG TGTCTGACTT CTTCATGTCA CTCACTCAGC CGCCAGTGTT CTTTGTGGCC AGCCTTCACC
Larimi086    -AGTCGCAGT CTGCGGACGC CGGCCAACAT GTTCATCATT AACCTGGCCA TCACAGACCT GCTGATGTGT ATCACCCAAA CGCCCACGTT CTTCATCACC AGCATGAACA
Lethen087    -AGCAAGAGT TTGCGCTCGC CGGCCAACAT TTTTATCATC AACCTGGCCT TCGCGACTT CTTCATGTCC TTCACACAGA CACCCATTTT CTTCGTCACG AGTCTTCACA
Mastac088    -AGTCGCAGC CTGCGGACGC CGTCCAACAT GTTCATCATT AACCTGGCTA TCACTGACTT GCTGATGTGT GTCACCCAAA CACCCACCTT CTTCGTCACC AGCATGCACA
Meleag089    -AACAAGAAG TTGGGGACAC CCCAGAACTT CTTTATAATG AACTTGGCTG TGAGTGACTT CTTGATGTCA GCTTCTCAGG CCCCCATATG CTTTGTCAAC AGCTTGCACA
Meleag090    -AGTAGGACC CTTCAGAAAC CAGCCAATAT ATTCATAATC AATCTAGCTG TTAGTGACTT CCTTATGTCC ATCACGCAGT CTCCAGTGTT TTTCACCAAC AGTCTCCATA
Notech091    -AACAAGAGG CTGAGGACAC CTGCAAATTA TTTCATCATG AATTTAGCTG CAAGTGACTT TCTGATGTCT GCAACTCAAG CTCCAGTCTG CTTTTTCAAC AGTATGCATA
Opisth092    -AGTAGGAGC CTTCGGACTC CAGCCAACAT ATTCATCATC AATCTAGCTA TTAGTGACTT CCTGATGTCC ATTACGCAGT CTCCAATTTT TTTCACCAAC AGCCTCCACA
Pelodi093    -AGTAAGAGT CTTCGAACTC CAGCCAACAT GTTCATCATC AATCTAGCTA TTAGCGACTT CTTAATGTCC ATCACACAGG CTCCAATTTT CTTTGCTACC AGTCTCCACA
Phyllo094    -AGCAGAGGC CTCAGGACAC CTGCCAACAT GTTCATTATC AACCTTGCAA TCAGCGACTT CCTCATGTCC TTCACCCAGG CCCCTGTGT ATTTGCCAGC AGCCTCTATA
Physet095    GAGCAGAGGT CTCAGGACAC CTGCCAACAT GTTCATCATC AACCTCGCAG TCAGCGACTT CCTCATGTCC TTCACCCAGG CCCCCGTCTT CTTCGCCAGC AGCGTCTATA
Pipra_096    -ACTAGGAGC CTTCGGACTC CAGCCAACAT GCTCATCATC AATCTGGCTA TTAGTGACCT CCTGATGTCC ATCACACAGT CTCCAGTTTT TTTCACCAAC AGCCTCCACA
Podarc097    -AACAAGCGG CTGAGGACAC CGGCAAACTA TTTCATAATG AATTTAGCGG CAAGTGATTT TCTGATGTCT GCAACTCAAG CTCCAATCTG CTTTCTCAAC AGCATGCACA
Podarc098    -AGCAGAGGC CTTCGGACTC CCGCCAACAT GTTCATTATC AACCTGGCTG TTAGTGATTT CCTAATGGCC GTCACCCAGT GTCCAGTTTT CTTCACCAGC AGTCTCAACA
Pseudo099    -AACAAGAGG CTGAGGACAC CTGCAAATTA TTTCATCATG AATTTAGCTG CAAGTGACTT TCTGATGTCT GCAACTCAAG CTCCAGTCTG CTTTTTCAAC AGTATGCATA
Python100    -AACAAGAGA CTGAGGACAC CTCCAAATTA TTTCATCATG AATTTAGCCG TGAGTGATTT TCTGATGTGT GCAACTCAAG CTCCAGTCTG CTTTTTCAAC AGTATACATA
Salmo_101    -AGCAGGTCA CTGAGGACTC CGGCCAACAT GTTCATCATT AACCTGGCCA TCACTGACCT CCTGATGTGC CTCACACAGG CACCCATCTT CTTCACCACC TCCATGCATA
Salmo_102    -AATAAGAAA CTCCGCAATC TGCCCAACTA CTTCATCATG AACCAGGCGG TGAGCGACTT CCTCATGGCT TTCACACAGT CGCCCATCTT CTTCATTAAC TGTCTCTATA
Salmo_103    -AACAAGAAG CTCCGGACTC CTCCCAACTA TTTCATCATG AATCTGGCAG TGAGTGATTT CCTCATGGCT ATCACACAGT CCCCTATCTT CTTTGTCAAC TGTCTCTACA
Salmo_104    -AACAAGAAG CTTCGGACTC CTCCCAACTA TTTCATCATG AACCTGGCAG TGAGTGACTT CCTCATGGCC GTCACACAGT CCCCCATCTT CTTTGTCAAC TGTCTCTACA
Stegas105    -AGTCGCAGC CTGCGGACGC CGGCCAACAT GTTCATCATC AACCTGGCCA TCACAGACCT GCTGATGTGC GTCACCCAGT CGCCCATCTT CTTCACCACC AGCATGCACA
Terrap106    -AGTAGGAGC CTTCGAACTC CAGCCAACAT GTTCATCATC AATCTAGCTA TTAGCGACTT CCTAATGTCC ATCACACAGG CTCCAATTTT CTTCACTACC AGTCTCCACA
Xenopu107    -AGTAGGAGC CTTCGATCTC CTGCAAATAT GTTCATCATC AACCTAGCTA TCACTGATTT CTTAATGTCT GTAACTCAGG CACCAGTATT TTTTGCAACT AGTCTGCATA


             ....|....| ....|....| ....|....| ....|....| ....|....| ....|....| ....|....| ....|....| ....|....| ....|....| ....|....|
                    230        240        250        260        270        280        290        300        310        320        330
Felis000     AgCgGtGgCt CtTcGgGgAg GcAgGcTgCg AgTtCtAtGc CtTcTgTgGg GcTcTcTtTg GcAtCaCcTc CaTgAtCaCc CtGaTgGcCa TtGcTcTgGa CcGcTaCcTg
Gallus001    GAGAGTGGAT CCTTGGAGAT ATAGGTTGTG ACTTGTATGC TTTCTGCGGG GCGCTCTTTG GAATAACCTC GATGATGACT CTGTTAGCTA TTTCTGTTGA CCGCTATTTG
Gallus002    AACGTTGGAT TTTTGGTGAG AAAGGCTGTG AGCTGTATGC CTTCTGCGGA GCTCTTTTTG GCATTACATC CATGATCACT TTGATGGTGA TTGCCTTGGA CAGATACTTT
Gadus003     GGGAGTGGAT CTTCGGAGAG CTCGGCTGTA AGCTGTATGC GTTCCTGGGG GCCCTATTTG GCATCACGTC CATGATCAAC CTGCTGGCCA TCTCCCTGGA CCGCTACATG
Gadus_004    AAGAGTGGAT TTTTGGCGAG ACCGGCTGTA GGATGTATGC CTTCTGTGGG GCGTTGTTCG GTATCACCTC AATGATCAAC CTGCTGGCCA TCTCCCTGGA CCGCTACATC
Sminth005    AACGCTGGAT CTTTGGAGAG AAAGGATGTG AATTTTATGC CTTCTGTGGG GCTCTCTTTG GCATTACCTC CATGATAACG CTGATGGTGA TAGCCCTGGA CCGGTACTTT
Homo006  C   GCAGTGGCTC TTTGGGGAGA CAGGCTGCGA GTTCTATGCC TTCTGTGGGG CTCTCTTTGG CATTTCCTCC ATGATCACCC TGACGGCCAT CGCCCTGGAC CGCTACCTGG
Mus007  CT   AAGTGGCTCT TTGGGGAGAC AGGTTGCGAG TTCTATGCCT TCTGCGGGGC TGTCTTTGGC ATCACTTCCA TGATCACCCT GACAGCCATA GCCATGGACC GCTATCTGGT
Spalax008    AGCGGTGGCT CTTTGGGGAG GCAGGCTGCG AGTTCTATGC CTTCTGTGGG GCTGTCTCTG GCATCACCTC CATGACCACC CTGACTGCCA TAGCCCTGGA CCGCTACCTG
Monode009    AACGCTGGAT CTTTGGAGAG AAAGCGTGTG AATTTTATGC CTTCTGTGGG GCTCTCTTCG GCATCACCTC CATGATAACG CTGATGGCAA TTGCCCTGGA CCGGTACTTT
Rattus010    AGAAGTGGCT CTTCGGGGAG ACAGGTTGCA AGTTCTATGC CTTCTGTGGG GCTGTCTTTG GCATCGTTTC CATGATCACC CTGACAGCCA TAGCCATGGA CCGCTATCTG
Podarc011    CAGAATGGAT ACTCGGAGAC ATAGGTTGTA ACTTTTATGT ATTTTGTGGG GCACTCTTTG GAATAACTTC AATGATGACT TTATTAGCTA TTTCAGTTGA TCGCTACTGT
Trache012    AGCACTGGAT TTTTGGCAAG AAAGGTTGTG AGCTGTATGC CTTCTGCGGA GCCCTTTTTG GCATTACATC CATGATCACG TTGATGGCAA TCGCACTGGA CAGATATTTT
Xenopu013    GGGAGTGGAT ACTTGGAGAT ATAGGCTGTA ATGTGTATGC GTTTTGTGGG GCACTGTTTG GAATAACCTC TATGATGACA TTATTGGCTA TATCAATCAA TCGATATATA
Danio014     AAAGATGGAT CTTTGGAGAG AAAGGTTGTG AGTTGTATGC CTTCTGTGGG GCTTTATTTG GCATCTGCTC CATGATCACG CTCATGGTTA TTGCTGTGGA TCGATACTTT
Danio_015    GGCGATGGGT ATTTGGCGAG CGTCCTTGCG AGCTCTACGC CTTCTGCGGT GCCCTCTTTG GAATCTGCTC CATGATGACT TTGACCGCCA TCGCTGCTGA TCGTTGTCTC
Anolis016    AACGGTGGAT ATTTGGTGCA AAAGGCTGTG AGCTGTACGC ATTCTGTGGA GCTCTCTTTG GCATTGCTTC CATGATCACC TTGACAGTGA TAGCCCTGGA CAGGTATTTT
Bos017  CT   CAGTGGCTCT TTGGAGAAGC AGGCTGTGAA TTCTATGCCT TCTGTGGGGC TCTCTTTGGC ATCACCTCCA TGATTACCCT GACGGCCATC GCCCTGGACC GCTACCTGGT
Callor018    AGCGCTGGAT ATTTGGCAAT ATCGGTTGTA AACTATATGC CTTCTGCGGA GCACTGTTTG GCATTACATC TATGATTACT TTAATGGCAA TCTCTATTGA TCGCTATTGG
```

```
Canis019     AGCGGTGGCT CTTTGGGGAG GCAGGCTGCG AGTTCTACGC CTTCTGCGGG GCTCTGTTCG GCATTACCTC TATGATCACC CTGACGGCCA TCGCTCTGGA CCGCTACCTG
Cynogl020    AGAGGTGGAT CTTTGGAGAG AAAGCCTGTG AGGTATATGC TTTCTGTGGC GCCCTCTTTG GGATCTGCTC CATGATCACA CTGACTGTGA TTGCGGTCGA CCGCTACGTT
Dipodo021    AGCAGTGGCT CTTTGGAGAG GCAGGCTGCG AATTCTATGC CTTCTGTGGA GCTCTCTTTG GCATCACCTC CATGATCACC CTGATGGCCA TTGCCCTGGA CCGGTATCTG
Esox022  A   AAGATGGATC TTTGGAGAGA AAGCTTGTGA GATGTATGCA TTCTGCGGGG CCCTGTTCGG CATCTGCTCT ATGATCACTA TGACGGTGAT AGCAGTGGAC CGCTACTTCG
Haliae023    AACGCTGGAT TTTTGGTGAG AAAGGCTGTG AGCTGTATGC CTTCTGCGGA GCTCTGTTTG GCATTACGTC TATGATCACT TTGATGGTGA TTGCCTTGGA CAGATATTTT
Macaca024    AGCACTGGCT CTTTGGGGAG ACAGGCTGCG AGTTCTATGC CTTCTGTGGG GCTCTCTTTG GCATCTCCTC CATGATCACC CTGACGGCCA TCGCCCTGGA CCGCTACCTG
Nototh025    AGCGATGGAT CTTTGGAAAG AAAGGTTGTG AAATATACGC CTTTTGCGGA GCGCTGTTTG GTATCTGCAG TATGATAACA CTAATGGTGA TTGCGGTGGA CCGGTACGTA
Pygosc026    AACGCTGGAT TTTTGGTGAG AAAGGCTGTG AGCTGTATGC CTTCTGCGGA GCTCTTTTTG GCATTACATC TATGATCACT CTGATGGTGA TTGCCTTGGA CAGATATTTT
Rutilu027    GGCGCTGGAT CTTTGGTGAG CGCATTTGTG AGCTCTACGC CTTTTGTGGT GCCCTTTTTG GGATCTGTTC CATGATGACT TTGACTGCCA TTGCTGCTGA TCGTTGCCTA
Taenio028    ---------- ---------- ---------- ---------- ---------- ---------- ---------- ---------- ---------- ---------- ----------
Taenio029    ---------- ---------- ---------- ---------- ---------- ---------- ---------- ---------- ---------- ---------- ----------
Tarsiu030    AGCAGTGGCT CTTTGGGGAG ACAGGCTGTG AGTTCTATGC CTTCTGCGGG GCTCTCTTCG GCATCTCCTC CATGATCACC CTGACGGCCA TCGCCTTGGA TCGCTACCTG
Tinamu031    AACGCTGGAT TTTTGGTGAG AAAGGCTGTG AACTGTATGC CTTCTGCGGA GCTCTTTTTG GCATTACATC AATGATCACT TTGACGGTGA TTGCCCTGGA CAGATATTTT
Trache032    ---------- ---------- ---------- ---------- ---------- --------TG GAATAACCTC GATGATGACT TTATTAGCTA TTTCCATTGA TCGATACCTT
Xenopu033    AAAGATGGAT CTTTGGAGAA AAAGGTTGCG AGTTATACGC TTTTTGCGGA GCCCTTTTTG GAATTACTTC CATGATCACA CTGATGGTGA TTGCTGTGGA TAGATATTTT
Bjar_O034    AGGAGTGGGT ACTTGGAGAC ACAGGTTGTA ACTTTTATGC TTTTTGTGGA GCACTKTTTG GAATAACCTC AATGATGACC TTGTTGGCTA TTTCGGTGGA TCGCTACTGT
Cbic_O035    AGGAGTGGGT ACTTGGAGAC ACAGGTTGTA ACTTCTATGC TTTTTGTGGA GCACTCTTTG GAATAACCTC AATGATGACC TTGTTAGCTA TTTCGGTCGA TCGCTACTGT
Cdur_O036    AGGAGTGGGT ACTTGGAGAC ACAGGTTGTA ACTTTTATGC TTTTTGTGGA GCACTGTTTG GAATAACCTC AATGATGACC TTGTTGGCTA TTTCGGTGGA TCGCTACTGT
Dips_O037    AGGAGTGGGT TCTTGGAGAC ATAGGTTGTA ACTTCTACGC TTTTTGTGGA GCACTCTTTG GAATAACCTC AATGATGACC TTGTTAGCTA TTTCAGTGGA TCGCTACTGT
Eceph_038    CAGAGTGGGT ACTTGGAGAC ATAGGTTGTA ACTTCTATGC TTTTTGTGGA GCACTCTTTG GAATAACCTC AATGATGACC TTGTTAGCTA TTTCGGTGGA TCGCTACTGT
Eund_O039    CAGAGTGGGT ACTTGGAGAC ATAGGTTGTA ACTTCTATGC TTTTTGTGGA GCACTCTTTG GAATAACCTC AATGATGACC TTGTTAGCTA TTTCGGTGGA TCGCTACTGT
Ery_OP040    CGGAGTGGGT ACTTGGAGAC ATAGGTTGTA ACTTCTATGC TTTTTGTGGA GCACTCTTTG GAATAACCTC AATGATGACC TTGTTAGCTA TTTCGGTGGA TCGCTACTGT
Hel_OP041    CAGAGTGGGT ACTTGGAGAC ATAGGTTGTA ACTTCTATGC TTTTTGTGGA GCACTCTTTG GAATAACCTC AATGATGACC TTGTTAGCTA TTTCAGTGGA TCGCTACTGT
Lmil_O042    CGGAGTGGGT ACTTGGAGAC ATAGGTTGTA ACTTCTATGC TTTTTGTGGA GCACTCTTTG GAATAACCTC AATGATGACC TTGTTAGCAA TTTCGGTGGA TCGCTACTGT
Mcor_O043    AGGAGTGGGT ACTTGGAGAC ACAGGTTGTA GCTTCTATGC TTTTTGTGGA GCTCTCTTCG GAATAACCTC AATGATGACC TTGTTAGCTA TTTCGGTGGA TCGCTACTGT
Mlem_O044    AGGAGTGGGT ACTTGGAGAC ACAGGTTGTA GCTTCTATGC TTTTTGTGGA GCTCTCTTCG GAATAACCTC AATGATGACC TTGTTAGCTA TTTCGGTGGA TCGCTACTGT
Oguib_045    CAGAGTGGGT ACTTGGAGAC ATAGGTTGTA ACTTCTATGC TTTTTGTGGA GCACTCTTTG GAATAACCTC AATGATGACC TTGTTAGCTA TTTCGGTGGA TCGCTACTGT
Arizon046    AGGAGTGGGT ACTTGGAGAC ACAGGTTGTA ACTTCTATGC TTTTTGTGGA GCCCTCTTTG GAATAACCCC AATGATGACC TTGTTAGCTA TTTCGGTGGA TCGCTACTGT
Cemoph047    AGGAGTGGGT ACTTGGAGAC ATAGGTTGTA ACTTCTATGC TTTTTGTGGA GCCCTCTTTG GAATAACTTC AATGATGACC TTGTTAGCTA TTTCGGTGGA TCGCTACTGT
Hypsig048    AGGAATGGGT ACTTGGAGAC ATAGGTTGTA ACTTCTATGC TTTTTGTGGA GCACTCTTTG GAATAACCTC AATGATGACC TTGTTAGCTA TTTCGGTGGA TCGCTACTGT
Lampro049    AGGAGTGGGT ACTTGGAGAC ATAGGTTGTA ACTTCTATGC TTTTTGTGGA GCACTCTTTG GAATAACCTC AATGATGACC TTGTTAGCTA TTTCGGTGGA TCGCTACTGT
Pgutat050    AGGAGTGGGT ACTTGGAGAC ATAGGTTGTA ACTTCTATGC TTTTTGTGGA GCACTCTTTG GAATAACCTC AATGATGACC TTGTTAGCTA TTTCGGTGGA TCGCTACTGT
Mastic051    ---------- ---------- ---------- ---------- ---------- ---------- ---------- ---------- ---------- ---------- ----------
Ppat_O052    CAGAGTGGGT ACTTGGAGAC ATAGGTTGTA ACTTCTATGC CTTTTGTGGA GCACTCTTTG GAATAACCTC AATGATGACC TTGTTAGCTA TTTCGGTGGA TCGCTACTGT
Smik_O053    AGGAGTGGAT TCTTGGAGAC ATAGGTTGTA ACTTCTACGC TTTTTGTGGA GCACTCTTTG GAATAACCTC AATGATGACC TTGTTAGCTA TTTCGGTGGA TCGCTACTGT
Snew_O054    AGGAGTGGGT TCTTGGAGAC ATAGGTTGTA ACTTCTACGC TTTTTGTGGA GCACTCTTTG GAATAACCTC AATGATGACC TTGTTAGCTA TTTCGGTGGA TCGCTACTGT
Tae_OP055    CAGAGTGGGT ACTTGGAGAC ATAGGTTGTA ACTTCTATGC TTTTTGTGGA GCACTCTTTG GAATAACCTC AATGATGACC TTGTTAGCTA TTTCGGTGGA TCGCTACTGT
Tham_O056    CGGAGTGGGT ACTTGGAGAC ATAGGTTGTA ACTTTTATGC TTTTTGTGGA GCACTCTTTG GAATAACCTC AATGATGACC TTGTTAGCTA TTTCCGTGGA TCGCTACTGT
Tdor_O057    CGGAGTGGGT ACTTGGAGAC ATAGGTTGTA ACTTCTATGC TTTTTGTGGA GCACTCTTTG GAATAACCTC AATGATGACC TTGTTAGCTA TTTCCGTGGA TCGCTACTGT
Anolis058    TCTCGTCCTT TATGCATTTT ACAGGTTGTA ACTTGTATGC TTTTTGTGGG GCGCTCTTCG GAATAACCTC AATGATTACG TTGTTGGCTA TTTCGGTTGA TCGTTACTGT
Pogona059    GAGAATGGGT CCTTGGAGAT ACAGGTTGCA ACCTGTATGC TTTCTGCGGG GCACTCTTTG GAATAACCTC AATGATGACC TTATTAGCTA TTTCAGTTGA TCGCTACTGT
Protob060    AGGAGTGGGT ACTTGGAGAC ACAGGTTGTA ACTTTTATGC TTTTTGTGGA GCACTGTTTG GAATAACCTC AATGATGACC TTGTTGGCTA TTTCGGTGGA TCGCTACTGT
Thamno061    CGGAGTGGGT TCTTGGAGAT GCAGGTTGTA ACTTCTATGC TTTTTGTGGA GCACTCTTTG GAATAACCTC AATGATGACC TTGTTAGCTA TTTCGGTGGA TCGCTACTGT
Alliga062    AAGAATGGAT ACTTGGAGAC ATAGGTTGTG AGCTGTATGC TTTTTGTGGA GCACTCTTTG GAATCACTTC AATGATGACT TTAGTAGCTA TTTCTGTTGA TCGCTACCTT
Athene063    AACGCTGGAT TTTTGGTGAG AAAGGCTGTG AGCTGTATGC CTTCTGCGGA GCTCTTTTTG GCATTACATC TATGATCACT TTGATGGTGA TTGCCTTGGA CAGATATTTT
Callor064    AGCGCTGGAT CTTCGGAGAA AAAGGCTGTG AGCTCTATGC TTTCTGCGGC GCTCTCTTTG GCATCACCTC GATGATCACG CTGATGGTGA TCGCCTTGGA CAGGTACTTT
Chelon065    AGCACTGGAT TTTTGGCAAG AAAGGCTGTG AACTGTATGC CTTCTGTGGA GCCCTTTTTG GCATTACATC CATGATCACA TTGATGGCAA TCGCACTGGA CAGATATTTT
Chelon066    AAGAGTGGAT ACTTGGCAAA ATAGGTTGTG ACCTGTATGC TTTTTGCGGG GCGCTCTTTG GAATAACCTC AATGATGACT TTATTAGCTA TTTCCATTGA TCGATACCTT
Chryse067    AGCACTGGAT TTTTGGCAAG AAAGGTTGTG AGCTGTATGC CTTCTGCGGA GCCCTTTTTG GCATTACATC CATGATCACA TTGATGGCAA TCGCACTGGA CAGATATTTT
Danio_068    GGCGCTGGGT ATTTGGCGAG CGCCCTTGCG AGCTCTACGC CTTCTGCGGT GCCCTCTTTG GAATCTGCTC CATGATGACT TTGACCGCTA TCGCTGCTGA TCGTTGTCTC
Danio_069    AGGAGTGGAT GTTCGGGGAA ATGGGCTGTA AGATGTACGC CTTCTGCGGA GCTCTGTTTG GGATCACATC CATGATCAAC CTTCTGGCCA TTTCTATCGA CCGCTACATC
Danio_070    AGCGATGGAT CTTTGGAGAA AAAGGCTGTG AGTTATATGC ATTCTGCGGT GCTCTGTTTG GTATCTGCTC GATGATCACA CTCATGATTA TTGCAGTAGA TCGATACTTC
Danio_071    AGGAGTGGAT GTTTGGAGAA CTGGGATGTA AAATCTATGC ATTCTGTGGT GCGCTGTTCG GCATCACCTC GATGATAAAC CTGTTGGCCA TTTCTATTGA TCGCTACCTG
Egrett072    AACGTTGGAT TTTTGGTGAG AAAGGCTGTG AGCTGTATGC CTTCTGCGGA GCTCTGTTTG GCATTACATC TATGATCACT TTGATGGTGA TTGCCTTGGA CAGATATTTT
Empido073    AACGCTGGAT TTTTGGTGAG AAAGGCTGTG AGCTGTATGC CTTCTGTGGA GCTCTCTTTG GCATTACATC TATGATCACT TTGATGGTGA TTGCCTTGGA CAGATATTTT
Eptatr074    AGCGCTGGAT TTTCGGCGAG AAAGGTTGCG AGCTCTACGG GTTCTGTGGG GCTGTTTTCG GAATATCTTC GATGATTACA CTCACCGCCA TAAGCATTGA CCGCTACTAT
Eptesi075    AGCGCTGGCT CTTCGGGGAG GCAGGCTGCG AGTTCTATGC CTTCTGTGGG GCTCTCTTCG GCATCACCTC CATGATCACC CTCACGGCCA TTGCCCTGGA CCGCTACCTG
Erpeto076    AGAGATGGAT ATTTGGTGAG AAAGGTTGTG AGCTTTATGC CTTCTGTGGT GCCCTCTTTG GGATCTGCTC AATGATTACT CTGATGGTGA TAGCAGTAGA CCGTTACTTT
Falco_077    AACGCTGGAT TTTTGGTGAG AAAGGCTGTG AGCTGTATGC CTTCTGCGGA GCTCTTTTTG GCATTACATC TATGACCACT TTGATGGTGA TTGCCTTGGA CAGATATTTT
Gekko_078    GGGAATGGAT ACTTGGAGAC ATAGGTTGCA ACTTGTATGC TTTTTGCGGG GCGCTGTTTG GAATAACTTC CATGATGACT TTGTTAGCCA TTTCAGTTGA TCGCTACTGT
Hippog080    AGAGGTGGAT CTTCGGAGAG AAAGGCTGCG AGCTGTACGC CTTTTGTGGC GCCCTCTTTG GAATCTGCTC CATGATCACG CTGACGGTGA TCGCCATCGA CCGCTACTTC
Hippog081    AGCGATGGAT CTTTGGGAAG AAAGGTTGTG AGTTGTATGC CTTCTGCGGA GCGCTGTTTG GTATCTGCAG TATGATGACT CTGATGGTGA TTGCGCTGGA CCGGTATGTG
Hippog082    AAGGCTGGAT TTTTGGTGAA ACAGGGTGTA AAATGTATGC CTTCTGTGGA GCTTTATTTG GAATCACCTC CATGATAAAC CTCCTGGCAA TCTCCATCGA CCGCTACATC
Hippog083    AGGAATGGGT GTTCGGAGAG ATGGGCTGTA AGTTGTACGC ATTCTGTGGC GCCCTGTTCG GCATTGCCTC CATGATCAAC CTACTGGCCA TCTCCATCGA CCGCTACGTG
Ictalu084    GGAGATGGAT CTTTGGAGAG AAAGGCTGTG AGATGTATGC ATTTTGTGGT GCTCTCTTCG GGATGTGCTC TATGATCACA CTAATGATCA TTGCTGTGGA TAGATATATT
Ictalu085    GGCGTTGGGT GTTCAGCGAG CTTGCGTGTG AACTGTATGC TTTCTGTGGT GCCCTCTTTG GGATCTGCTC CATGATGACT CTAACAGCTA TAGCTGTCGA CCGCTGCCTT
Larimi086    GGAGGTGGAT CTTTGGAGAG AAAGGTTGCG AAGTGTACGC GTTCTGCGGC GCCCTCTTCG GGATCTGCTC CATGATCACG CTGACGGTGA TCGCCATCGA CCGCTACTTC
Lethen087    AGCGTTGGAT TTTCGGGGAG AAGGGTTGTG AGCTGTATGC TTTCTGCGGA GCGCTCTTCG GCATCGCGTC CATGATCACC CTCGTGGTCA TCGCCACTGA CCGCTACCTG
Mastac088    AGAGGTGGAT CTTTGGCGAG AAAGCCTGTG AGCTCTACGC CTTCTGCGGC GCTCTCTTCG GCATCTGCTC CATGATCACG CTGACGGTTA TCGCCGTCGA CCGCTACTTC
Meleag089    GAGAGTGGAT CCTTGGAGAT ATAGGTTGTG ACTTGTACGC TTTCTGTGGG GCGCTCTTTG GAATAACCTC GATGATGACT CTGTTGGCTA TTTCTGTTGA CCGCTACTTG
Meleag090    AACGTTGGAT TTTTGGTGAG AAAGGCTGTG AGCTGTATGC CTTCTGCGGA GCTCTTTTTG GCATTACATC TATGATCACT TTGATGGTGA TTGCCTTGGA CAGATACTTT
Notech091    AGGAGTGGGT ACTTGGAGAC ACAGGTTGTA ACTTCTATGC TTTTTGTGGA GCTCTCTTCG GAATAACCTC AATGATGACC TTGCTAGCTA TTTCGGTGGA TCGCTACTGT
Opisth092    AACGCTGGAT TTTTGGTGAG AAAGGCTGTG AGCTGTATGC CTTCTGTGGA GCTCTTTTTG GCATTACATC TATGATCACT TTGATGGTGA TTGCCTTGGA CAGATATTTT
Pelodi093    AGCACTGGAT CTTTGGCAAA AAAGGTTGTG AGCTTTATGC CTTTTGCGGA GCCCTTTTTG GCATCACATC CATGATCACA CTGATGGCAA TTGCACTGGA CAGATATTTT
Phyllo094    AGCGGTGGCT CTTTGGGGAG GCAGGCTGCG AGTTCTATGC CTTCTGTGGG GCTCTCTTTG GCATCACCTC CATGATCACT CTGACAGCCA TCGCCCTGGA CCGCTACCTG
Physet095    AGCAGTGGCT CTTTGGAGAG GCAGGCTGTG AATTCTATGC CTTCTGTGGG GCTCTCTTTG GCATCACCTC CATGATTACC CTGACGGCCA TCGCCCTGGA CCGCTACCTG
Pipra_096    AACGCTGGAT TTTTGGTGAG AAAGGCTGTG AGCTGTATGC CTTCTGTGGA GCTCTCTTTG GCATTACATC TATGATCACT TTGATGGTGA TTGCCTTGGA CAGATATTTT
Podarc097    CAGAATGGAT ACTCGGAGAC ATAGGTTGTA ACTTTTATGC TTTTTGTGGG GCACTCTTTG GAATAACTTC AATGATGACT TTATTAGCTA TTTCAGTTGA TCGCTACTGT
Podarc098    AACGATGGGT ATTTGGTGAA AAGGGTTGCG AGCTGTACGC TTTCTGTGGA GCGCTTTTCG GCATCGCTTC CATGATCACT CTAACAGTGA TAGCCCTGGA CAGGTACTTT
Pseudo099    AGGAGTGGGT ACTTGGAGAC GCAGGTTGTA ACTTCTATGC TTTTTGTGGT GCTCTCTTCG GAATAACCTC AATGATGACC TTGTTAGCTA TTTCGGTGGA TCGCTACTGT
Python100    AGGAGTGGGT ACTTGGAGAC ACAGGTTGTA ACTTCTATGC TTTTTGTGGA GCACTCTTTG GAATAACCTC AATGATGACC TTGTTAGCTA TTTCAGTTGA TCGCTACTGT
Salmo_101    AGAGATGGAT CTTCGGAGAG AAAGGCTGTG AGCTGTATGC GTTCTGTGGA GCTCTATTTG GGATCTGCTC TATGATCACA CTGATGGTGA TAGCAGTGGA CCGCTACTTT
Salmo_102    AAGAGTGGAT TTTTGGCGAG TACGGATGCA AAATGTATGC CTTCTGTGGG GCTCTCTTTG GCATCACCTC CATGATCAAC CTGTTGGCTA TCTCCATTGA CCGCTACCTG
Salmo_103    AGGAATGGGT TTTTGGAGAA ACAGGCTGTA AGATGTACGC CTTCTGTGGG GCCTTATTTG GGATCACCTC CATGATCAAT CTGCTGGCTA TCTCCATTGA CCGCTACATC
Salmo_104    AGGAGTGGGT GTTTGGAGAC ACAGGCTGTA AGATGTACGC CTTCTGTGGG GCCTTATTTG GGATCACCTC CATGATCAAC CTGCTGGCTA TCTCCATTGA CCGCTACATA
Stegas105    AGAGGTGGAT CTTTGGAGAG AAAGGCTGCG AGCTCTACGC CTTCTGCGGC GCTCTCTTCG GGATCTGCTC CATGATCACG CTGACGGTGA TCGCGGTCGA CCGCTACTTC
Terrap106    AGCGCTGGAT TTTTGGCAAG AAAGGTTGTG AGCTGTATGC CTTCTGCGGA GCCCTTTTTG GCATTACATC CATGATCACA TTGATGGCAA TCGCACTGGA CAGATATTTT
Xenopu107    AAAGATGGAT CTTTGGAGAG AAAGGTTGCG AGCTATATGC TTTTTGTGGA GCCCTTTTTG GAATTACTTC CATGATCACA CTGATGGTGA TTGCTTTGGA TAGGTATTTT


             ....|....| ....|....| ....|....| ....|....| ....|....| ....|....| ....|....| ....|....| ....|....| ....|....| ....|....|
                    340        350        360        370        380        390        400        410        420        430        440
Felis000     GtGaTcAcGc AcCcAcTgGc CaCcAtCgG- --GgTgGtGt CcAaGaGgCg GgCgGcGcTt GtCcTgCtGg GcGtCtGgCt CtAtGcCcTa GcCtGgAgTc TgCcAcCcTt
Gallus001    GTGATCACCA AGCCCCTGCG CTCCATCCA- --GTGGACCT CCAAGAAGCG CACCATACAG ATTATTGCTG CTGTGTGGCT GTACTCGCTG GGGTGGAGCG TAGCCCCACT
Gallus002    GTCATCACAA AACCTCTGGC TTCTGTTCG- --AGTGATGT CAAAGAAGAA GGCCCTTATA ATCCTGGTAG GCGTCTGGCT GTACTCTTTG GCTTGGAGCC TCCCACCCTT
Gadus003     GTCATCACCA GGCCCCTGGA GGCATGAA-- --GTGGAACT CCAAGCGGAG AACCACCATC GCCATCTTGT TGGTCTGGCT CTACTCCCTG GCCTGGAGCC TAGCCCCTCT
Gadus_004    GTCATCACCA AGCCCCCGCA GGCCATCCG- --CTGGGTCT CGGGCCGGCG CACCATGGTG GTGATCCTGC TGGTCTGGCT CTACTCGCTG GCCTGGAGCC TGGCGCCCCT
Sminth005    GTGATCACCC GCCCTCTGGC CTCTATTGG- --GATGATCT CCAAGAAGAA GACCGGACTC ATCCTCCTGG GAGTCTGGTT GTACTCTCTG GCCTGGAGCC TTCCGCCTTT
Homo006  C   TAATCACACG CCCGCTGGCC ACCTTTGG-- -TGTGGCGTC CAAGAGGCGT GCGGCATTTG TCCTGCTGGG CGTTTGGCTC TATGCCCTGG CCTGGAGTCT GCCACCCTTC
Mus007  CT   GATCACACGT CCACTGGCCA CCATCGG--- CAGGGGATCC AAAAGACGAA CGGCACTCGT CCTGCTAGGC GTCTGGCTTT ATGCCCTGGC CTGGAGTCTG CCACCTTTCT
Spalax008    GTGATCACAC GCCCACTGGC CACCATCGG- --GGTGGCAT CCAAGAGGCG GACAGCACTT GTCCTGTTGG GTGTCTGGCT CTATGCCCTG GCCTGGAGTC TGCCCCCCTT
Monode009    GTCATTACTC GCCCTCTGGC CTCTATTGG- --TGTGATTT CAAAGAAGAA GACTGGCTTC ATCCTCCTGG GAGTCTGGTT GTACTCTTTG GCCTGGAGCC TTCCACCCTT
Rattus010    GTGATCACAC GTCCACTGGC CACCATCGG- --CATGAGAT CCAAGAGACG GACGGCACTA GTCCTGCTAG GTGTCTGGCT CTATGCCCTG GCCTGGAGTC TGCCGCCTTT
Podarc011    GTGATTACTA AGCCTCTGCA GTCTATAAA- --AAGGTCTT CAAAGAAACG CTCATGCATC ATCATTGCCT TTGTCTGGCT CTACTCGCTG GGATGGAGTG TATGTCCTCT
Trache012    GTGATCACAC GACCGCTGGC CTCCATTGT- --GGTGATGT CCAAGAAGAA GGCTTTGATA ATCCTGCTTG GAGTCTGGCT GTACTCTTTG GCTTGGAGCC TCCCACCCTT
Xenopu013    GTTATCACAA AACCACTACA GTCCATTCA- --ATGGTCTT CCAAAAAAAG AACATCACAG ATTATTGCTC TTGTCTGGAT GTATTCATTA ATGTGGAGCT TGGCACCATT
Danio014     GTGATCACGC GACCGCTGGC CTCCATCGG- --TGTGCTTT CTCAGAAGAG AGCTCTGCTC ATCCTCCTTG TAGCCTGGGT GTATTCACTC GGCTGGAGTC TGCCACCTTT
Danio_015    GCCATAACTC AGCCTCTCGC CCTAGTAAG- --AGTGACTA GCCGACGCAA AGCTGGAGCT GTGTTTGTCG TTGTGTGGCT CTACTCCCTG GGTTGGAGCC TTCCGCCTTT
Anolis016    GTGATTACGC GACCCCTTGC CTCCATCGG- --AGCAATGT CTACGAAGAA GGCTTTGCTA ATCCTATCAG GAGTCTGGCT GTATTCCTTA GCTTGGAGTC TCCCGCCTTT
Bos017  CT   GATCACACGC CCACTGGCCA CCGTCGG--- GATGGTGTCC AAGAGGCGGG CCGCGCTTGT CCTGCTGGGC GTCTGGCTCT ATGCCCTAGC TTGGAGCCTG CCGCCCTTCT
Callor018    GTCATAACAA AGCCATTACA GTCAATAAGC TCGACGACGA CAAAGAAGAA TACACTGAAG GTCATCATTC TGGTTTGGCT ATATTCGCTT GCATGGAGTC TTCCTCCACT
Canis019     GTCATCACGC ACCCCCTGGC CGCCGTCGG- --GGTGGTGT CCAAGAGGCG GGCGGCGCTC GTGCTGCTGG GCGTCTGGCT CTATGCCCTG GCCTGGAGTC TGCCGCCCTT
Cynogl020    GTCATCACTC GACCTCTGAC CTCCATCGG- --AGTTCTGT CAAAAAAGCG TGCGTTCCTG GTTCTTCTGG GCGCCTGGTT CTATTCCCTG GGCTGGAGTC TGCCTCCGTT
Dipodo021    GTGATCACAC GCCCACTGGC CACCATCGG- --GGTGACAT CCAAGAGACG AACTGCATTT GTCCTGCTGG GTGTCTGGGT CTATGCCCTG GCTTGGAGTC TTCCACCATT
Esox022  A   TCATCACCCG TCCGCTCTCC TCTATCGG-- -AGTGCTTTC TAAAAAGCGG GCCCGGCTTA TCCTCATGGC TGCCTGGGTC TACTCGCTCG GCTGGAGCCT GCCCCCTTTC
Haliae023    GTCATCACAA AACCTCTGGC TTCTGTTGG- --AGTGACAT CTAAGAAGAA GGCCCTAATA ATCCTGGTAG GAGTCTGGCT GTACTCTTTG GCTTGGAGCC TCCCGCCCTT
Macaca024    GTAATCACAC GCCCGCTGGC CACCATTGG- --TGTGGCGT CCAAGAGGCG CGCGGCATTT GTCCTGCTGG GCGTTTGGCT CTATGCCCTG GCCTGGAGTC TGCCACCCTT
Nototh025    GTGATCACCC GGCCTCTGGC CTCCCTGGG- --GGGGATGT CTCGCAGGAA AGCCCTGTGC ATCCTGGCTG CTGCCTGGGT CTACTCCGCA GGGTGGAGCC TGCCCCCCTT
Pygosc026    GTCATCACAA AGCCTCTGGC TTCTGTTGG- --AGTGACGT CTAAGAAGAA GGCCCTAATA ATCCTGGTAG GAGTCTGGCT GTACTCTTTG GCTTGGAGCC TCCTGCCCTT
Rutilu027    GCCATAACTC AGCCTCTTGC CTTGGTGGG- --CAACGTTA GCCGACGCAA AGCAGGCGCG GTGTTGGCCG TTGTGTGGCT CTACTCCCTG GGCTGGAGCC TTCCGCCTTT
Taenio028    ---------- ---------- ---------- ---------- ---------- ---------- ---------- ---------- ---------- -----GAGTC TCCCACCCTT
Taenio029    ---------- ---------- ---------- ---------- ---------- ---------- ---------- ---------- ---------- ---------- ----------
Tarsiu030    GTGATCACAC GCCCACTGGC CACCATCGG- --TGTGGCAT CCAAGAGGCG GGCAGCATTT GTCCTGCTTG GCGTCTGGCT CTATTCCCTG GCCTGGAGTC TGCCACCCTT
```

```
Tinamu031    GTCATCACAA AACCCCTGGC CTCTATCGG- --AGTGATGT CTAAGAAGAA GGCACTACTA ATCCTGGTAG GAGTCTGGCT GTACTCCTTG GCTTGGAGCC TCCCACCCTT
Trache032    GTGATCACTA AGCCTCTGCA ATCCATGCG- --GTGGACTT CCAAGAAACA CACATCGCAG ATCATTGTTA TTGTCTGGCT CTACTCGCTG GGATGGAGCG TGGCTCCGCT
Xenopu033    GTGATCACAC GACCACTCAC CTCCATTGG- --TGTTATGT CCAAGAAAAG GGCAGTGCTA ATCCTTTCAG GTGTTTGGCT GTATTCTCTG GCATGGAGTC TTCCTCCATT
Bjar_O034    GTGATTACCA AGCCTCTCCA GTCATTAAA- --AAGAAGTT CAAAGAAACG TTCGTGTATA ATCATTATGT TTGTCTGGCT CTACTCACTG GCTTGGAGTG TTTGTCCAAT
Cbic_O035    GTGATTACGA AGCCTCTCCA GTCCTTAAA- --AAGAAGTT CAAAGAAACG TTCTTGTATA ATCATTATAT TTGTCTGGCT CTACTCACTG GGTTGGAGTG TATGTCCATT
Cdur_O036    GTGATTACCA AGCCTCTCCA GTCATTAAA- --AAGAAGTT CAAAAAAACG TTCATGTATA ATCATTATGT TTGTCTGGCT CTACTCACTG GCTTGGAGTG TTTGTCCATT
Dips_O037    GTGATTACAA AGCCTCTCCA GTCCTTAAA- --AAGAAGTT CAAAGAAACG TTCTTGTATA ATCATTATAT TTGTCTGGCT CTACTCACTG GCTTGGAGTG TATGTCCATT
Eceph_038    GTGATTACAA AGCCTCTCCA GTCCTTAAA- --AAGAAGTT CAAAGAAACG TTCTTGTATA ATMATTATAT TTGTCTGGCT CTACTCACTG GGTTGGAGTG TATGTCCATT
Eund_O039    GTGATTACAA AGCCTCTCCA GTCCTTAAA- --AAGAAGTT CAAAGAAACG TTCTTGTATA ATCATTATAT TTGTCTGGCT CTACTCACTG GGTTGGAGTG TATGTCCATT
Ery_OP040    GTGATTACAA AGCCTCTCCA GTCCTTAAA- --AAGAAGTT CAAAGAAACG TTCTTGTATA ATCATTATAC TTGTCTGGCT CTACTCGCTG GGTTGGAGCG TGTGTCCATT
Hel_OP041    GTGATTACAA AGCCTCTCCA GTCCTTAAA- --AAGAAGTT CAAAGAAACG TTCTTGCATA ATCATTATAT TTGTCTGGCT CTACTCACTG GGTTGGAGTG TATGTCCATT
Lmil_O042    GTGATTACAA AACCTCTCCA GTCCTTAAA- --AAGAAGTT CAAAGAAACG TTCTTGTATA ATCATTATAC TTGTCTGGCT CTACTCGCTG GGTTGGAGCG TATGTCCATT
Mcor_O043    GTGATCACAA AACCTCTCCA GTCCTTAAA- --AAGAAGTA CAAAGAAACG CTCTTGTATA ATCATCATGT TTGTCTGGCT CTACTCACTG GGTTGGAGCG TATGTCCATT
Mlem_O044    GTGATCACAA AACCTCTCCA GTCCTTAAA- --AAGAAGTA CAAAGAAACG CTCTTGTATA ATCATCATGT TTGTCTGGCT CTACTCACTG GGTTGGAGCG TATGTCCATT
Oguib_045    GTGATTACAA AGCCTCTCCA GTCCTTAAA- --AAGAAGTT CAAAGAAACG TTCTTGTATA ATCATTATAT TTGTCTGGCT CTACTCACTG GGTTGGAGTG TATGTCCATT
Arizon046    GTGATTACGA AGCCTCTCCA GTCCTTAAA- --GAGAAGTT CAAAGAAACG TTCTTGTATA ATCATTATAT TTGTCTGGCT CTACTCACTG GGTTGGAGTG TGTGTCCATT
Cemoph047    GTGATTACGA AGCCTCTCCA GTCCTTAAA- --AAGAAGTT CAAAGAAACG TTCTTGCATA ATCATTATAT TTGTCTGGCT CTACTCACTG GGTTGGAGTG TGTGTCCATT
Hypsig048    GTAATTACAA AGCCTCTCCA GTCCTTAAA- --AAGAAGTT CAAAGAAACG TTCTTGTATA ATCATTATAT TTGTCTGGCT CTACTCACTG GCTTGGAGTG TATGTCCATT
Lampro049    GTGATTACGA AGCCTCTCCA GTCCTTAAA- --AAGAAGTT CAAAGAAACG TTCTTGTATA ATCATTATAT TTGTCTGGCT CTACTCACTG GGTTGGAGTG TGTGTCCATT
Pgutat050    GTGATTACGA AGCCTCTCCA GTCCTTAAA- --AAGAAGTT CAAAGAAACG TTCTTGTATA ATCATTATAT TTGTCTGGCT CTACTCACTG GGTTGGAGTG TGTGTCCATT
Mastic051    ---------- ---------- ---------- ---------- ---------- ---------- ---------- ---------- ---------- ---------- ----------
Ppat_O052    GTGATTACAA AGCCTCTCCA GTCCTTAAA- --AAGAAGTT CAAAGAAACG TTCTTGTATA ATCATTATAT TTGTCTGGCT CTACTCACTG GGTTGGAGTG TATGTCCATT
Smik_O053    GTGATTACAA AGCCTCTCCA GTCCTTAAA- --AAGAAATT CAAAGAAACG TTCTTGTATA ATCATTATAT TTGTCTGGCT CTACTCACTG GCTTGGAGTR TATGTCCATT
Snew_O054    GTGATTACAA AGCCTCTCCA GTCCTTAAA- --AAGAAGTT CAAAGAAACG TTCTTGTATA ATCATTATAT TTGTCTGGCT CTACTCACTG GCTTGGAGTG TATGTCCATT
Tae_OP055    GTGATTACAA AGCCTCTCCA GTCYTTAAA- --AAGAAGTT CAAAGAAACG TTCTTGTATA ATCATTATAT TTGTCTGGCT CTACTCACTG GGTTGGAGTG TATGTCCATT
Tham_O056    GTGATTACAA AGCCTCTCCA GTCCTTAAA- --AAGAAGTT CAAAGAAACG TTCTTGTATA ATCATTATAT TTGTCTGGCT CTACTCACTG GGTTGGAGTG TATGTCCATT
Tdor_O057    GTGATTACAA AGCCTCTCCA GTCCTTAAA- --AAGAAGTT CAAAGAAACG TTCTTGTATA ATCATTATAT TTGTCTGGCT CTACTCACTG GGTTGGAGTG TATGTCCATT
Anolis058    GTGATTACGA AGCCTCTCCA ATCCATAAA- --AAGGACAT CAAAAAAGCG CACCTGCATC ATCATTGTCT TTGTCTGGCT CTACTCACTT GGATGGAGTG TATGTCCACT
Pogona059    GTGATTACTA AGCCTCTGCA GTCCATAAA- --AAGGACTT CAAAGAAGCG CACATGTCTA ATCATTGTTT TTGTCTGGCT CTACTCGCTG GGATGGAGTG TGTGTCCACT
Protob060    GTGATTACAA AGCCTCTCCA GTCATTAAA- --AAGAAGTT CAAAGAAACG TTCATGTATA ATCATTATGT TTGTCTGGCT CTACTCACTG GCTTGGAGTG TTTGTCCATT
Thamno061    GTGATTACAA AGCCTCTCCA GTCCTTAAA- --AAGAAGTA CAAAGAAACG TTCTTGTATA ATCATTATAT TTGTCTGGCT CTACTCACTG GGTTGGAGTG TATGTCCATT
Alliga062    GTGATCACTA AACCGCTGCA ATCCATACG- --ATGGACTT CAAAGAGACG CACGGCACAG ATCATTGTTC TTGTCTGGCT TTACTCACTG GGATGGAGCA TGGCTCCACT
Athene063    GTCATCACAA AACCTCTGGC TTCTGTTGG- --AGTGACGT CTAAGAAGAA GGCCCTAATA ATCCTGGGAG GAGTCTGGCT GTACTCTTTG GCTTGGAGCC TCCCGCCCTT
Callor064    GTGATCACAC GACCCCTGGC CTCCATCGG- --GGTCCTCT CCCACAGACG AGCTGGGCTG ATCATCCTGT CCTTGTGGCT CTACTCGCTG GCCTGGAGCC TCCCTCCTTT
Chelon065    GTGATCACAC GACCACTGGC CTCCATTGT- --GGTGATGT CCAAGAAGAA GGCTTTGATA ATCCTTCTAG GAGTCTGGCT GTACTCTTTG GCTTGGAGCC TCCCACCCTT
Chelon066    GTGATCACTA AGCCTCTGCA ATCCATGCG- --GTGGACTT CCAAGAAACA CACATCGCAG ATCATTGTTA TTGTCTGGCT CTACTCGCTG GGATGGAGCG TGGCTCCACT
Chryse067    GTGATCACAC GACCGCTGGC CTCCATTGT- --GGTGATGT CCAAGAAGAA GGCTTTGATA ATCCTGCTAG GAGTCTGGCT GTACTCTTTG GCTTGGAGCC TCCCACCCTT
Danio_068    GCCATAACTC AGCCTCTCGC CCTAGTAAG- --CAGAGTTA GCCGACGCAA AGCTGGAGCT GTGTTGGTCG TTGTGTGGCT CTACTCCCTG GGTTGGAGCC TTCCGCCTTT
Danio_069    GTGATCACCA AACCACTGCA GGCCATCCG- --CTGGACAT CGGGACGCCG AACACTCATT ATTATCCTCG TGGTCTGGAT CTACTCGCTG GCCTGGAGTC TGGCGCCACT
Danio_070    GTGATCACTC GTCCTTTGGC CTCTATTGG- --CGTTATGT CACGCAAGCG AGCCTTGCTG ATCCTCTCTG CTGCCTGGGC TTACTCTATG GGCTGGAGCC TGCCCCCCTT
Danio_071    GTTATCACCA AACCACTGCA GACCATCCA- --GTGGAACT CCAAGCGCAG AACCGGTCTG GCCATCCTTT GCATCTGGCT CTACTCGCTG GCCTGGAGTC TGGCTCCTCT
Egrett072    GTCATCACAA AACCTCTGGC TTCTGTTGG- --AGTGATGT CTAAGAAGAA AGCCCTAATA ATCCTGGTAG GAGTCTGGCT GTACTCTTTG GCTTGGAGCC TCCCGCCCTT
Empido073    GTCATCACAA AACCTCTGGC TTCTGTTGG- --AGTGACAT CTAAGAAGAA GGCCCTAATA ATCCTGGTAG GAGTCTGGCT GTACTCTTTG GCTTGGAGTC TCCCACCCTT
Eptatr074    GTTATTACAC ATCCTTTGGC CTCTATCGCA AGAGGTCTAA CCAAGCGGAA CGCAGTGGTC GCCATGCTCT TCGTATGGAT GTTCTCCATT GGATGGAGCT TGCCGCCTAT
Eptesi075    GTGATCACGC GCCCACTGGC CATCGGCG-- ----TGGTGT CCAAAAGGCG GGCGGCGCTG GTCCTGCTGG GCGTCTGGCT CTACGCCCTG GCCTGGAGTC TTCCGCCTTT
Erpeto076    GTCATCACTA GGCCTCTGGC TTCAATGGG- --TGTGATGT CCCGCAAGCG TGCCCTGGTG ATCCTCTCAG GGGTGTGGCT CTACTCACTG GCCTGGAGCC TTCCACCATT
Falco_077    GTCATCACAA AACCTCTGGC TTCCGTTGG- --AGTGATGT CTAAGAAGAA GGCCCTAATA ATCCTGGTAG GAGTCTGGCT GTACTCTTTG GCTTGGAGCC TCCCGCCTTT
Gekko_078    GTGATTACGA GGCCTCTGCA GTCCATAAA- --GAGGACTT CAAAGAAAAA CTCATGCCTG ATCATTGTCT TTGTCTGGCT CTACTCACTG GCTTGGAGTG TGTGTCCGCT
Hippog080    GTCATCACGA GGCCTCTGAC CTCCATTGG- --CGTTTTGT CACGGAAACG GGCCCTCCTC GTACTTATGG CTGCATGGCT CTATTCTTTG GGGTGGAGCC TGCCGCCATT
Hippog081    GTGATCACCA GGCCTCTGGC CTCTCTGGG- --GGTGATGT CTCGCAGGAA AGCCCTTAGC ATCTTGGCTG TTGCCTGGGT CTACTCCATG GGCTGGAGCC TCCCCCCCTT
Hippog082    GTCATCACCA AGCCTCTGCA GGCGCTTCG- --ATGGACCT CGAAGAGACG CACCTGCATC TTTATTGCCC TGGTGTGGCT CTACTCATTA GCTTGGAGCC TTGCACCACT
Hippog083    GTTATCACCA AGCCCCTGCA GGCGATTCA- --CTGGAGCT CCAAGAGGAG AACTGCACTG GCAATCCTCA TGGTCTGGCT GTACTCTCTG GCCTGGAGTC TGGCTCCTCT
Ictalu084    GTTATCACTC GTCCTCTGGC CTCCATCGG- --CAAGATGT CACATAAGCG TGCATTTTGC ATCCTCGCCC TAGGCTGGCT GTATACCTTG TTCTGGAGCC TTCCTCCTTT
Ictalu085    GCCATAACCC GGCCTCTTTC TTTGCTGGG- --ACAGGTCA ATCAAGGCAG AGTTGGTACA GTGCTAGCCC TGGTATGGCT CTATGCTCTG GGCTGGAGTC TACCTCCTTT
Larimi086    GTCATCACGC GACCTCTGAC CTCGATCGG- --CGTGTTGT CACGGAAACG GGCCCTCTTC ATCCTCTTGG CAGCCTGGGC CTACTCTCTG GGATGGAGCC TGCCGCCGTT
Lethen087    GTGCTGACTC GGCCCCTCGC CTCCATAGG- --AGCCATGT CCAAACGGCG TGCAATGTAC ATCACGGCGG CCGTGTGGTT CTACTCGCTC GCCTGGAGCC TCCCACCCTT
Mastac088    GTCATCACGC GACCTCTGAC CTCCATCGG- --CGTGTTGT CGCGGAAACG GGCCCTCCTC ATCCTCCTGA CGTCCTGGGC TTACTCACTG GCCTGGAGCC TGCCGCCATT
Meleag089    GTGATCACCA AGCCCCTGCG CTCCATCCA- --GTGGACCT CCAAGAAGCG CACCATGCAG ATTATTGCTG CTGTGTGGCT GTACTCGCTG GGATGGAGCG TGGCCCCACT
Meleag090    GTCATCACAA AACCTCTGGC TTCTGTTCG- --AGTGATGT CTAAGAAGAA GGCCCTTATA ATCCTGGTAG GCGTCTGGCT GTACTCTTTG GCTTGGAGCC TCCCACCCTT
Notech091    GTGATCACAA AACCTCTCCA GTCCTTAAA- --AAGAAGTA CAAAGAAACG CTCTTATATA ATCATCATGT TTGTCTGGCT GTACTCACTG GGTTGGAGCG TATGTCCATT
Opisth092    GTCATCACAA AACCTCTGGC TTCTGTTGG- --AGTGACAT CTAAGAAGAA GGCCTTAATA ATCCTGGTAG GAGTCTGGCT GTACTCTTTG GCTTGGAGCC TCCCGCCCTT
Pelodi093    GTGATCACAC GACCCTTGGC CTCCATTGT- --GGTGATGT CGAAGAAGAA GGCTTTGATA ATCCTGCTAG CAGTCTGGCT GTACTCTTTG GCTTGGAGCC TTCCACCCTT
Phyllo094    GTGATCACAC GCCCATTGGC CACCATTGG- --GGTGGTGT CCAAGAGACG AGCAGCACTT GTCTTGCTGG GAGTCTGGCT CTATGCCCTG GCCTGGAGTC TTCCACCCTT
Physet095    GTGATCACAC GCCCACTGGC CACGGTCGG- --GATGGCAT CCAAGAGGCG GGCGGCGCTT GTCCTGCTGG GCGTCTGGCT CTATGCCCTG GCTTGGAGTC TGCCGCCCTT
Pipra_096    GTCATCACAA AACCTCTGGC TTCTGTTGG- --AGTGACAT CTAAGAAGAA GGCCCTAATA ATCCTGGTAG GAGTCTGGCT GTACTCTTTG GCTTGGAGTC TCCCACCCTT
Podarc097    GTGATTACTA AGCCTCTGCA GTCTATAAA- --AAGGTCTT CAAAGAAACG CTCATGCATA ATCATTGCCT TTGTCTGGCT CTACTCGCTG GGATGGAGTG TATGTCCTCT
Podarc098    GTGATTACGC GACCCTTGGC TTCCATTGG- --AGTAATGT CTAAGAAGAA AGCTTTGCTA ATCCTGTTAG GAGTCTGGCT GTATTCCTTA GCATGGAGCC TTCCTCCCTT
Pseudo099    GTGATCACAA AACCTCTCCA GTCCTTAAA- --AAGAAGTA CAAAGAAACG CTCTTATATA ATCATCATGT TTGTCTGGCT GTACTCACTG GGTTGGAGCG TATGTCCATT
Python100    GTGATTACTA AGCCTCTGCA GTCCATAAA- --AAGGAGTT CAAAGAAACG CTCATGTCTA ATCATTATGT TTGTCTGGCT TTACTCACTG GGTTGGAGTG TGTGTCCATT
Salmo_101    GTGATTACAC GTCCACTGGC CTCCATCGG- --GGTGCTGT CCAAGAAAAG AGCCCTGCTC ATCCTCATGG CTGCCTGGGT CTACTCATTG GGATGGAGCC TGCCCCCTTT
Salmo_102    GTCATCACCC AGCCCCTCAA GGCCCTTGG- --CCGGTCGT CAAGGCGTCG GACCACCCTG GCAATACTAA TGGTGTGGCT TTACTCTTTT GCCTGGAGTT TTGCCCCACT
Salmo_103    GTCATCACCA AGCCCCTGCA GGCCCTCCA- --CTGGACCT CTAAACGCCG TACCTCCGTT GTCATCGTCA TTGTCTGGCT CTACTCGCTG GCCTGGAGCC TGGCGCCTCT
Salmo_104    GTCATCACCA AGCCCCTGCA GGCCCTGCA- --TTGGACCT CTAAACGCCG CACCTCCGTC GTCATCGTCA TAGTCTGGCT CTACTCATTG GCCTGGAGCC TGGCACCTCT
Stegas105    GTCATCACGC GACCCCTCAC CTCCATCGG- --CGTGCTGT CACGGAAACG GGCCCTGCTC ATCCTGATGG CGGCCTGGGC CTACTCGCTG GGGTGGAGCC TGCCGCCGTT
Terrap106    GTGATCACAC GACCGCTGGC CTCCATTGT- --GGTGATGT CCAAGAAGAA GGCTTTGATA ATCCTGCTAG GAGTCTGGCT GTACTCTTTG GCTTGGAGCC TCCCACCCTT
Xenopu107    GTGATCACAC GACCACTCAC CTCTATTGG- --TGTTATGT CCAAGAAAAG GGCAGTGCTA ATCCTTTCAG GTGTTTGGCT GTATTCTCTG GCATGGAGTC TGCCTCCCTT


             ....|....| ....|....| ....|....| ....|....| ....|....| ....|....| ....|....| ....|....| ....|....| ....|....| ....|....|
                     450        460        470        480        490        500        510        520        530        540        550
Felis000     CtTtGgCtGg aGcGcCtAcG tAcCaGaGgG gCtGcTgAcC tCcTgCtCcT gGgAcTaCaT gAgCtTcAcG cCaTcAgTtC gAgCcTaCaC cAtGcTgCtC tTcTgCtTtG
Gallus001    CCTTGGGTGG AGTTCCTACG TGCCTGAGGG CTTGATGATA TCCTGTACAT GGGACTATGT AACCTACTCC CCTGCAAATA GAAGTTACAC CATGATTTTA TGCTGCTGCG
Gallus002    CTTTGGATGG AGTGCCTATG TTCCTGAAGG CCTGCTGACT TCCTGTTCCT GGGATTACAT GACTTTCACA CCATCAGTCC GTGCCTACAC AATGCTGCTT TTCTGCTTTG
Gadus003     CGTTGGCTGG AGCTCCTACA TCCCAGAGGG CCTGAGGACC TCATGCACCT GGGACTACGT CACTTACACG GCGTCCAACA GGAGCTACAC CATGATGCTG TGCTGTTTCG
Gadus_004    GCTGGGTTGG AGCTCCTACA TCCCAGAGGG TCTGATGACA TCATGCACAT GGGATTACGT GACGTCCACC CCGGCCAATA AGGGCTACAC GTTGATGCTG TGCTGCTTCG
Sminth005    CTTTGGATGG AGTGCCTACG TCCCTGAAGG GCTGCTCACG TCTTGTTCCT GGGATTACAC AACCTTCACA CCATCTGTCC GTGCTTATAC CATCCTGCTC TTCTGCTTCG
Homo006  C   TTCGGCTGGA GCGCCTACGT GCCCGAGGGG TTGCTGACAT CCTGCTCCTG GGACTACATG AGCTTCACGC CGGCCGTGCG TGCCTACACC ATGCTTCTCT GCTGCTTCGT
Mus007  CT   TTGGTTGGAG TGCCTACGTG CCCGAGGGGC TGCTGACATC CTGCTCCTGG GACTACATGA CCTTCACACC CCAGGTGCGT GCCTACACCA TGCTGCTCTT CTGCTTTGTC
Spalax008    CTTTGGCTGG AGTGCCTACG TGCCTGAGGG GCTGCTGACC TCCTGCTCCT GGGACTACAT GACCTTCACA CCCTCAGTGC GTGCCTACAC CATGCTGCTC TTCTGCTTCG
Monode009    CTTTGGATGG AGTGCCTATG TTCCTGAGGG GCTGCTCACA TCTTGTTCCT GGGATTACAC AACCTTCACG CCATCGGTCC GGGCGTACAC CATGCTGCTT TTTTGTTTTG
Rattus010    CTTTGGCTGG AGCGCCTACG TGCCCGAGGG GCTGCTGACA TCCTGCTCCT GGGACTACGT GACCTTCACG CCCCTCGTGC GCGCCTACAC CATGCTGCTC TTCTGCTTTG
Podarc011    CTTTGGATGG AGTTCCTATA TACCTGAAGG TTTGATGATA TCTTGTACAT GGGACTACGT ATCCTATTCT CCAGCAAACA GAAGTTATAC CATGATGTTA TGTTGCTTTG
Trache012    CTTTGGATGG AGTGCTTATG TTCCTGAGGG TTTGTTGACT TCCTGTTCCT GGGACTACGT TACCTTTACC CCATCAGTCC GTGCCTATAC AATGCTCCTC TTCTGCTTTG
Xenopu013    ACTTGGCTGG AGTTCCTATG TGCCAGAGGG CTTAAGGATA TCTTGTACAT GGGACTATGT GACATCTACA ATGTCAAACA GAAGTTACAC AATGATGCTG TGCTGTTGCG
Danio014     CTTTGGCTGG AGTGCTTATG TTCCCGAAGG GCTGTTGACC TCTTGTACCT GGGATTATAT GACTTTCACA CCCTCTGTGC GAGCTTACAC TATGCTCCTC TTCATCTTCG
Danio_015    CTTTGGCTGG AGTGCTTATG TCCCTGAGGG GCTTCAGACG TCTTGTTCTT GGGATTACAT GACCTTCACC CCATCGGTGC GTGCGTACAC CATCCTCCTC TTTGTTTTTG
Anolis016    CTTTGGATGG AGTGCTTATG TTCCAGAGGG CCTCTTAACC TCCTGTTCCT GGGATTATAT CACTTTTACC CCATCAGTCC GTGCCTACAC GATGCTCCTT TTCTGCTTTG
Bos017  CT   TTGGCTGGAG TGCCTATGTG CCCGAAGGCC TGCTGACCTC TTGCTCCTGG GACTACGTGA GCTTCACGCC GTCCGTCCGC GCCTACACCA TGCTGCTCTT CTGTTTTGTG
Callor018    CCTTGGTTGG AGTTCCTATA TTCCTGAAGG TCTGATGACT TCCTGCACCT GGGATTACGT GACAAATTCT CCATCTAACA GGAGCTACAC TTTGATGCTC TGCTGCTTTG
Canis019     CTTCGGCTGG AGTGCCTACG TCCCCGAGGG GCTGCTGACC TCCTGTTCCT GGGACTACAT GAGCTTCACG CCGTCGGTCC GCGCCTACAC CATGCTGCTC TTCTGCTTCG
Cynogl020    CTTCGGCTGG AGTGCCTACG TCCCAGAGGG TCTGATGACC TCCTGTACGT GGGACTACAT GACCTTTACC CCCTCTGTGC GGGCCTACAC CATGCTGCTC TTCATCTTTG
Dipodo021    CTTTGGCTGG AGTGCCTATG TGCCCGAGGG GCTGCTCACA TCCTGCTCCT GGGACTACAT GACCTTCACC CCCTCTGTGC GCTCCTACAC CATGTTGCTC TTCTGCTTCG
Esox022  A   TTTGGCTGGA GTGCCTATGT TCCAGAGGGC CTGTTGACCT CCTGCACCTG GGACTATGTG ACCTTCACCC CTTCAGTGCG TGCTTACACC ATGCTTCTCT TCACGTTTGT
Haliae023    CTTTGGATGG AGTGCTTATG TTCCCGAGGG TCTGCTGACT TCTTGTTCCT GGGACTACAT GACTTTCACA CCATCAGTCC GTGCCTACAC AATGCTGCTT TTCTGCTTCG
Macaca024    CTTTGGCTGG AGTGCCTATG TGCCCGAGGG GTTGCTGACA TCCTGCTCCT GGGACTACAT GAGCTTCACA CCGGCTGTGC GTGCCTACAC CATGCTTCTC TGCTGCTTCG
Nototh025    CTTTGGCTGG AGTGCTTATG TCCCAGAAGG TCTGATGACG TCCTGTTCCT GGGATTACAT GACATTTACT CCTTCTGTCC GTTCCTACAC CATGCTGCTC TTTATCTTCG
Pygosc026    CTTTGGATGG AGTGCGTATG TTCCCGAGGG TCTGCTGACT TCCTGTTCCT GGGACTACAT GACTTTCACA CCATCAGTCC GCGCCTACAC GATGCTGCTT TTCTGCTTCG
Rutilu027    CTTTGGCTGG AGCGCTTATG TTCCAGAGGG TCTTCAGACT TCCTGTTCTT GGGACTATAT GACCTTCACT CCATCAGTTC GAGCGTACAC CATCTTGCTC TTCATTTTTG
Taenio028    CTTTGGATGG AGTGCATATG TTCCTGAAGG TTTGCTGACT TCCTGTTCCT GGGACTATAT GACTTTCACA CCATCAGTCC GTGCCTACAC GATGCTGCTT TTCTGCTTTG
Taenio029    --TTGGGTGG AGTTCCTACG TGCCTGAGGG CTTGATGATA TCCTGTACAT GGGACTATGT AACCTACTCC CCTGCAAACA GAAGCTACAC CATGATTTTA TGCTGCTGTG
Tarsiu030    CTTTGGCTGG AGTGCCTATG TGCCCGAGGG GCTGCTGACG TCCTGTTCCT GGGACTACAT GAGCTTCACA CCGTCTGTCC GCGCCTACAC CATGCTTCTC TGCTGCTTCG
Tinamu031    CTTTGGATGG AGTGCGTATG TTCCCGAGGG CCTGCTGACC TCCTGTTCCT GGGATTACAT AACCTTCACC CCATCAGTCC GTGCCTATAC AATGCTGCTT TTCTGCTTTG
Trache032    TCTTGGGTGG AGTTCCTATG TGCCAGAAGG GTTGATGATA TCTTGTACAT GGGACTATGT AACCTATTCC CCAGCAAACA GAAGTTACAC CATGATGTTA TGTTGCTGTG
Xenopu033    CTTTGGTTGG AGTGCATATG TACCCGAGGG TCTTCTGACA TCCTGCACCT GGGATTATAT GACCTTTACT CCATCTGTTC GAGCATATAC CATGCTTCTC TTCTGTTTTG
Bjar_O034    ATTCGGGTGG AGTTCCTATA TCCCAGAAGG TTTGATGATA TCCTGTACAT GGGACTATAT AAGCTATTCT CCAGCAAACA GAAGTTACAC CATGTTGCTA TGTTGGTGTG
Cbic_O035    ATTCGGGTGG AGTTCCTATA TCCCAGAAGG TTTGATGATA TCCTGTACAT GGGACTATGT AAGCTATTCT CCAGGAAACA GAAGTTACAC CATGTTGCTA TGTTGGTGTG
Cdur_O036    ATTTGGGTGG AGTTCCTATA TCCCAGAAGG TTTGATGATA TCCTGTACAT GGGACTATGT AAGCTATTCT CCAGCAAACA GAAGTTACAC CATGTTGCTA TGTTGGTGTG
Dips_O037    ATTCGGGTGG AGTTCCTATA TCCCAGAAGG TTTGATGATA TCCTGTACAT GGGACTATGT AAGCTATTCT CCAGCAAACA GAAGTTATAC CATGTTGCTA TGTTGGTGTG
Eceph_038    ATTYGGGTGG AGTTCCTATA TCCCAGAAGG TTTGATGATA TCCTGTACAT GGGACTATGT AAGCTATTCT CCAGGAAACA GAAGTTACAC GATGTTGCTA TGTTGGTGTG
Eund_O039    ATTCGGGTGG AGTTCCTATA TCCCAGAAGG TTTGATGATA TCCTGTACAT GGGACTATGT AAGCTATTCC CCAGGAAACA GAAGTTACAC GATGTTGCTA TGTTGGTGTG
Ery_OP040    ATTTGGGTGG AGTTCCTATA TCCCAGAAGG TTTAATGATA TCCTGTACAT GGGACTATGT AAGCTATTCT CCAGGAAACA GAAGTTACAC TATGTTGCTA TGTTGGTGTG
Hel_OP041    ATTCGGGTGG AGTTCCTATA TCCCAGAAGG TTTGATGATA TCCTGTACAT GGGACTATGT AAGCTATTCT CCAGCAAACA GAAGTTACAC TATGTTGCTA TGTTGGTGTG
Lmil_O042    ATTTGGGTGG AGTTCCTATA TCCCAGAAGG TTTGATGATA TCCTGTACGT GGGACTATGT AAGCTATTCT CCAGGAAACA GAAGTTACAC TATGTTGCTA TGTTGGTGTG
```

```
Mcor_O043     ATTCGGGTGG AGTTCCTATA TCCCCGAAGG TTTGATGATA TCCTGTACAT GGGACTATGT AAGCTATTCT CCAGCAAACA GAAGTTACAC CATGTTGCTA TGTTGGTGTG
Mlem_O044     ATTCGGGTGG AGTTCCTATA TCCCAGAAGG TTTGATGATA TCCTGTACAT GGGACTATGT AAGCTATTCT CCAGCAAACA GAAGTTACAC CATGTTGCTA TGTTGGTGTG
Oguib_045     ATTCGGGTGG AGTTCCTATA TCCCAGAAGG TTTGATGATA TCCTGTACAT GGGACTATGT AAGCTATTCT CCAGCAAACA GAAGTTACAC TATGTTGCTA TGCTGGTGTG
Arizon046     ATTCGGGTGG AGTTCCTATA TCCCAGAAGG TTTGATGATA TCCTGTACAT GGGACTATGT AAGCTATTCT CCAGGAAACA GAAGTTACAC CATGTTGCTA TGTTGGTGTG
Cemoph047     ATTCGGGTGG AGTTCCTATA TCCCAGAAGC TTTGATGATA TCCTGTACAT GGGACTATGT AAGCTATTCT CCAGGAAACA GAAGTTACAC CATGTTGCTA TGTTGGTGTG
Hypsig048     ATTCGGGTGG AGTTCCTATG TCCCAGAAGG TTTGATGATA TCCTGTACAT GGGACTATGT AAGCTATTCC CCAGCAAACA GAAGTTATAC CATGTTGCTA TGTTGGTGTG
Lampro049     ATTCGGGTGG AGTTCCTATA TCCCAGAAGG TTTGATGATA TCCTGTACAT GGGACTATGT AAGCTATTCT CCAGGAAACA GAAGTTACAC CATGTTGCTA TGTTGGTGTG
Pgutat050     ATTCGGGTGG AGTTCCTATA TCCCAGAAGG TTTGATGATA TCCTGTACAT GGGACTATGT AAGCTATTCT CCAGGAAACA GAAGTTACAC CATGTTGCTA TGTTGGTGTG
Mastic051     ---------- -GTTCCTATA TCCCAGAAGG TTTGATGATA TCCTGTACAT GGGACTATGT AAGCTATTCT CCAGGAAACA GAAGTTACAC CATGTTGCTA TGTTGGTGTG
Ppat_O052     ATTCGGGTGG AGTTCCTATA TCCCAGAAGG TTTGATGATA TCCTGTACAT GGGACTATGT AAGCTATTCT CCAGGAAACA GAAGTTACAC TATGTTGCTA TGTTGGTGTG
Smik_O053     ATTCGGGTGG AGTTCCTATA TCCCAGAAGG TTTGATGATA TCCTGTACAT GGGACTATGT AAGCTATTCT CCAGCAAACA GAAGTTATAC CATGTTGCTA TGTTGGTGTG
Snew_O054     ATTCGGGTGG AGTTCCTATA TCCCAGAAGG TTTGATGATA TCCTGTACAT GGGACTATGT AAGCTATTCT CCAGCAAACA GAAGTTATAC CATGCTGCTA TGTTGGTGTG
Tae_OP055     ATTCGGGTGG AGTTCCTATA TCCCAGAAGG TTTGATGATA TCCTGTACAT GGGACTATGT AAGCTATTCT CCAGGAAACA GAAGCTACAC TATGTTGCTA TGTTGGTGTG
Tham_O056     ATTCGGGTGG AGTTCCTATA TCCCAGAAGG TTTGATGATA TCCTGTACAT GGGACTATGT AAGCTATTCT CCAGCAAACA GAAGTTACAC TATGTTGCTA TGTTGGTGTG
Tdor_O057     ATTCGGGTGG AGTTCCTATA TCCCAGAAGG TTTGATGATA TCCTGTACAT GGGACTATGT AAGCTATTCT CCAGCAAACA GAAGTTACAC TATGTTGCTA TGTTGGTGTG
Anolis058     CTTTGGGTGG AGTTCCTATA TACCAGAGGG TTTGATGATA TCCTGTACAT GGGACTATGT GACCTATTCT CCAGCAAATC GAAGTTATAC TATGATGTTG TGTTGCTGCG
Pogona059     TTTTGGGTGG AGTTCCTATA TACCTGAAGG CTTGATGATA TCTTGTACTT GGGATTATGT GACCTATTCT CCAGCAAACA GAAGTTATAC CATGATGTTA TGCTGCTGTG
Protob060     ATTCGGGTGG AGTTCCTATA TCCCAGAAGG TTTGATGATA TCCTGTACAT GGGACTATGT AAGCTATTCT CCAGCAAACA GAAGTTACAC CATGTTGCTA TGTTGGTGTG
Thamno061     ATTCGGATGG AGTTCCTATA TCCCAGAAGG TTTGATGATA TCCTGTACAT GGGACTATGT AAGCTATTCT CCCGGAAACA GAAGTTACAC CATGTTGCTA TGTTGGTGTG
Alliga062     ATTTGGGTGG AGTTCTTATG TGCCTGAGGG TTTGATGATA TCCTGTACTT GGGACTACAT AACCACTACC CCAGCAAACA GAACCTATAC CATGATGCTG TGTTGCTGTG
Athene063     CTTTGGATGG AGTGCGTATG TTCCCGAGGG TCTGATGACT TCCTGTTCCT GGGACTACAT GACTTTCACA CCGTCAGTCC GTGCCTACAC GATGCTGATT TTCTGTTTCG
Callor064     CTTCGGCTGG AGTGCCTATG TGCCTGAAGG CTTGCTAACC TCCTGTACCT GGGACTATAT GACATTCACA CCATCTGTCC GGGCTTACAC TATGCTTCTC TTCTGCTTTG
Chelon065     CTTTGGATGG AGTGCTTATG TTCCTGAGGG TTTGTTGACT TCCTGTTCCT GGGACTACGT GACCTTTACC CCATCAGTCC GTGCCTATAC AATGCTCCTC TTCTGCTTTG
Chelon066     TCTTGGGTGG AGTTCCTATG TGCCAGAAGG GTTGATGATA TCTTGTACAT GGGACTATGT AACCTATTCC CCAGCAAACA GAAGTTACAC CATGATGTTA TGTTGCTGTG
Chryse067     CTTTGGATGG AGTGCTTATG TTCCGGAGGG TTTGTTGACT TCCTGTTCCT GGGACTACGT GACCTTTACC CCATCAGTCC GTGCCTATAC AATGCTCCTC TTCTGCTTTG
Danio_068     CTTTGGCTGG AGTGCTTATG TCCCTGAGGG GCTTCAGACG TCTTGTTCTT GGGATTACAT GACCTTCACC CCATCAGTGC GTGCGTACAC CATCCTCCTC TTTGTTTTTG
Danio_069     TATAGGATGG AGTTCCTACA TCCCAGAGGG CCTCATGACC TCCTGCACGT GGGACTATGT GACGTCCACT CCTGCCAACA AGAGCTACAC CTTGATGCTC TGCTGTTTCG
Danio_070     TTTTGGATGG AGTGCATATG TTCCTGAAGG TCTGCTGACG TCCTGTTCGT GGGACTACAT GACCTTCAGT CCTTCTGTTC GAGCCTACAC AATGCTGCTC TTCACTTTTG
Danio_071     GATTGGCTGG AGTTCCTACA TCCCTGAAGG TCTGATGACG TCTTGCACAT GGGACTACGT CTCTCCATCT CCTGCCAACA AGAGCTACAC CATGATGCTC TGCTGTTTCG
Egrett072     CTTTGGATGG AGTGCATATG TGCCTGAGGG TCTGCTGACT TCCTGTTCCT GGGACTACAT GACTTTCACA CCATCAGTCC GCGCCTACAC GATGCTGCTT TTCTGCTTTG
Empido073     CTTTGGATGG AGTGCATATG TTCCTGAGGG GCTGCTGACT TCCTGTTCCT GGGACTACAT GACTTTCACA CCATCAGTCC GTGCCTACAC GATGTTGCTT TTCTGCTTTG
Eptatr074     CTTCGGATGG AGTAGCTACG CTCCTGAGGG ATTGATGACC TCCTGCACAT GGGATTATAT TACTTTCACA CCGACGGTGC GCACCTACAC CATGCTGCTT TTCTGCTTCG
Eptesi075     CTTCGGCTGG AGTGCCTACG TGCCCGAGGG CCTGCTGACC TCCTGCTCCT GGGACTACAT GAGCTTCACG CCAGCGGTCC GCGCCTACAC CATGCTGCTC TTCTGCTTCG
Erpeto076     CTTTGGATGG AGTGCCTATG TGCCAGAAGG GCTCCTGACT TCCTGTACCT GGGATTATAT GACATTTACC CCAGCAGTGC GTGCTTATAC AATGCTGCTT TTTACCTTCA
Falco_077     CTTTGGATGG AGTGCGTACG TTCCTGAGGG TCTGCTGACC TCCTGTTCCT GGGACTACAT GACTTTCACA CCATCAGTCC GTGCCTACAC AATGCTGCTT TTCTGCTTTG
Gekko_078     GTTTGGGTGG AGTTCATATG TGCCTGAGGG TTTGATGATT TCCTGTACAT GGGACTATAT AACCTATTCT CCAGCAAACA GAAGCTACAC CATGATGTTA TGTTGGTGTG
Hippog080     CTTTGGCTGG AGCGCCTATG TCCCGGAGGG TCTGCTGACT TCCTGTTCCT GGGACTACAT GACCTTTACG CCGTCGGTGC GAGCGTACAC CATGCTGCTT TTCATCTTCG
Hippog081     CTTTGGCTGG AGTGCCTATG TCCCGGAGGG TCTGATGACG TCCTGCTCCT GGGACTACAT GACGTTCACC CCTTCTGTCC GCTCCTACAC GATGCTGCTC TTCACCTTCG
Hippog082     TTTGGGTTGG AGTTCGTACA TTCCCGAGGG CCTGATGACC TCGTGCACGT GGGACTATGT GACATCGACT CCAGCCAATA AAAGTTACAC TTTGATGTTA TGCTGCTTTG
Hippog083     CGTCGGCTGG AGCTCTTATA TCCCAGAGGG GCTGATTACG TCTTGTACGT GGGATTACGT CACATACACA TTGGCCAACA GGAGCTACAC GATGATGCTT TGCTGTTTTG
Ictalu084     TTTTGGATGG AGTGCATATG TTCCTGAGGG CTTGATGACC TCCTGCACAT GGGACTATAT GACGTTTACC CCATCTGTTC GTGCCTACAC AATGCTACTC TTCGTCTGTG
Ictalu085     CTTCGGCTGG AGTGCGTATG TTCCCGAGGG TCTCCAGACC TCCTGCTCTT GGGACTACAT GTCATTCACG CCATCGGTGC GCACCTACAC CATTCTTCTC TTCATGTTCG
Larimi086     CTTCGGCTGG AGTGCCTATG TCCCCGAGGG CCTGATGACG TCTTGCACGT GGGACTACAT GACCTTTACC CCGTCGGTGC GAGCGTACAC CATGCTGCTC TTCATCTTCA
Lethen087     CTTTGGCTGG AGTGCCTACG TCCCAGAGGG ACTCATGACG TCGTGCACAT GGGATTACGT GACATTCACA CCAGCCGTAC GCTCCTACAC CATGCTGCTC TTCTGCTTCG
Mastac088     CTTCGGCTGG AGTGCCTATG TCCCAGAGGG TCTGATGACT TCGTGTACGT GGGACTACAT GACGTTTACC CCGTCGGTGC GAGCGTACAC CATGCTGCTC TTCATCTTCG
Meleag089     CCTTGGGTGG AGTTCCTACG TGCCTGAGGG CTTGATGATA TCCTGTACGT GGGACTATGT AACCTACTCC CCTGCAAACA GGAGTTACAC CATGATTTTA TGCTGCTGCG
Meleag090     CTTTGGATGG AGCGCCTATG TTCCTGAAGG CCTGCTGACT TCCTGTTCCT GGGATTACAT GACTTTCACA CCATCAGTCC GTGCCTACAC AATGCTGCTT TTCTGTTTTG
Notech091     ATTCGGGTGG AGTTCCTATA TCCCAGAAGG TTTGATGATA TCCTGTACAT GGGACTATGT AAGCTATTCT CCATCAAACA GAAGTTACAC CATGTTGCTA TGTTGGTGTG
Opisth092     CTTCGGATGG AGTGCGTATG TTCCCGAGGG TCTGCTGACT TCCTGTTCCT GGGACTACAT GACTTTCACA CCTTCAGTCC GCGCCTACAC GATGCTGCTT TTCTGCTTCG
Pelodi093     CTTTGGATGG AGTGCTTATG TTCCTGAGGG TTTGTTAACT TCCTGCTCCT GGGACTATGT GACCTTTACT CCATCAGTCC GTGCCTATAC AATGCTCCTC TTCTGCTTTG
Phyllo094     CTTTGGCTGG AGTGCCTATG TGCCCGAGGG GCTGCTGACC TCCTGTTCCT GGGACTATGT GAGCTTCACG CCATCGGTCC GTGCCTACAC CATGCTGCTC TTCTGCTTTG
Physet095     CTTTGGCTGG AGTGCCTATG TGCCCGAGGG GCTGCTGACC TCTTGCTCCT GGGACTACGT GAGCTTCATG CCATCGGTCC GCGCCTACAC CATGCTGCTC TTCTGCTTTG
Pipra_096     CTTTGGATGG AGTGCATATG TTCCTGAGGG GCTGCTGACT TCCTGTTCCT GGGACTACAT GACTTTCACC CCATCAGTCC GCGCCTACAC GATGCTGCTT TTCTGCTTTG
Podarc097     CTTTGGATGG AGTTCCTATA TACCTGAAGG TTTGATGATA TCTTGTACAT GGGACTACGT ATCCTATTCT CCAGCAAACA GAAGTTACAC CATGATGTTA TGTTGCTTTG
Podarc098     CTTCGGATGG AGTGCTTATG TTCCGGAAGG CCTGCTGACA TCTTGCTCCT GGGATTATAT TACCTTCACC CCATCGGTCC GTGCCTACAC AATGCTCCTC TTTTGCTTTG
Pseudo099     ATTTGGGTGG AGTTCCTATA TCCCAGAAGG TTTGATGATA TCCTGTACAT GGGACTATGT AAGCTATTCT CCATCAAACA GAAGTTACAC CATGTTGCTA TGTTGGTGTG
Python100     ATTTGGGTGG AGTTCCTATA TCCCAGAAGG TCTGATGATA TCTTGTACTT GGGACTATGT AACCTATTCT CCAGCAAACA GAAGTTACAC CATGTTGCTA TGTTGCTGTG
Salmo_101     CTTTGGCTGG AGCGCCTATG TTCCAGAGGG CCTGTTGACC TCCTGTTCAT GGGACTATGT GACCTTTACC CCATCAGTGC GTGCCTACAC CATGCTGCTC TTCACCTTTG
Salmo_102     CGTTGGCTGG AGCTCCTATA TCCCAGAGGG CCTCATGACT TCCTGCACTT GGGATTATGT CACATACACG TACGCCAACC AGAGCTACAC CATGATGCTG TGTTGTTTTA
Salmo_103     CTTTGGCTGG AGTTCTTATA TCCCTGAGGG CCTGATGACC TCCTGTACGT GGGATTATGT GACCTCCACA CCAGCCAATA GGAGTTACAC TCTGATGCTA TGCATCTTTG
Salmo_104     CCTGGGCTGG AGTTCTTATA TCCCGGAGGG CCTGATGACA TCTTGTACGT GGGATTATGT GACCTCCACA CCAGCCAATA GGAGCTACAC TCTAATGCTA TGCGTCTTTG
Stegas105     CTTCGGCTGG AGCGCCTACG TCCCAGAGGG TCTGCTGACT TCCTGTACGT GGGACTACAT GACCTTCACC CCGTCGGTGC GAGCCTACAC CATGCTGCTC TTCATCTTCG
Terrap106     CTTCGGATGG AGTGCTTATG TTCCTGAGGG TTTGTTGACT TCCTGTTCCT GGGACTACGT GACCTTTACC CCATCAGTCC GTGCCTATAC AATGCTCCTC TTCTGCTTTG
Xenopu107     CTTTGGTTGG AGTGCATATG TGCCCGAGGG TCTCCTGACA TCCTGCACCT GGGATTATAT GACCTTCACT CCATCTGTTC GAGCATATAC CATGCTTCTC TTCTGTTTTG


              ....|....| ....|....| ....|....| ....|....| ....|....| ....|....| ....|....| ....|....| ....|....| ....|....| ....|....|
                     560        570        580        590        600        610        620        630        640        650        660
Felis000      tGtTcTtCtT gCcCcTgCtG gTcAtCgTcT aCtGcTaCaT cTtCaTcTtC aGgGcCaTcC gGgAgAcAgG cCaGgCtCtC cAgAcCtTcA gGgCcTgC-- ----------
Gallus001     TATTTTTTAT TCCCCTGATA ATAATACTCC ATTGCTATTT ATTTATGTTC TTGGCCATAA GAAGTACTGG CAGAGATGTC CAAAAGCTTG GGTCC----- ----------
Gallus002     TCTTCTTCAT TCCTTTGATT GCTATCATAT ACAGCTATGT CTTTATATTT GAGGCTATCA AGAAGGCCAA CAAGTCTGTA CAGACATTTG GTTGCAAAC- ----------
Gadus003      TGTTCTTCAT CCCCCTGGCT ATCATCTCCT ACTGCTACCT CTTCATGTTC CTGGCCATCC GCAAAACAAG CAGAGATGTG GAGCGGCTGG GCATCCAAG- ----------
Gadus_004     TGTTCTTCAT CCCTCTGGGT ATAATATCCT ACTGCTATCT GTGCATGTTC CTGGCTATAC GCAGTGCCGG CAGAGAGATC GAGAGGCTGG GGACACAGG- ----------
Sminth005     TCTTCTTCAT CCCACTGACT GTAATTATCT ACTGCTACAT CTTCATCTTC AGGGCCATTA AGGACACCAA CAAGGCCGTT CAGAACATTG GATCTAG--- ----------
Homo006  C    GTTCTTCCTC CCTCTGCTTA TCATCATCTA CTGCTACATC TTCATCTTCA GGGCCATCCG GGAGACAGGA CGGGCTCTCC AGACCTTCGG GGCCTGCA-- ----------
Mus007  CT    TTCTTCCTCC CCCTGCTCAT CATCATCTTC TGCTACATCT TCATCTTCAG GGCCATCCGA GAGACAGGCC GGGCCTGTGA GGGCTGCGGT GAGTCCC--- ----------
Spalax008     TGTTCTTCCT TCCCCTGCTC ATCATCATCT TCTGCTACAT CTTTATCTTC AAAGCCATCC GAGAGACAGG CCGGGCCTGT GAGGGCTGTG GTGAGTCCC- ----------
Monode009     TCTTCTTCAT TCCCCTGATT GTAATCATCT ACTGCTACAT CTTCATCTTC AGAGCCATTC AGGACACCAA CAAGGCTGTT CACAGTATCG GATCTGG--- ----------
Rattus010     TCTTCTTCCT CCCTCTGCTC ATTATCATCT TCTGCTACAT CTTCATCTTC AGGGCCATTC GAGAGACAGG CCGGGCCTGT GAGGGCTGTG GTGAGTCCC- ----------
Podarc011     TGTTCTTTAT CCCTCTGATA ATAATCTTCC ACTGCTATCT ATTCATGTTT CTGGCCATTA GAAGTACTGG CAGGAACGTT CAGAAGTTAG GATCAACC-- ----------
Trache012     TCTTCTTCAT TCCTCTGATT GCCATCATAT ACAGTTATGT CTTTATCTTC AAGGCTATCA AGAATACCAA TGAGGCTGTT CAGAACATTG GTTCTGAT-- ----------
Xenopu013     TCTTCTTTAT TCCACTTATT GTAATATCAC ATTGTTACTT ATTCATGTTT CTGGCAATTC GGAGTACTGG CAGGAATGTA CAGAAACTTG GATCA----- ----------
Danio014      TCTTCTTCAT TCCACTCATC GTCATCATCT ACTGCTACTT CTTCATTTTC CGATCCATTC GCACTACCAA TGAAGCTGTG GGCAAAATTA ATGGAG---- ----------
Danio_015     TATTCTTCAT CCCACTAGGC ATTATAGGCA GCTGCTACTT CGCAATTTTT CAAACAATCC GAGCAGCGGG GAAGGAAATT CGAGAGCTGG ATT------- ----------
Anolis016     TATTCTTTAT TCCCCTAATT GCCATCATAT ACAGTTATGT GTTCATTTTC ATAGCCATCA AGAATTCAAA CAGGGCTGTT CAAAGGACCA ATTCAGAC-- ----------
Bos017  CT    TTCTTCCTCC CCTTGCTCAT CATCATTTAC TGCTACATCT TCATCTTCAA GGCCATCCGA GAGACGGGCC AAGCTCTCCA GACCTTCGGG ACCTGCG--- ---------A
Callor018     TGTTCTTCGT GCCACTGATT GTAATTTCCT ACTGTTACAT CTGTATGTTC AGGGCTGTAA AACACACCAC CAGGGATATT GAGAAGATGG GAGGCTGCA- ----------
Canis019      TGTTCTTCCT GCCGCTGCTG GTCATCGTCT ACTGCTACGT GTTCATCTTC AGGGCCATCC GGGAGACAGG CCAGGCTCTC CAGACCTTCA GGGCCTGTG- ----------
Cynogl020     TCTTCTTCCT GCCGCTCTTC ATCATCATCT ACTGCTACAT CTTCATCTTT CGTGCGATCC GTAACACCAA CCAGGCCGTG GGGAAGGTGA ACGGCAGCGT GCACAGTCAC
Dipodo021     TGTTCTTCCT CCCCCTGCTC GTCATCATCT ATTGCTACAT CTTCATCTTC AGAGCCATCC GGGAGACACG TCGGGCCTGC GAGGGCTGTG GTGAGTCCC- ----------
Esox022  A    CTTCTTCCTC CCACTCATTG TCATTATGTA CTGCTACTTC TTCATCTTCA GAGCCATACG GACAACTGAA AGGGCTGTAA CCAAGATTAA TGGCACCG-- ----------
Haliae023     TCTTCTTCAT TCCTTTGATT GCTATCATAT ACAGCTATGT CTTTATATTT GAGGCTATCA AGAAGGCCAA CAAGTCTGTT CAGACATTTG GATGCAAAC- ----------
Macaca024     TGTTCTTCCT CCCTCTGCTT ATCATCATCT ACTGCTACAT CTTCATCTTC AGGGCCATCC GGGAGACAGG ACAGGCTCTC CAGACCTTCG GGGCCTGCA- ----------
Nototh025     TCTTCTTCAT CCCTCTCTTC ATCATCATCT TCAGCTACTT CTGCATCTTC AGAGCCATCC GACACACAAC CAGAGCGATA ACACAGATGA ACTGTC---- ----------
Pygosc026     TCTTTTTCAT TCCTTTGATT GCTATCATAT ACAGCTATGT CTTTATATTT GAGACTATCA AGAAGGCCAA CAAGTCTATT CAGACGTTTG GATGCAAAC- ----------
Rutilu027     TGTTCTTCAT CCCACTAGGC ATTATTGTCA GCTGCTACGT TGGAATCTTC CAAGCTATCC GAGCAATGGG AAAGGAAATT AGAGAGTTGG ATT------- ----------
Taenio028     TCTTTTTCAT TCCATTGATA GCTATCATAT ACAGCTATGT CTCCATCTTT GAGGCTATCA AGAAGGCCAA CAAGTCTATT CAGACATTTG GATGCAAAT- ----------
Taenio029     TGTTTTTTAT CCCCCTGGTA ATAATATTCC ACTGCTATTT ATCGATGTTC CTGGCCATAA GGCGTACTGG CAGAGATGTT CAAAACCTGG GGTCC----- ----------
Tarsiu030     TGTTCTTCCT TCCCCTACTT ATCATCATCT ACTGCTACAT CTTCATTTTC AGGGCCATCC GGGAGACAGG AAGGGCTCTC CAGAACTTCG GAGCCTGCA- ----------
Tinamu031     TCTTTTTCAT TCCTTTGATT GCTATCATAT ACAGCTATAT ATTTATCTTT GAGGCTATCA AGAAAGCCAA CAAGTCTGTT CAGACACTTG GATGCAAAC- ----------
Trache032     TGTTTTTCAT TCCCCTGGTT ATAATATTCC ATTGCTATTT ATTCATGTTT CTGGCCATAA GAAGTACTGG CAGGGATGTT CAGAAGCTAG GGTCC----- ----------
Xenopu033     TTTTCTTCAT TCCACTTTTT ATCATTATAT ACTGCTACAT CTTTATCTTC AAGGCCATCA AGAATACAAA CAGGGCAGTT CAGAAAATAG GTACAG---- ----------
Bjar_O034     TGTTCTTCAT TCCTCTCATA ATAATCTTCC ATTGCTATTT ATTCATGTTC CTCACCATCA GAAGGACAGG CAGGAATGTT CAGAAATTAG GATCCAGT-- ----------
Cbic_O035     TGTTCTTTAT TCCTCTTATG ATAATCTTCC ATTGCTATTT ATTCATGTTC CTCACCATCA GAAGTACAGG CAGGAATGTT CAGAAATTGG GATCTAGC-- ----------
Cdur_O036     TGTTCTTCAT TCCTCTCATA ATAATCTTCC ATTGCTATTT ATTCATGTTC CTCACCATAA GAAGTACAGG CAGGAATGTT CAGAAATTAG GATCCAGT-- ----------
Dips_O037     TGTTCTTCAT TCCACTTATG ATAATCTTCC ATTGCTATTT ATTCATGTTC CTCACCATCA GAAGTACAGG CAGGAATGTT CAGAAATTGG GATCCAGC-- ----------
Eceph_038     TGTTCTTCAT TCCTCTTATG ATAATCTTCC ATTGCTATTT ATTCATGTTC CTCACCATCA GAAGTACAGG CAGGAATGTT CAGAAATTGG GATCCAGC-- ----------
Eund_O039     TGTTCTTCAT TCCTCTTATG ATAATCTTCC ATTGCTATTT ATTCATGTTC CTCACCATCA GAAGTACAGG CAGGAATGTT CAGAAATTGG GATCCAGC-- ----------
Ery_OP040     TGTTCTTCAT TCCTCTTATG ATCATCTTCC ATTGCTATTT ATTCATGTTC CTCACCATCA GAAGTACAGG CAGGAATGTT CAGAAATTGG GATCCAGC-- ----------
Hel_OP041     TGTTCTTCAT TCCTCTTATG ATAATCTTCC ATTGCTATTT ATTCATGTTC CTCACCATCA GAAGTACAGG CAGGAATGTT CAGAAATTGG GATCCAGC-- ----------
Lmil_O042     TGTTCTTCAT TCCTCTTATG ATCATCTTCC ATTGCTATTT ATTCATGTTC CTCACCATCA GAAGTACAGG CAGGAATGTT CAGAAATTGG GATCCAGC-- ----------
Mcor_O043     TGTTCTTCAT TCCTCTTATG ATAATCTTCC ATTGCTATTT ATACATGTTC CTCACCGTAA GAAGTACAGG CAGGAACGTT CAGAAATTAG GATCTAGC-- ----------
Mlem_O044     TGTTCTTCAT TCCTCTTATG ATAATCTTTC ATTGCTATTT ATTCATGTTC CTCACCATAA GAAGTACAGG CAGGAATGTT CAGAAATTAG GATCTAGC-- ----------
Oguib_045     TGTTCTTCAT TCCTCTTATG ATAATCTTCC ATTGCTATTT ATTCATGTTT CTCACCATCA GAAGTACAGG CAGGAATGTT CAGAAATTGG GATCCAGC-- ----------
Arizon046     TGTTCTTCAT TCCTCTTATG ATAATCTTCC ATTGCTATTT ATTCATGTTC CTCACCATCA GAAGTACAGG CAGGAATGTT CAGAAATTGG GATCTAGC-- ----------
Cemoph047     TGTTCTTCAT TCCTCTTATG ATAATCTTCC ATTGCTATTT ATTCATGTTC CTCACCATCA GAAGTACAGG CAGGAATGTT CAGAAATTGG GATCTAGC-- ----------
Hypsig048     TGTTCTTCAT TCCTCTTATG ATAATCTTCC ATTGCTATTT ATTCATGTTC CTCACCATCA GAAGTACAGG CAGGAATGTT CAGAAATTGG GATCCAGC-- ----------
Lampro049     TGTTCTTCAT TCCTCTTATG ATAATCTTCC ATTGCTATTT ATTCATGTTC CTCACCATCA GAAGTACAGG CAGGAATGTT CAGAAATTGG GATCTAGC-- ----------
Pgutat050     TGTTCTTCAT TCCTCTTATG ATAATCTTCC ATTGCTATTT ATTCATGTTC CTCACCATCA GAAGTACAGG CAGGAATGTT CAGAAATTGG GATCTAGC-- ----------
Mastic051     TGTTCTTCAT TCCTCTTATG ATAATCTTCC ATTGCTATTT ATTCATGTTC CTCACCATCA GAAGTACAGG CAGGAATGTT CAGAAATTGG GATCTAGC-- ----------
Ppat_O052     TGTTCTTCAT TCCTCTTATG ATAATCTTCC ATTGCTATTT ATTCATGTTC CTCACCATCA GAAGTACAGG CAGGAATGTT CAGAAATTGG GATCCAGC-- ----------
Smik_O053     TGTTCTTCAT TCCACTTATG ATAATCTTCC ATTGCTATTT ATTCATGTTC CTCACCATCA GAAGTACAGG CAGGAATGTT CAGAAATTGG GATCCAGC-- ----------
Snew_O054     TGTTCTTCAT TCCACTTATG ATAATCTTCC ATTGCTATTT ATTCATGTTC CTCACCATCA GAAGTACAGG CAGGAATGTT CAGAAATTGG GATCCAGC-- ----------
```

```
Tae_OP055    TSTTCTTCAT TCCTCTTATG ATAATCTTCC ATTGCTATTT ATTCATGTTC CTCACCATCA GAAGTACAGG CAGGAATGTT CAGAAATTGG GATCCAGC-- ----------
Tham_O056    TGTTCTTCAT TCCTCTTATG ATAATCTTCC ATTGCTATTT ATTCATGTTC CTCACCATCA GAAGTACAGG CAGGAATGTT CAGAAATTGG GATCCAGC-- ----------
Tdor_O057    TGTTCTTCAT TCCTCTTATG ATAATCTTCC ATTGCTATTT ATTCATGTTC CTCACCATCA GAAGTACAGG CAGGAATGTT CAGAAATTGG GATCCAGC-- ----------
Anolis058    TGTTCTTTAT TCCCCTCGTC ATCATCTTCC ACTGCTATAT ATTCATGTTT CTGGCCATAA GAAGTACAGG CAGGAATGTT CAAAAACTGG GTTCAACC-- ----------
Pogona059    TGTTTTTTAT CCCACTGGTC ATCATCTTCC ATTGCTATCT ATTCATGTTT CTGGCTATAA GAAATACTGG CAGGAATGTT CAGAAATTGG GATCAACA-- ----------
Protob060    TGTTCTTCAT TCCTCTTATA ATAATCTTCC ATTGCTATTT ATTCATGTTC CTCACCATCA GAAGTACAGG CAGGAATGTT CAGAAATTAG GATCCAGT-- ----------
Thamno061    TGTTCTTCAT TCCTCTTATG ATAATCTTCC ATTGCTATTT ATTCATGTTC CTCACCATCA GAAGTACAGG CAGGAATGTT CAGAAATTGG GATCCAGC-- ----------
Alliga062    TATTTTTCAT TCCTCTGCTT ATAATATTCC ATTGCTATTT ATCCATGTTT ATGGCTATAA GAAATACTGG CAGACATGTT CAGAAGCTAG GGTCC----- ----------
Athene063    TCTTTTTCAT TCCTTTGATT GCTATCATAT ACAGCTACGT CTTTATATTT GAGGCTATCA AGAAGGCCAA CAAGTCTATT CAGATATTTG GATGTAAAC- ----------
Callor064    TCTTCTTCAT CCCACTGGGA GTCATCATCT GCTGTTACAT CTTCATCTTC AGGACCATCC AATCCACCAA CAAGGCTGTG GAAAAAGTCG GGGGAAGCA- ----------
Chelon065    TCTTCTTCAT TCCTCTGATT GCCATCATAT ACAGTTATGT CTTTATCTTC AAGGCTATCA AGAATGCCAA TGAGGCTGTT CAGAACATTG GTTCTGAT-- ----------
Chelon066    TGTTTTTCAT TCCCCTGGTT ATAATATTCC ATTGCTATTT ATTCATGTTT CTGGCCGTAA GAAGTACTGG CAGGGATGTT CAGAAGCTAG GGTCC----- ----------
Chryse067    TCTTCTTCAT TCCTCTGATT GCCATCATAT ACAGTTATGT CTTTATCTTC AAGGCTATCA AGAATACCAA TGAGGCTGTT CAGAACATTG GTTCTGAT-- ----------
Danio_068    TATTCTTCAT CCCACTAGGC ATTATAGGCA GCTGCTACTT CGCAATTTTT CAAACAATCC GAGCAGCGGG GAAGGAAATT CGAGAGCTGG ATT------- ----------
Danio_069    TGTTCTTCAT TCCTCTAGGG ATCATCTCGT ACTGCTACTT CTTCATGTTC CTCGCCATTC GGAGCGCAAG CAGGGATGTG GAGAAGCTGG GTAGCCATG- ----------
Danio_070    TATTCTTCAT TCCCCTCTTC GTCATCATTT ATTGCTACTT CTTTATCTTC AAGGCCATCC GTGAAACCAA CAGAGCTGTG GGGAAAATCA ATGGAGAAG- ----------
Danio_071    TCTTCTTCAT CCCACTGAGC ATCATCCTCT ACTGTTACCT GTTCATGTTC CTCTCCGTGC GGCAGGCCAG CAGAGATCTG GAAATGCTGA GCTCTCA--- ----------
Egrett072    TCTTTTTCAT TCCTTTGATT GCTATCATAT ACAGCTATGT CTTTATATTT GAGGCTATCA AGAAGGCCAA CAAGTCTATT CAGACATTCG GATGCAAAC- ----------
Empido073    TCTTTGTCAT TCCTTTGATT GCCATCATAT ACAGCTATGT CTTTATATTT GAGGCTATCA AAAAGGCCAA CAAGTCTGTT CAGACATTTG GAAGCAAAC- ----------
Eptatr074    TCTTCTTCAT TCCACTATTG ATCATCATTG TTTGCTACGT GTGCATCTTC ATCGCCATCA AAAGGACAAG CAGGGAGGTG CAGTCTCTTG GCTCCTCAG- ----------
Eptesi075    TGTTCTTCCT CCCGCTGCTG GTCATCATCT ACTGCTACGT CTTCATCTTC AGGGCCATTC GGGAGACAGG CCAGGCTCTC CAGACGTTCG GAGCCTGCG- ----------
Erpeto076    TTTTTTTTAT TCCTCTGCTT GTCATTATCT GCTGCTACTT TTTCATCTTC CGTGCCATCC GTTCAACCAA CAGGGCTATT AAAAAAATCA GTTCTGATG- ----------
Falco_077    TCTTCTTCAT TCCTTTGATT GCTATCATAT ACAGCTATGT CTTTATATTT GAGGCTATCA AGAAGGCCAA CAAGTCTGTT CAGACATTTG GATGCAAAA- ----------
Gekko_078    TGTTCTTCAT TCCTCTGATC ATAATCTTCC ATTGCTATAT ATTCATGTTT CTTGCCATAA GAACTACAGG CAGGAATGTT CAGAAGTTAG GATCATCC-- ----------
Hippog080    TCTTCTTCCT GCCACTCTTC ATCATCATCT ACTGCTACGT CTTCATCTTC CGCGCCATAC GCAACACAAA CCAGGCTGTG GGGAAGTTTA ACGGCAGCAC TCACAGTCAC
Hippog081    TCTTCTTCAT CCCTCTCTCC ATCATCATCT TCTGCTACTG CTGCATCTTC AGGGCCATCC GACACACAAC ACGAGCGATA GAAAAGATCA ACTGTG---- ----------
Hippog082    TATTCTTCAT CCCTCTGGGA ATCATATCCT ACTGTTATCT GTGCATGTTC CTGGCCATCC GCCGTGCAAG CAGAGATGTG GAGAAGTGGG GGTCCCAGG- ----------
Hippog083    TTTTCTTTAT TCCTCTGGGA ATCATCTTTT ATTGCTACCT CCTCATGTTT CTGGCCATAA GGAAGACTGG CAGGGAGGTA GAGCGTCTGG GGACTCAGG- ----------
Ictalu084    TCTTCTTCAT CCCTCTGTTT GTCATTATGT ACTGCTATTT CTGCATCTTC CGTGCTATTC GCAGCACCAC CAGTGCTGTG GGTAAAATTA GTGAGGAAGG A---------
Ictalu085    TCTTCTTCAT CCCTCTGAGC ATCATTGCTG CCTGTTACTT GGTCATCTTC CGAGCTATCC GAAAAACCCG GAGAGAAGTA AGACAGCTTG ACT------- ----------
Larimi086    TCTTCTTCCT GCCGCTCTTC ATCATCATCT ACTGCTACGT CTTCATCTTC CGCGCCATCC GCAGCACCAA CCAGGCTGTG GGGAAGATTA ACGGCAGCAT TCACAGTCAC
Lethen087    TCTTCTTCAT CCCGCTCATT GTCATCATCT TCTGCTACGT GCGCATCTTC GTAGCCATCA AGAACACCAA CAGGGCTGTG AAGACTCTGG GGGAAACCC- ----------
Mastac088    TCTTCTTCCT GCCGCTCTTT GTCATCATCT ACTGCTACGT CTTCATCTTC CGCGCCATCC GCACCACCAA CCAGGCTGTG GGGAAGGTTA ACGGCAGCGT GCACAGCCAC
Meleag089    TATTTTTTAT TCCACTGCTA ATAATACTCC ACTGCTATTT ACTTATGTTC TTGGCCATAA GAAGTACTGG CAGAGATGTC CAAAAGCTTG GGTCC----- ----------
Meleag090    TCTTCTTCAT TCCTTTGATT GCTATCATAT ACAGCTATTT CTTTATATTT GAGGCTATGA AGAAGGCCAA CAAGTCTATA CAGACATTTG GATGCAAAC- ----------
Notech091    TGTTCTTCAT TCCTCTTATG ATAATCTTCC ATTGCTATTT ATTCATGTTC CTCACCATAA GAAGTACAGG CAGGAATGTT CAGAAATTAG GATCCACC-- ----------
Opisth092    TCTTTTTCAT TCCTTTGATT GCTATCATAT ACAGCTATGT CTTTATATTT GAGGCTATCA AGAAGGCCAA CAAGTCTATT CAGACATTTG GATGCAAAC- ----------
Pelodi093    TCTTCTTCAT TCCTCTAATT GCCATCATAT ATAGTTATGT CTTTATCTTC AAGGCTATCA AGAATGCCAA CATGGCTGTT CAGAACATTG GCTCTGAT-- ----------
Phyllo094    TGTTCTTCCT TCCCCTGCTT GTCATCATCT ACTGCTACGT CTTCATCTTC AGGGCCATTC GGGAGACAGG CCAGGCTCTC CAGACTTTCG GGTCCTGCG- ----------
Physet095    TGTTCTTCTT GCCCCTGCTC ATCATCATCT ACTGCTACAT CTTCATCTTC AAGGCCATCC GGGAGACGGG CCAAGTTCTC CAGACTTTCG GTGCCAGCG- ----------
Pipra_096    TCTTTTTCAT TCCTTTGATT GCCATCATAT ACAGCTACGT CTTTATATTT GAGGCTATCA AGAAGGCCAA CAAGTCTGTT CAGACATTTG GAAGCAAAC- ----------
Podarc097    TGTTCTTTAT CCCTCTGATA ATAATCTTCC ACTGCTATCT ATTCATGTTT CTGGCCATTA GAAGTACTGG CAGGAACGTT CAGAAGTTAG GATCAACC-- ----------
Podarc098    TGTTCTTCAT TCCCCTAATT GCCATCATAT ACAGCTATTT CTTCATTTTC AGAGCCATCC GGAATACAAA CAGGGCTGTT CAAACCATCA GTTCAGATG- ----------
Pseudo099    TGTTCTTCAT TCCTCTTATG ATAATCTTCC ATTGCTATTT ATTCATGTTC CTCACCATAA GAAGTACAGG CAGGAATGTT CAGAAATTAG GATCCACC-- ----------
Python100    TGTTCTTCAT TCCACTGATA ATAATCTTCC ACTGCTATTT ATGCATGTTT CTAGCCATAA GAAATACTGG CAGGAATGTT CAGAAATTAG GATCCAGC-- ----------
Salmo_101    TCTTCTTCAT CCCCCTCATC GTCATCATGT ACTGCTACTT CTTCATCTTC AGAGCCATAC GTACAACTAA CAAGGCTGTA ACCAAGATCA ACTGCAATG- ----------
Salmo_102    CCTTCTTCAT CCCACTGGGA GTCATCTTCT ACTGCTACCT CTTCATGTTC CTGGCCATCC GCACTGCCAG CAGGGAAGTA GAGAACTTGG GGACACAAG- ----------
Salmo_103    TGTTCTTCAT CCCACTGGCC ATCATCTCCT ACTGCTACCT CTGCATGTTC CTGGCCATAC GCACTGCCAG TAGAGACATG GAGAAACTGG GCAGTCAGG- ----------
Salmo_104    TGTTCTTCAT CCCTCTGGGC ATCATCTCTT ACTGCTACCT CTGCATGTTC CTGGCCATAC GCACTGGTAG TAGAGACATG GAGAAGCTGG GCAGTCAGG- ----------
Stegas105    TCTTCTTCCT GCCGCTCTTC ATCATCATCT ACTGCTACGT CTTCATCTTC CGAGCCATCC GCAACACCAA CCAGGCTGTG GGGAAGATTA ACGGCAGCAC TC--------
Terrap106    TCTTCTTCAT TCCTCTGATT GCCATCATAT ACAGTTATGT CTTTATCTTC AAGGCTATCA AGAATACCAA TGAGGCTGTT CAGAACATTG GTTCTGAT-- ----------
Xenopu107    TTTTCTTCAT TCCTCTTTTT ATCATTATAT ATTGCTACAT CTTTATCTTC AAGGCCATCA AAAATACAAA CAGGGCAGTT CAGAAAATAG GCACAG---- ----------


             ....|....| ....|....| ....|....| ....|....| ....|....| ....|....| ....|....| ....|....| ....|....| ....|....| ....|....|
                    670        680        690        700        710        720        730        740        750        760        770
Felis000     gAgGgTgGtG gTaGgTcCcC cCgGcAaCgG cAgCgGcTgC aGaGaGaGtG gAaAaTgGcC aAgAtCgAgC tGcTgGtCaT cCtTcTcTtC gTgCtCtCcT gGgCcCcCtA
Gallus001    ------TGCA GCCGCAAATC CTTCCTCTCT CAGTCCATGA AGAATGAATG GAAACTGGCA AAAATTGCGT TTGTGGTCAT CATTGTGTAT GTCTTGTCCT GGTCTCCATA
Gallus002    -ATGGAAACA GAGAGCTCCA GAAACAGTAT CATAGGATGA AGAATGAGTG GAAGTTGGCC AAGATTGCAC TGATCGTCAT CTTGCTTTAT GTCATTTCCT GGTCACCATA
Gadus003     ----TGCGGA AATCCACCAT AATCCGCCAG AAGTCCATCC GAACTGAATG GAAGTTGGCA AAGATTGCCT TTGTGGTGAT CGTGGTTTAT GTCTTATCCT GGTCACCGTA
Gadus_004    ----TGAGGA AGTCCACGCT GATGCAGCAG CAGACCATCA AGACCGAGTG GAAACTGACC AAAGTTGCCT TTGTCGTCAT CATCGTTTAT GTCCATTCAT GGTCGCCGTA
Sminth005    --TGAGCACA CTCCATCCCT GAGGCATTTT CAAAGGATGA AGAATGAATG GAAGATGGCA AAGATTGCCC TGGTGGTCAT CCTACTCTTT GTGTTGTCCT GGGCCCCTTA
Homo006  C   AGGGCAATGG CGAGTCCCTG TGGCAGCGGC AGCGGCTGCA GAGCGAGTGC AAGATGGCCA AGATCATGCT GCTGGTCATC CTCCTCTTCG TGCTCTCCTG GGCTCCCTAT
Mus007  CT   -CTCTGCGGC AGA----GGC GGCAGTGGCA GCGGCTGCAG AGTGAGTGGA AGATGGCCAA GGTCGCACTG ATTGTCATTC TTCTCTTCGT GCTGTCCTGG GCTCCCTACT
Spalax008    ---CTCAGCG GC-------G GCGACAGTGG CAGCGGCTGC AGAATGAGTG GAAGATGGCC AAGGTCGCAC TGCTGGTCAT CTTCCTCTTT GTGCTCTCCT GGGCCCCCTA
Monode009    --TGAGAGCA CGGCATCCCC AAGGCACTGT CAAAGGATGA AGAATGAATG GAAGATGGCA AAGATCGCCC TGGTGGTTAT CCTCCTTTAT GTGCTGTCCT GGGCCCCCTA
Rattus010    ---CTCTGCG GC-------G GCGGCAGTGG CAGCGGCTAC AGAGTGAATG GAAGATGGCC AAGGTCGCAC TGATCGTCAT TCTCCTCTTT GTGCTGTCCT GGGCTCCCTA
Podarc011    ------TATA ACCGGAAGTC GAATGTTTCA CAGTCAGTGA AGAGTGAATG GAAGTTAGCA AAAATTGCCT TTGTGGCTAT TGTGGTGTTT GTTCTGTCCT GGTCTCCCTA
Trache012    ---GCTAATA AAGTATCCCA GAGACAGTAT CAGAGGATGA AGAATGAATG GAAAATGGCC AAAATTGCCT TGATTGTCAT CTTACTTTAC GTCATTTCCT GGTCACCATA
Xenopu013    ------TACG GTCGCCAATC TTTTTTGTCT CAATCCATGA AGAATGAATG GAAAATGGCA AAAATTGCTT TTGTAATCAT CATTGTATTT GTACTGTCCT GGTCTCCTTA
Danio014     -ATAACAAAC GAGACTCGAT GAAACGATTC CAACGACTGA AAAATGAATG GAAGATGGCA AAAATTGCTC TTATTGTTAT TCTTATGTAT GTCATCTCCT GGTCTCCATA
Danio_015    -------GCG GGGAAACGCA CAAGGTGTAT GAACGCATGC AGAACGAATG GAAGATGGCG AAAGTCGCTC TTGTAGTCAT TTTGCTTTTT ATTATATCCT GGTCGCCCTA
Anolis016    ---AACAGTA AAGAAGGTCA AAAACTCTAC CAGAAGCTGA AGAATGAGTG GAAAATGGCC AAAGTAGCCT TGATTGTCAT CTTGCTCTAT GTCATCTCCT GGTCTCCATA
Bos017  CT   GGGTGGCAGT GAGTGTCCCC GGCAGCGGCA GCGGCTACAG AATGAGTGGA AAATGGCCAA GATCGAGCTG CTGGTCATCC TCCTCTTTGT GCTCTCCTGG GCCCCCTACT
Callor018    -GAAGAAAAG CGTATGAAGC CCATC---AA CAAACAGTAA AGGTTGAATG GAAATTGGCC AAAATTGCAT TTGTGGCGAT TGTCGTGTTT GTGCTCTCGT GGTTCCCTTA
Canis019     -AGGGCGGGG CCAGGTCCCC CCGACAGCGG CAGCGGCTAC AGAGAGAGTG GAAGATGGCC AAGATGGAGC TGCTGGTCAT CCTGCTCTTC GTGCTCTCCT GGGCCCCCTA
Cynogl020    AGCAGCGGAC GGAACTTAGT GAAGAGCCGC CACCGTCTCC AGAACGAGTG GAAAATGGCC AAGATCGCTC TGATGGTCAT CCTCCTGTAC GTCATCTCCT GGTCGCCGTA
Dipodo021    ---------- CACAGCAACG GCAGCAGTGG CAGCGGCTGC AGAGCGAGTG GAAGATGGCC AAGATCGCAC TGCTGGTCAT CCTCCTCTTT GTGCTTTCCT GGGCCCCCTA
Esox022  A   GAAACACCAG AGACTCCATC AAGAGTTTTC ACCGGCTGAA GAACGAGTGG AAGACGGCTA AGGTCGCCCT GATTGTCATC CTCTTTTACG TCATCTCCTG GTCCCCCTAC
Haliae023    -ATGGAAATA AAGAGTTCCA GAAACAGTAT CAGAGGATGA AAAATGAGTG GAAGATGGCC AAAATTGCAC TGATTGTCAT CTTGCTTTAT GTCATTTCCT GGTCACCATA
Macaca024    -AGGGCAGTG GCGAGTCCCT GTGGCAGCGG CAGCGGCTGC AGAGCGAGTG CAAGATGGCC AAGATCATGC TGCTGGTCAT CCTCCTCTTC GTGCTCTCCT GGGCTCCCTA
Nototh025    -AGGGAACCA GAGACTCTGC AAAGAAGTTC AATAAGATGA AGACTGAATG GAAGATGGCA AAGATCGCAC TCATCGTCAT CCTGCTATAC GTCATCTCTT GGGCCCCCTA
Pygosc026    -GTGGAAATA AAGAGTTCCA GAAACAGTAT CAGAGGATGA GAAATGAGTG GAAGATGGCC AAAATTGCAC TGATTGTCAT CTTGCTTTAT GTCATTTCCT GGTCACCATA
Rutilu027    -------GTG GGGAAACGCA GAAGGTGTAT GAACGCATGC AGAACGAATG GAAGATGGCG AAAATCGCCC TCCTGGTCAT TTTGCTTTTT GTAATATCCT GGTCGCCCTA
Taenio028    -GTGGAAATA GAGAGTTCCA GAAACAGTAT CAGAGGATGA AAAATGAGTG GAAGATGGCC AAAATTGCAC TGATTGTCAT CTTGTTTTTT GTCATTTCCT GGTCACCATA
Taenio029    ------TGCA GCCGGAAATC CTACGTCTCT CAGTCCATCA AGAATGAGTG GAAGCTGGCA AAAATTGCTT TTGTCATCAT TATTGTGTTT GTCTTGTCCT GGTCTCCATA
Tarsiu030    -AGGATGGCA GCGAGTCCCT GTGGCAGCGG CAGCGGCTAC AAAGTGAGTG GAAGATGGCC AAGATCATGC TACTAGTCAT CCTTCTCTTC GTGCTCTCCT GGGCCCCCTA
Tinamu031    -ATGGAAATA GGGAGTTCCA AAAACAGTAT CAGAGGATGA AAACTGAGTG GAGGATGGCC AAAATTGCCC TGGTTGTCAT CTTGCTTTAC GTCATTTCCT GGTCACCATA
Trache032    ------TATG GTCGTAAATC CTACCTCTCG CATTCCATGA AGAATGAATG GAAGCTGGCA AAAATTGCTT TTGTGTCTTT GTTGTGTTTG TCTTGTCCTG GTCTCCCTAT
Xenopu033    -ATAACAAAA AGGAATCTCA CAAACAGTAT CAGAAAATGA AGAATGAATG GAAAATGGCC AAAATTGCTT TGATTGTTAT CCTCCTTTAT GTTGTTTCAT GGTCACCATA
Bjar_O034    ------TCTA AACGAAAACA GTCAAGCTCA CAGAGCATAA AGAGTGAATG GAAACTGGCA AAAGTTGCCT TCGTAGCCAT TGTGGTTTAT GTTGTATCCT GGTCTCCATA
Cbic_O035    ------ACTA AACGGAAAAA GTCAAGCTCA CAGAGCATAA AGAATGAATG GAAACTGGCA AAGGTTGCCT TCGTAGCTAT TGTGGTTTAC GTTGTATCCT GGTCTCCATA
Cdur_O036    ------TCTA AACGAAAACA GTCAAGCTCA CAGAGCATAA AGAGTGAATG GAAACTGGCA AAAGTTGCCT TCGTAGCCAT TGTGGTTTAT GTTATATCCT GGTCTCCGTA
Dips_O037    ------TCTA AACGGAAAAA GTCAAGCTCA CAGAGCATAA AGAGTGAATG GAAACTAGCA AAAGTTGCCT TTGTAGCCAT TGTGGTTTAC GTTGTATCCT GGTCTCCATA
Eceph_038    ------TCTA AACGGAAAAA GTCAAGCTCA CAGAGCATAA AGAATGAATG GAAACTGGCA AAAGTTGCYT TCGTAGCCAT TGTGGTTTAC GTTGTATCCT GGTCTCCATA
Eund_O039    ------TCTA AACGGAAAAA GTCAAGCTCA CAGAGCATAA AGAATGAATG GAAACTGGCA AAAGTTGCCT TCGTAGCCAT TGTGGTTTAC GTTGTATCCT GGTCTCCATA
Ery_OP040    ------TCTA AACGGAAAAA GTCAAGCTCA CAAAGCATAA AGAATGAATG GAAACTGGCC AAAGTTGCCT TCGTAGCCAT TGTGGTTTAC GTTGTATCCT GGTCTCCATA
Hel_OP041    ------TCTA AACGGAAAAA GTCAAGCTCA CACAGCATAA AGAATGAATG GAAACTGGCA AAAGTTGCCT TCGTAGCCAT TGTGGTTTTC GTTGTATCCT GGTCTCCTGA
Lmil_O042    ------TCTA AACGGAAAAA GTCAAGCTCA CACAGCATAA AGAATGAATG GAAACTGGCC AAAGTTGCCT TCGTAGCCAT TGTGGTTTAC GTTGTATCCT GGTCTCCTGA
Mcor_O043    ------TCTA AACGGAAAAA ATCAAGCTCA CAGAGCATAA AGAATGAATG GAAACTAGCA AAAGTTGCCT TCGTAGCCAT TGTGGTTTAC GTTGTATCCT GGTCTCCATA
Mlem_O044    ------TCTA AACGGAAAAA GTCAAGCTCA CAGAGCCTAA AGAATGAATG GAAACTAGCA AAAGTTGCCT TCGTAGCCAT TGTGGTTTAT GTTGTATCCT GGTCTCCATA
Oguib_045    ------TCTA AACGGAAAAA GTCAAGCTCA CAGAGCATAA AGAATGAATG GAAACTGGCA AAAGTTGCCT TTGTAGCCAT TGTGGTTTAC GTTGTATCCT GGTCTCCATA
Arizon046    ------ACTA AACGGAAAAA ATCAAGCTCA CATAACATAA AGAATGAGTG GAAACTGGCA AAGGTTGCCT TTGTAGCTAT TGTGGTTTAC GTTGTATCCT GGTCTCCGTA
Cemoph047    ------ACTA AACGGAAAAA ATCAAGCTCA CAGAACATAA AGAATGAATG GAAACTGGCA AAGGTTGCCT TCGTAGCTAT TGTGGTTTAC GTTGTATCCT GGTCTCCGTA
Hypsig048    ------TCTA AACGGAAAAA GTCAAGCTCA CAGAGTATAA AGAGTGAATG GAAACTGGCA AAAGTTGCCT TCGTAGCCAT TGTGGTTTAC GTTGTATCCT GGTCTCCTTA
Lampro049    ------ACTA AACGGAAAAA ATCAAGCTCA CAGAACATAA AGAATGAATG GAAACTGGCA AAGGTTGCCT TCGTAGCTAT TGTGGTTTAC GTTGTATCCT GGTCTCCGTA
Pgutat050    ------ACTA AACGGAAAAA ATCAAGCTCA CAGAACATAA AGAATGAATG GAAACTGGCA AAGGTTGCCT TCGTAGCTAT TGTGGTTTAT GTTGTATCCT GGTCTCCGTA
Mastic051    ------ACTA AACGGAAAAA ATCAAGCTCA CAGAACATAA AGAATGAATG GAAACTGGCA AAGGTTGCCT TCGTAGCTAT TGTGGTTTAC GTTGTATCCT GGTCTCCGTA
Ppat_O052    ------TCTA AACGGAAAAA GTCAAGCTCA CAGAGCATAA AGAATGAATG GAAACTGGCA AAAGTTGCCT TCGTAGCCAT TGTGGTTTAC GTTGTATCCT GGTCTCCATA
Smik_O053    ------TCTA AGCGGAAAAA GTCAAGCTCA CAGAGCATAA AGAGTGAATG GAAACTAGCA AAAGTTGCCT TAGTAGCCAT TGTGGTTTAC GTTGTATCCT GGTCTCCTTA
Snew_O054    ------TCTA AACGGAAAAA GTCAAGCTCA CAGAGCATAA AGAGTGAATG GAAACTAGCA AAAGTTGCCT TAGTAGCCAT TGTGGTTTAC GTTGTATCCT GGTCTCCGTA
Tae_OP055    ------TCTA AAYGGAAAAA GTCAAGCTCA CAGAGCATAA AGAATGAATG GAAACTGGCA AAAGTTGCCT TCGTAGCCAT TGTGGTTTAC GTTGTATCCT GGTCTCCGTA
Tham_O056    ------TCTA AACGGAAAAA GTCAAGCTCA CAGAGCATAA AGAATGAATG GAAACTGGCA AAAGTTGCCT TCGTAGCCAT TGTGGTTTAC GTTGTATCCT GGTCTCCTGA
Tdor_O057    ------TCTA AACGGAAAAA GTCAAGCTCA CAGAGCATAA AGAATGAATG GAAACTGGCA AAAGTTGCCT TCGTGGCCAT TGTGGTTTAC GTTGTATCCT GGTCTCCGTA
Anolis058    ------TATA ACCGGAAATC CTCTATTTCA CACTCTATAA AGAGTGAATG GAAGCTTGCA AAAATTGCCT TTGTGGCCAT TGTGGTGTTC GTCTTGTCCT GGTCTCCATA
Pogona059    ------TATA ACCGGAAATC CTCTGTCTCA CAATCTATAA AGAGTGAATG GAGGTTGGCA AAAATTGCCT TTGTGTCCAT TGTTGTGTTT GTCCTATCAT GGTCTCCATA
Protob060    ------TCTA AACGAAAACA GTCAAGCTCA CAGAGCATAA AGAGTGAATG GAAACTGGCA AAAGTTGCCT TCGTAGCCAT TGTGGTTTAT GTTGTATCCT GGTCTCCGTA
Thamno061    ------ACTA AACGGAAAAA GTCAAGCTCA CACAGCATAA AGAATGAATG GAAGCTGGCC AAGGTTGCCT TCGTAGCCAT TGTGGTTTAT GTTGTATCTT GGTCTCCGTA
Alliga062    ------TACA GCCGGAAGTC ATACCTCTCA CATTCCATGA AGAATGAATG GAAATTGGCA AAAATTGCTT TTGTGGTCAT TATTGTGTTT GTCGTGTCCT GGTCTCCATA
Athene063    -ATGGAAATA AGGAGTTCCA GAAACAGTAT CAGAGGATGA AAAATGAGTG GAAGATGGCC AAAATCGCAC TGATTGTCAT CTTGCTTTAT GTCATTTCCT GGTCACCATA
Callor064    ----CCAACA GGGAATCTCA GAAACAACAC CAGAGGATGA AGAACGAGTG GAAAATGGCA AAAATTGCCT TGATAGTCAT CTTGCTTTTT GTTATCTCCT GGTCCCCATA
Chelon065    ---GCTAATA AAGTATCTCA GAGACAGTAT CAGAAGATGA AGAATGAATG GAAAATGGCC AAAATTGCCT TGATTGTCAT CTTACTTTAC ATCATTTCCT GGTCACCATA
Chelon066    ------TATG GTCATAAATC CTACCTCTCG CATTCCATGA AGAATGAATG GAAGCTGGCA AAAATTGCCT TTGTGGTCAT CATTGTGTTT GTCTTTTCTT GGTCTCCATA
```

```
Chryse067     ---GCTAATA AAGTATCCCA GAGACAGTAT CAGAGGATGA AGAATGAATG GAAAATGGCC AAAATTGCCT TGATTGTCAT CTTACTTTAC GTCATTTCCT GGTCACCATA
Danio_068     -------GCG GGGAAACGCA CAAGGTGTAT GAACGCATGC AGAACGAATG GAAGATGGCG AAAGTCGCTC TTGTAGTCAT TGTGCTTTTT ATTATATCCT GGTCGCCCTA
Danio_069     ----TGAGGA AGACCACCCT CATCCAACAG CAGTCCATAA AGACGGAGTG GAAGCTGGCC AAGATTGCGT TTGTGGTCAT CATTGTGTTT GTGCTGTCCT GGTCTCCGTA
Danio_070     -GAGGACCCA GAGATTCTAT CAAAAAGATC CACCGGATGA AAAACGAATG GAAGATGGCA AAGATAGCGC TCATAGTGAT CCTGTTGTAT GTCATCTCCT GGTCTCCATA
Danio_071     --------GA AGTCCAGCTT TGTGAAGCAG CAGTCCATGA GGAGTGAATG GAAACTGGCT AAAATAGCGG CTGTGGTCAT TGTGGTGTAT GTTTTGTCCT GGGCTCCATA
Egrett072     -ATGGAAATA AGGAAATCCA GAAACAGTAT CACAGGATGA AAAAAGAGTG GAAGATGGCC AAAATTGCAC TGATTGTCAT CTTGCTTTAT GTCATTTCCT GGTCACCATA
Empido073     -ACGGAAATA AAGAGTTCCA GAAACAGTAT CGGAGGATGA AAACTGAGTG GAAGATGGCC AAAATTGCAC TGATTGTCAT CTTGCTTTTT GTCATTTCCT GGTCACCATA
Eptatr074     ----AACGTG GTGAGACGAG GCGAGCAGAT AAGAGGCTAA AAGGAGAATG GCGTCTCGCC AAGATCGCTC TCATTATCAT CCTGCTCTTC ATTGTTTCCT GGTCTCCTTA
Eptesi075     -AGGGCCGCA GTGAACTGCC CCGACAGCGG CAGGGGCTGC AGAGTGAGTG GAAAATGGCC AAGATTGTGC TGCTGGTCAT TCTTCTCTTT GTGCTCTCCT GGGCCCCCTA
Erpeto076     ----ACAACA GAGAATCACT AAGGAAGTTC CAGAAAATGA AGAATGAATG GAAAATGGCA AAAATTGCTC TTATTGTCAT TCTCCTCTAT GTTATTTCCT GGTCACCATA
Falco_077     -ATGGAAATA AAGAGTTCCA GAAACAGTAT CGGAGGATGA AAAATGAGTG GAAGATGGCC AAAATTGCAC TGATTGTCAT CTTGCTTTAT GTCATTTCCT GGTCACCATA
Gekko_078     ------TCTG GTCGGAAATC AAACATCTCT CAGTCCATTA AAAGTGAATG GAAGCTGGCA AAGATTGCTT TTGTGGCCAT TGTGGTCTAT GTCTTGTCCT GGTCTCCGTA
Hippog080     ACCAGCGGTC GTGACTCGGT GAAGAGTTAC CATCGCTTGC AGAACGAGTG GAAGATGGCG AAGATCGCCC TGATCGTCAT CCTGCTCTAC GTCATCTCAT GGTCGCCGTA
Hippog081     -TGGGAAACA GAGACTCAGT GAAGAGGTTC AACAAGATGA AGACTGAATG GAAGATGGCG AAGATTGCGC TCATCGTCAT CCTGCTGTTT GTCATCTCCT GGGCCCCTTA
Hippog082     ----TGAGGA AGTCCACCCT GATCCAGCAG CAGTCCATCA AGACTGAATG GAAGCTGGCC AAGATTGCCT TTGTGGTCAT CATAGTGTTT GTGCTTTCCT GGTCGCCCTA
Hippog083     ----TGAGGA AATCCACCCT CATCCAGCAG AAGTCCATCA AAAGCGAGTG GAAGTTGGCG AAGATCGCCT TCGTCGTCAT CGTGGTTTAT GTCCTCTCCT GGTCACCATA
Ictalu084     GGAATCACCA AAGACTCAAT CAAAAAGTTC CATCGATTGA AGAGTGAATG GAAGATGGCA AAGGTGGCTC TCATAGTCAT TCTGTTGTAC GTGATCTCCT GGTCTCCATA
Ictalu085     -------CCG GTGAAACACC TAAGGCGCTC GAATGCATCA AGACTGAATG GAAAATGGCT AAAGTGGCCC TCTTGGTCAT CTTACTATTT GTGATTTCCT GGTCACCTTA
Larimi086     GGCAGCAGCC GCGACTCGGT GAAGAATTTC CAGCGGCTGC AGAACGAGTG GAAGATGGCG AAGATCGCTC TGATCGTCAT CCTGCTTTAC GTCATCTCCT GGTCGCCGTA
Lethen087     -ATGATAGTA AGGAATCACA GAAGCAGCAG CAACGGATGA ACGCCGAGTG GAAGCTTGCG AAGATTGCCC TCATTGTCAT CTTGCTCTAT GTGGTGTCCT GGTCTCCGTA
Mastac088     AGCAGCTGTC GGGACTCTGT GAAAAGGTTC CACCGACTGC AGAACGAGTG GAAGATGGCG AAGATCGCTC TCATCGTCAT CCTGCTGTAC GTCGTCTCCT GGTCGCCGTA
Meleag089     ------TGCA GCCGCAAATC CTTCCTCTCT CAGTCCATGA AGAATGAATG GAAACTGGCA AAAATTGCAT TTGTGGTCAT CATTGTGTAT GTCTTATCCT GGTCTCCATA
Meleag090     -ATGGAAACA AAGAGCTCCA GAAACAGTAT CACAGGATGA AGAATGAGTG GAAGTTGGCC AAGATTGCAC TGATCGTCAT CTTGCTTTAT GTCATTTCCT GGTCACCATA
Notech091     ------TCTA AACGGAAAAA GTCAAGCTCA CAGAGCATAA AGAATGAATG GAAACTAGCA AAAGTTGCCT TCGTAGCCAT TGTGGTTTAT GTTGTATCCT GGTCTCCGTA
Opisth092     -ACGGAAATA GAGAGTTCCA GAAACAGTAT CAGAGGATGA AAAATGAGTG GAAGATGGCC AAAATTGCAC TGATTGTCAT CTTGCTTTAT GTCATTTCCT GGTCACCATA
Pelodi093     ---GGTAATA AAGTATCCCA GAGACAGTAT CAGAAGATGA AGAATGAATG GAAAATGGCC AAAATTGCAT TGATTGTCAT CTTGCTTTTC GTCATTTCCT GGTCACCATA
Phyllo094     -AAGGCCACA GTGAATTGCC CTGGCAACAG CAGCGGCTGC AGAGCGAGTG GAAAACGGCC AAGATTGTCT TGCTAGTCAT CCTTCTCTTC GTCCTGTCCT GGGCACCCTA
Physet095     -AGGGTGGTG GTGAGTGCCC CTGGCAACGG CAGCGTCTGC AGAACGAGTG GAAAATGGCC AAGATCGAGC TGTTGGTCAT CCTTCTCTTT GTGCTCTCCT GGGCCCCCTA
Pipra_096     -ATGGAAATA AAGAGTTCCA GAAACAGTAT AAGAGGATGA AAAATGAGTG GAAGATGGCC AAAATTGCAC TGATTGTCAT CTTGCTTTTT GTCATTTCCT GGTCACCATA
Podarc097     ------TATA ACCGGAAGTC GAATGTTTCG CAGTCAGTGA AGAGTGAATG GAAGTTAGCA AAAATTGCCT TTGTGGCTAT TGTGGTGTTT GTTCTGTCCT GGTCCCCATA
Podarc098     ----GTACAA AAGAATCTCA AAGGCTGTCC CAGAGGATGA CGAATGAATG GAAAATGGCC AAAGTAGCCC TGGTTATCAT CTTGCTTTAT GTCCTCTCCT GGTCTCCATA
Pseudo099     ------TCTA AACGGAAAAA GTCAAGCTCG CAGAGCATAA AGAATGAATG GAAACTAGCA AAAGTTGCCT TCGTAGCCAT TGTGGTTTAT GTTGTATCCT GGTCTCCGTA
Python100     ------TATA AACGGAAACA TTCAGCCTCA CAGAACATAA AAAGTGAATG GAAACTGGCA AAAATTGCCT TTGTAGCCAT TGTGGTTTAT GTTGTATCCT GGTCTCCATA
Salmo_101     -GGAGCACCA GAGACTCAAT TAAGAGTTTC CGTCGGCTGA AGAATGAGTG GAAGATGGCT AAGATTGCTC TGATAGTCAT CCTCCTCTAT GTAATCTCTT GGTCCCCATA
Salmo_102     ----TGAGGA AATGTACGCT GATCCAGCAG AAGAGCATCA GGAATGAGTG GAAGCTGGCC AAAATCGCCT TTGTGGTCAT TGTTGTGTAT GTGCTGTCCT GGTCGCCGTA
Salmo_103     ----TGAGGA AGTCCACCCT CATCCAGCAG CAGTCCATCA AGACAGAGTG GAAGCTGGCC AAGATTGCCT TTGTGGTCAT CATCGTCTAT GTGATGTCCT GGTCTCCCTA
Salmo_104     ----TAAGGA AGTCCACCCT GATCCAGCAG CAGTCCATCA AGACTGAGTG GAAGCTGGCC AAGATTGCCT TTGTAGTCAT CATCGTGTAT GTGATGTCCT GGTCTCCCTA
Stegas105     ---------- GCGACTCGGT GAAGAGTTTC CACCGGCTGC AGAACGAGTG GAAGATGGCG AAGATCGCTC TGATCGTCAT CCTGCTCTAC GTCATCTCCT GGTCGCCGTA
Terrap106     ---GCTAATA AAGTATCCCA GAGACAGTAT CAGAGGATGA AGAATGAATG GAAAATGGCC AAAATTGCCT TGATTGTCAT CTTACTTTAC GTCATTTCCT GGGCACCATA
Xenopu107     -ATGACAATA AGGAATCTCA TAAACAGTAT CAGAAAATGA AGAATGAATG GAAAATGGCA AAAATTGCTC TGATTGTAAT CCTCCTTTAT GTTGTTTCAT GGTCACCATA


              ....|....| ....|....| ....|....| ....|....| ....|....| ....|....| ....|....| ....|....| ....|....| ....|....| ....|....|
                      780        790        800        810        820        830        840        850        860        870        880
Felis000      cTcCaTtGtG gCcCtGaTgG cCtTtGcTgG gTaCgCgCaT gTgCtGaCaC cCtAcAtGaA cTcGgTgCcA gCtGtCaTcG cCaAgGcCtC tGcCaTcCaC aAcCcCaTcA
Gallus001     TGCTTGTGTC ACCTTGATTG CCTGGGCAGG CCGAGGAAAC ACTCTAACGC CGTATTCCAA ATCTGTGCCA GCTGTTATTG CTAAAGCCTC TGCAATCTAC AACCCCATCA
Gallus002     CTCTGTTGTT GCCCTGGTAG CTTTTGCTGG GTATTCTCAT GTACTAACAC CTTTCATGAA TTCAGTACCA GCTGTGATTG CCAAAGCTTC TGCCATCCAT AACCCCATTA
Gadus003      TGCCTGTGTG ACGATGATTT CCTGGTCAGG GCATGCCAAC ATCCTGTCGC CGTACTCCAA AACTGTCCCA GCTGTTATAG CGAAAGCATC TACCATTTAC AACCCATTCA
Gadus_004     CGCGTGCGTC ACACTCATCG CCTGGGCGGG GTACGGGAGC CACCTCAGCC CCTACTCCAA AGCGGTGCCG GCTGTCATCG CCAAAGCCTC CGCCATCTAC AACCCCTTCA
Sminth005     TTCAACAGTG GCCCTTGTGG CCTTTGCTGG GTATTCCCAC GTCCTGACTC CCTATATGAA CTCGGTGCCA GCCATCATTG CCAAGGCCTC TGCCATCCAC AATCCGATCA
Homo006  C    TCCGCTGTGG CCCTGGTGGC CTTTGCTGGG TACGCACACG TCCTGACACC CTACATGAGC TCGGTGCCAG CCGTCATCGC CAAGGCCTCT GCAATCCACA ACCCCATCAT
Mus007  CT    CCACTGTGGC TCTGGTGGCC TTTGCTGGAT ACTCGCACAT CCTGACGCCC TACATGAGCT CGGTGCCAGC CGTCATCGCC AAGGCTTCTG CCATCCACAA TCCCATTATC
Spalax008     CTCCACAGTG GCCCTGGTGG CCTTTGCTGG GTACTCGCAC ATTCTGACGC CCTACATGAA CTCAGTGCCA GCTGTCATCG CCAAGGCTTC TGCCATCCAC AATCCCATCA
Monode009     TTCCACCGTG GCCCTGGTGG CCTTTGCTGG GTATTCCCAC ATCCTGACTC CCTACATGAA CTCTGTGCCA GCCATCATCG CCAAAGCCTC TGCCATCCAC AATCCCATCA
Rattus010     CTCCACTGTG GCCCTGGTGG GCTTTGCTGG GTACTCGCAC ATCCTGACGC CCTACATGAG CTCGGTGCCA GCCGTCATTG CCAAGGCCTC GGCCATCCAC AATCCTATCA
Podarc011     TGCTTGTGTC ACTCTGATTG CGTGGGCAGG CTACGCCAAA ACCTTAAACC CATATTCTAA ATCTGTGCCA GCTGTTATTG CCAAAGCTTC TGCGATCTAC AATCCCATAA
Trache012     TTCTGTTGTA GCTCTGGTGG CTTTTGCTGG GTATTCCCAT CTCCTGACAC CCTTTATGAA CACCGTACCA GCTGTGATTG CCAAAGCTTC TGCCATCCAC AACCCCATCA
Xenopu013     TGCTTGCGTC ACACTGATTG CTTGGGCTGG GCATGGTAAA TCTTTAACTC CCTATTCCAA GACAGTCCCA GCAGTCATTG CCAAGGCATC TGCGATCTAC AATCCAATTA
Danio014      CTCAACAGTG GCACTGACAG CCTTTGCTGG GTATTCTGAT TTTCTCACAC CATATATGAA TTCAGTGCCA GCTGTGATTG CTAAAGCTTC AGCGATCCAC AATCCCATAA
Danio_015     TTCTGTGGTG GCTCTCACCG CTACGGCTGG TTATTCCCAT TTCCTGACTC CCTATATGAA TTCAGTACCT GCTGTGATTG CCAAGGCTTC AGCCATCCAT AATCCAATCA
Anolis016     TTCTGTGGTA GCTTTGGTGG CTTTTGCAGG ATACTCTCAT CTCCTTACAC CATACATGAA CTCAGTCCCT GCAGTGATTG CCAAGGCTTC TGTTATCCAC AACCCAATCA
Bos017  CT    CCACTGTCGC CCTGATGGGC TTTGCTGGGT ACGCACATAT CCTGACGCCC TACATGAACT CGGTGCCAGC TGTCATTGCC AAGGCCTCTG CCATCTACAA CCCCATCATT
Callor018     TGCATGTGTG ACATTAGTTG GATGGGCAGG GCATGGCGAT ATCCTGACTC CCTACTCGAA AACCGTTCCT GCCGTATTAG CAAAAGCTTC TGCCATCTAC AATCCCATCA
Canis019      CTCTGCCGTG GCCCTGACGG CTTTCGCTGG GTATTCGCAC GTCCTGACGC CCTACATGAA CTCCGTGCCA GCCGTCATCG CCAAGGCCTC CGCCATCCAC AACCCCATCA
Cynogl020     CTCCGCCGTC GCCCTCACCG CCTTCGCAGG ATATGCAGAC ATGTTGACGC CGTACATGAA CTCCGTCCCG GCCGTCATTG CCAAGGCCTC AGCGATCCAC AACCCCATCA
Dipodo021     TTCCACTGTG GCCCTGGTGG CCTTTGCTGG GTATGCACAC ATCCTGACGC CCTACATGAA CTCAGTGCCA GCCGTCATCG CCAAGGCTTC TTCCATCCAC AACCCCATCG
Esox022  A    TCCGCTGTGG CCCTGACATC CTTTGCAGGG TACGCTGATT TTCTAACGCC CTACATGAAC TCAGTGCCTG CAGTGATCGC CAAGGCTTCA GCCATTCACA ACCCCATCAT
Haliae023     CTCTGTTGTT GCTCTGGTAG CTTTTGCTGG GTATTCCCAG GTCCTAACAC CCTTCATGAA CTCCATACCA GCTGTGATTG CCAAAGCGTC TGTCATCCAT AACCCCATCA
Macaca024     TTCTGCTGTG GCCCTGGTGG CCTTTGCTGG GTACGCACAT GTCCTGACAC CCTACATGAG CTCGGTGCCA GCCGTCATCG CCAAGGCCTC TGCAATCCAC AACCCCATCA
Nototh025     CTCGTGTGCT GCCCTCACTG CATTTGCTGG GCACGCTGAC ATGTTGACTC CGTACATGCA CTCTGTTCCT GCTGTGATCG CCAAGGCATC TGCAATCCAC AACCCTATCA
Pygosc026     CTCTGTTGTT GCTCTGGTAG CTTTTGCTGG GTATTCCCAC GTCCTCACAC CCCTCATGAA CTCCATACCA GCTGTGATTG CCAAAGCTTC TGTCATCCAT AACCCCATCA
Rutilu027     CTCTGTGGTG GCCCTCACCG CCACAGCTGG CTATTCCCAT CTCCTGACCC CCTACATGAA TTCAGTACCC GCTGTGATTG CCAAGGCCTC AGCCATCCAT AATCCCATCA
Taenio028     CTCTGTTGTT GCTCTGGTAG CTTTTGCTGG GTATTCCCAC GTCCTAACAC CTTTCATGAA CTCCATACCA GCTGTGATTG CCAAAGCTTC TGTCATCCAT AACCCCATCA
Taenio029     TGCTTGTGTC ACCCTGATTG CCTGGGCAGG TCGAGGCAAC AGCCTAACAC CATATTCCAA ATCTGTGCCA GCAGTTATTG CCAAAGCCTC TGCAATCTAC AACC------
Tarsiu030     CTCTGCTGTG GCTCTGGTGG CCTTTGCAGG GTATGCACAT GTCCTGACGC CCTACATGAA CTCGGTGCCA GCTGTCATCG CCAAGGCCTC TGCCATCCAT AACCCTATCA
Tinamu031     CTCTGTTGTT GCTCTGGTAG CTTTTGCTGG GTATTCCCAC ATCCTCACAC CCTACATGAA CTCCATACCA GCTGTGATTG CCAAAGCTTC TGCCATCCAC AACCCCATCA
Trache032     GCTT------ ---------- ---------- ---------- ---------- ---------- ---------- ---------- ---------- ---------- ----------
Xenopu033     TTCTACAGTG GCCCTACTGG CCTTTGCAGG ATATGCAAGC ATCTTAACAC CATACATGAA CTCTGTGCCT GCTGTGATTG CAAAAGCTTC TGCCATTCAC AATCCTATCA
Bjar_O034     TGCTTGTGTC ACCCTGATTG CATGGGCTGG TTATGCCAGA ATCTTAACTC CGTATTCTAA ATCTGTGCCT GCTATTATTG CTAAAGCTTC AGCAATTCAC AATCCTATAA
Cbic_O035     TGCTTGTGTC ACTCTGATTG CATGGGCTGG TTATGCCAAR ATCTTAACTC CATATTCTAA ATCTGTGCCT GCTGTTATTG CCAAAGCTTC AGCAATTCAC AATCCCATAA
Cdur_O036     TGCTTGTGTC ACCCTGATTG CATGGGCTGG TTATGCCAGG ATCTTAACTC CATATTCTAA ATCTGTGCCT GCTGTTATTG CTAAAGCTTC AGCAATTCAC AATCCTATAA
Dips_O037     TGCTTGTGTC ACTCTGATTG CATGGGCTGG TTATGCCAGA ATCTTAACTC CATATTCTAA GTCTTTGCCT GCTGTTATTG CCAAAGCTTC AGCAATTCAC AATCCTATAA
Eceph_038     TGCTTGTGTC ACTCTGATTG CATGGGCTGG TTATGCCAAA GTCTTAACTC CATATTCTAA GTCTGTGCGT GCTGTTATTG CCAAAGCTTC AGCAATTCAC AATCCTATAA
Eund_O039     TGCTTGTGTC ACTCTGATTG CATGGGCTGG TTATGCCAAA GTCTTAACTC CATATTCTAA GTCTGTGCCT GCTGTTATTG CCAAAGCTTC AGCAATTCAC AATCCTATAA
Ery_OP040     TGCTTGTGTC ACTCTGATTG CATGGGCTGG TTATTCCAGA GTCTTAACAC CATATTCCAA GTCTGTGCCT GCTGTTATTG CCAAAGCTTC AGCAATTCAC AATCCTATAA
Hel_OP041     TGCTTGTGTC ACTCTGATTG CATGGGCTGG TTATGCCAGA GTCTTAAATC CATATTCAAA GTCTGTGCCT GCTGTTATTG CCAAAGCTTC AGCAATTCAC AATCCTATAA
Lmil_O042     TGCTTGCGTC ACTCTGATTG CATGGGCTGG TTATTCCAGA GTCTTAACCC CATATTCCAA GTCTGTGCCT GCTGTTATCG CCAAAGCTTC AGCAATTCAC AACCCTATCA
Mcor_O043     TGCTTGTGTC ACCCTGATTG CATGGGCTGG TTATGCCAGG GTCTTAACTC CATATTCTAA GTCTGTGCCT GCTGTTATTG CCAAAGCTTC AGCAATTCAC AATCCTATAA
Mlem_O044     TGCTTGTGTC ACCCTGATTG CATGGGCTGG TTATGCCAGG GTCTTAACTC CATATTCTAA GTCTGTGCCT GCTGTTATTG CCAAAGCTTC AGCAATTCAC AATCCTATAA
Oguib_045     TGCTTGTGTC ACTCTGATTG CGTGGGCTGG TTATGCCAGA GTCTTAAATC CATATTCTAA GTCTGTGCCT GCTGTTATTG CCAAAGCTTC AGCAATTCAC AATCCTATAA
Arizon046     TGCTTGTGTC ACTCTAATTG CATGGGCTGG TTATGCCAAG ATCTTAACTC CATATTCTAA ATCTGTGCCT GCTGTTATTG CCAAAGCTTC AGCAATTCAC GGGCCCATAA
Cemoph047     TGCTTGTGTC ACTCTGATTG CATGGGCTGG TTATGCCAAG ATCTTAACTC CATATTCTAA ATCTGTGCCT GCTGTTATTG CCAAAGCTTC AGCAATTCAC AATCCCATAA
Hypsig048     TGCTTGTGTC ACTCTGATTG CATGGGCTGG TTATGCCAAA GTCTTAACTC CATATTCTAA GTCTGTGCCT GCTGTTATTG CCAAAGCTTC AGCAATTCAC AATCCTATAA
Lampro049     TGCTTGTGTC ACTCTGATTG CATGGGCTGG TTATGCCAAG ATCTTAACTC CATATTCTAA ATCTGTGCCT GCTGTTATTG CCAAAGCTTC AGCAATTCAC AATCCCATAA
Pgutat050     TGCTTGTGTC ACTCTGATTG CATGGGCTGG TTATGCCAAG ATCTTAACTC CATATTCTAA ATCTGTGCCT GCTGTTATTG CCAAAGCTTC AGCAATTCAC AATCCCATAA
Mastic051     TGCTTGTGTC ACTCTGATTG CATGGGCTGG TTATGCCAAG ATCTTAACTC CATATTCTAA ATCTGTGCCT GCTGTTATTG CCAAAGCTTC AGCAATTCAC AATCCCATAA
Ppat_O052     TGCTTGTGTC ACTCTGATTG CATGGGCTGG TTATGCCAGA GTCTTAACTC CATATTCTAA GTCTGTGCCT GCTGTTATTG CCAAAGCTTC AGCAATTCAC AATCCTATAA
Smik_O053     TGCTTGTGTC ACTCTGATTG CGTGGGCTGG TTATGCCAGA ATCTTAACTC CATATTCTAA GTCTTTGCCT GCTGTTATTG CCAAAGCTTC AGCAATTCAC AATCCTATAA
Snew_O054     TGCTTGTGTC ACTCTGATTG CATGGGCTGG TTATGCCAGA GTCTTAACTC CATATTCTAA GTCTCTGCCT GCTGTTATTG CCAAAGCTTC AGCAATTCAC AATCCTATAA
Tae_OP055     TGCTTGTGTC ACTCTGATTG CATGGGCTGG TTATGCCAGA GTCTTAACTC CATATTCTAA GTCTGTGCCT GCTGTTATTG CCAAAGCTTC AGCAATTCAC AATCCTATAA
Tham_O056     TGCTTGTGTC ACTCTGATTG CATGGGCTGG TTATGCCAGA GTCTTAACTC CATATTCTAA GTCTGTGCCT GCTGTTATTG CCAAAGCTTC AGCAATTCAC AATCCTATAA
Tdor_O057     TGCTTGTGTC ACTCTGATTG CATGGGCTGG TTATGCCAGA GTCTTAAATC CATATTCTAA GTCTGTGCCT GCTGTTATCG CCAAAGCTTC AGCAATTCAC AATCCTATAA
Anolis058     TGCTTGTGTT ACTCTAATTT CATGGGCAGG TTACGCCAGG ACCTTAACTC CGTATTCTAA ATCTGTGCCT GCTGTTATTG CCAAAGCTTC TGCAATCTAC AATCCCATAA
Pogona059     TGCTTGTGTT ACTCTCATTG CATGGGCTGG CTATGCCAGG ACTTTAACTC CATATTCTAA ATCTGTGCCT GCTGTTATCG CCAAAGCTTC TGCAATCTAC AATCCCATAA
Protob060     CGCTTGTGTC ACCCTGATTG CATGGGCTGG TTATGCCAGG GTCTTAACTC CGTATTCTAA ATCTGTGCCT GCTGTTATTG CCAAAGCTTC AGCAATTCAC AATCCTATAA
Thamno061     TGCTTGTGTC ACTCTGATTG CATGGGCCGG TTATGCCAGA ATCTTAACTC CATATTCTAA GTCTGTGCCT GCTGTTATTG CCAAAGCTTC AGCAATTCAC AATCCTATAA
Alliga062     TGCTTGTGTC ACTTTGATTG GATGGGCAGG CCAGGGTGAC ATCCTAACAC CATATTCTAA ATCTGTGCCA GCTATTATTG CCAAAGCTTC TACAATCTAC AACCCGATCA
Athene063     CTCTGTTGTT GCTCTGGTGG CTTTTGCTGG GTATTCCCAC GTCCTAACTC CCTTCATGAA CTCCATACCA GCTGTGATTG CCAAAGCTTC TGTCATCCAT AACCCCATCA
Callor064     CTCCACAGTG GCTCTCACTG CATTTGCTGG ATACTCCAGT ATTCTTACTC CCTACATGAA CTCTATCCCA GCCGTCATTG CCAAAGCCTC GGCCATCCAC AACCCCATCA
Chelon065     TTCTATTGTA GCTCTGGTGG CTTTTGCTGG GTATTCCCAT CTCCTGACAC CCTTTATGAA CACCGTACCA GCTGTGATTG CCAAAGCTTC TGCCATTCAC AACCCCATCA
Chelon066     TGCTTGTGTC ACAATGATTG CATGGGCGGG CCATGGCAAC ACTCTAACTC CATATTCTAA ATCTGTGCCA GCTGTTATCG CCAAAGCTTC TGCAATCTAC AATCCTATCA
Chryse067     TTCTGTTGTA GCTCTGGTGG CTTTTGCTGG GTATTCCCAT CTCCTGACAC CCTTTATGAA CACCGTACCA GCTGTGATTG CCAAAGCTTC TGCCATCCAC AACCCCATCA
Danio_068     TTCTGTGGTG GCTCTCACCG CTACGGCTGG TTATTCCCAT TTCCTGACTC CCTATATGAA CTCAGTACCT GCTGTGATTG CCAAGGCTTC AGCCATCCAT AATCCAATCA
Danio_069     CGCCTGCGTC ACACTCATCG CCTGGGCTGG ATACAGCCAC GTTCTTTCTC CATATTCCAA GGCAGTTCCT GCTGTGATTG CCAAGGCATC AGCCATATAC AATCCCTTCA
Danio_070     CTCCTGTGTG GCTCTCACTG CTTTTGCTGG ATACGCAGAC ATGCTGACAC CCTACATGAA CTCTGTGCCC GCTGTTATTG CCAAAGCATC TGCCATCCAC AACCCCATCA
Danio_071     TGCCTGTGTG ACCCTCGTGG CCTGGGCAGG GCATCAAGAC GTTCTCACAC CCTACTCAAA AACACTGCCA GCCGTCCTCG CCAAGTCATC AGCCATCTAC AACCCCTTTA
Egrett072     CTCTGTTGTT GCTCTGGTAG CTTTTGCTGG GTACTCCCAC GTCCTAACAC CCTTCATGAA CTCCATACCA GCTGTGATTG CCAAAGCTTC TGTCATCCAT AACCCCATCA
Empido073     CTCTGTTGTT GCTCTGGTAG CTTTTTCTGG GTATTCCCAT GCCCTAACAC CCTACATGAA CTCCATACCA GCTGTGATTG CCAAAGCTTC TGTCATCCAT AACCCCATCG
Eptatr074     CTCGTGTGTG GCACTCATTG CCTGGGCCGG TTATGCTGAA TTGCTGACTC CATATACAAA CTCTATTCCT GCCATCATTG CCAAAGCATC AGCGATTCAT AACCCCATCG
Eptesi075     CTCCACCGTG GCCCTGATGG CCTTTGCTGG GTACGCTCAC GTCCTGACGC CCTACATGAA CTCAGTGCCA GCCATCATCG CCAAGGCCTC TGCCATCCAC AACCCCATCA
Erpeto076     CTCCACAGTG GCCCTTACTG CCTTTGCTGG ATACTCTGAC TTGTTGACAC CTTACATGAA TTCTGTGCCT GCAGTGATAG CCAAGGCTTC AGCCATCCAC AATCCCATTA
Falco_077     CTCTGTTGTT GCTCTGATAG CTTTTGCTGG GTATTCCCAC GTTCTAACAC CCTTCATGAA CTCCATACCA GCTGTGATTG CCAAAGCTTC TGTCATCCAT AACCCCATCA
Gekko_078     TGCTTGCGTT ACTCTCATTG CATGGGCAGG CCATGCCAGC TTCCTGACTC CCTATTCTAA ATCTGTGCCA GCTGTTATTG CCAAAGCTTC TGCGATCTAC AATCCCATCA
```

```
Hippog080    CTCCGCCGCT GCCCTCACCG CCTTCGCCGG ATACGCCGAC ATGTTGACTC CCTACATGAA CTCTGTGCCC GCCGTCATTG CCAAGGCCTC CGCCATCCAC AACCCCATCA
Hippog081    CTCCTGTGTG GCCCTCACTG CATTCGCTGG GTACGCTGAC ATGTTGACTC CCTACATGAA CTCTGTTCCT GCTGTGATCG CCAAAGCATC TGCCATCCAC AACCCCATTA
Hippog082    TGCATGCGTC ACCCTCATCG CCTGGGCTGG ATATGGACAT GTCCTCAATC CGTACTCCAA AGCTGTCCCT GCTGTTATAG CCAAGGCATC AGCCATCTAC AACCCGTTTA
Hippog083    TGCCTGCGTC ACACTGATTT CCTGGGCAGG GCACGCCAAC ATCTTGTCGC CATACTCCAA GGCCGTCCCT GCAATCATAG CAAAAGCCTC GGCCATCTAC AATCCCTTCA
Ictalu084    TTCCTGTGTG GCTCTGACGG CATTTGCAGG TTACGCTGAC TTGCTCACCC CGTACATGAA CTCTGTTCCT GCTGTGATTG CCAAGGCTTC AGCTATCCAC AACCCCATCA
Ictalu085    CTCCGTGGTG GCTCTCACAG CCACAGCTGG CTATGCTCAT GTCATCACTC CTTATATGAA CTCCATCCCT GCAGTGATCG CCAAGGCTTC TGCCATCCAC AACCCCATAA
Larimi086    CTCAACCGTC GCTCTCACCG CCTTCGCCGG GTACTCAGAC ATGTTGACTC CATACATGAA CTCTGTGCCC GCCATCATCG CCAAAGCCTC CGCCATCCAC AACCCCATCA
Lethen087    CTCGTGTGTG GCGCTTGTGG CCTGGGCTGG TTACGCTGAC ATGCTCACGC CCTACATGAA CTCCGTGCCT GCCATCATCG CCAAGGCGTC GGCCATCCAC AACCCCATAG
Mastac088    CTCCGTCGTC GCCCTCACCG CCTTCGCCGG GTACGCAGAC ATGTTGACCC CCTACATGAA CTCTGTGCCC GCCATCATCG CCAAGGCCTC GGCCATCCAC AACCCCATCA
Meleag089    CACTTGTGTC ACTTTGATTG CCTGGGCAGG TCGAGGAAAC ACTCTGACGC CGTATTCCAA ATCTGTGCCA GCTGTTATTG CTAAAGCCTC TGCAATCTAC AACCCCATCA
Meleag090    CTCTGTTGTT GCCCTGGTAG CTTTTGCTGG GTATTCTCAT GTTCTAACAC CCTTCATGAA TTCAATACCA GCTGTGATTG CCAAAGCTTC TGCCATCCAT AACCCCATTA
Notech091    TGCTTGTGTC ACCCTGATTG CATGGGCTGG TTATGCCAGG GTCTTAACTC CATATTCTAA GTCTGTGCCT GCTGTTATTG CCAAAGCTTC AGCAATTCAC AATCCTATAA
Opisth092    CTCTGTTGTT GCTCTGGTAG CTTTTGCTGG GTACTCCCAC GTCCTAACAC CCTTCATGAA CTCCATACCA GCTGTGATTG CCAAAGCTTC TGTCATCCAT AACCCCATAA
Pelodi093    TTCTGCTGTA GCTCTGGTGG CTTTTGCTGG GTATTCCCAT CTCCTGACAC CATTTATGAA CACCATACCA GCTGTGATCG CCAAAGCTTC TGCTATTCAC AACCCCATCA
Phyllo094    CTCCACTGTG GCCTTGATGG CCTTTGCTGG GTATGCGCAT GTCCTGACGC CCTACATGAA CTCAGTGCCA GCTGTTATCG CCAAGGCCTC TGCCATCCAC AACCCCATCA
Physet095    CTCCACCGTG GCCCTGATGG GCTTTGCTGG GTACGCGCAT GTCCTGACGC CCTACATGAC CTCCGTGCCG GCTGTCATCG CCAAGGCCTC TGCCATCTAC AACCCCATCA
Pipra_096    CTCTGTTGTT GCTCTGGTAG CTTTTGCTGG GTATTCCCAT GCCCTAACAC CCTTCATGAA CTCCATACCA GCTGTGATTG CCAAAGCTTC TGTCATCCAT AACCCCATCA
Podarc097    TGCTTGTGTC ACTTTGATTG CGTGGGCAGG CTATGCCAAG ACCTTAAACC CATATTCTAA ATCTGTGCCA GCTGTTATTG CCAAAGCTTC TGCGATCTAC AATCCCATAA
Podarc098    TTCGATGGTG GCTTTGCTGG CTTTTGCAGG GTACTCCCAT GTGCTCACTC CCTTAATGAA CACAATACCT GCAGTGATTG CCAAAGCTTC ATCCATCTAC AACCCAATCG
Pseudo099    TGCTTGTGTC ACCCTGATTG CATGGGCTGG TTATGCCAGG GTCTTAACTC CATATTCTAA GTCTGTGCCT GCTGTTATTG CCAAAGCTTC AGCAATTCAC AATCCTATAA
Python100    TGCTTGTGTT ACCTTGATTG CCTGGGCTGG CTATGCCAGG ATCTTAACTC CATATTCTAA ATCTGTGCCT GCTATTATTG CCAAAGCTTC TGCAATTTAC AATCCTATAA
Salmo_101    CTCTGTTGTG GCCCTGACTG CATTTGCAGG GTATGCAGAT TTTCTGACCC CATACATGAA CTCAGTTCCT GCTGTGATCG CCAAGGCTTC AGCCATTCAC AACCCCATTA
Salmo_102    TGCCACCGTC ACCATGATCT CCTGGGCAGG ACACGCCAAC ATTCTGTCAC CGTACTCCAA AGCCGTACCT GCAGTTATAG CAAAAGCCTC TACCATCTAC AACCCTTTTA
Salmo_103    TGCCTGCGTC ACCCTCATCG CCTGGGCTGG CTATGGACGT GCCCTCAGCC CCTACTCCAA GGCCGTCCCT GCTGTCATCG CCAAAGCCTC AGCCATCTAT AACCCTTTCA
Salmo_104    CGCCTGCGTC ACCCTCATCG CCTGGGCTGG CTATGGAAGT ACCCTCAGTC CCTACTCCAA GGCAGTCCCT GCTGTCATAG CCAAAGCCTC AGCCATCTAT AACCCCTTCA
Stegas105    CTCCACCGTC GCCCTCACCG CCTTCGCCGG CTACGCAGAC ATGTTGACTC CCTACATGAA CTCTGTGCCC GCCGTCATCG CCAAGGCCTC GGCCATCCAC AACCCCATCA
Terrap106    TTCTGTTGTA GCTCTGGTGG CTTTTGCTGG GTATTCCCAT CTCCTGACAC CCTTTATGAA CACCGTACCA GCTGTGATTG CCAAAGCTTC TGCCATCCAC AACCCCATCA
Xenopu107    TTCTACAGTG GCCCTTGTGG CCTTTGCAGG ATATGCAAGT GTCTTAACAC CATACATGAA CTCTGTGCCG GCTGTGATTG CAAAAGCTTC TGCCATTCAC AATCCTATCA


             ....|....| ....|....| ....|.
                     890        900
Felis000     tTtAtGcCaT cAcCcAcCcC aAgTaC
Gallus001    TATATGCAAT AATTCACCCG AGATAC
Gallus002    TTTATGCCAT CACTCACCCT AAATAT
Gadus003     TCTACGCCAT CATACATCAG AAGTAC
Gadus_004    TCTACGCCAT CATCCACTCT AAATAC
Sminth005    TTTATGCCAT CAGCCACCCC AAGTAC
Homo006  C   TTACGCCATC ACCCACCCCA AGTAC.
Mus007  CT   TACGCCATCA CTCACCCCAA GTAC..
Spalax008    TTTATGCCAT CACTCACCCC AAGTAC
Monode009    TTTATGCTAT CAGCCACCCC AAGTAC
Rattus010    TCTATGCCAT CACTCACCCC AAGTAC
Podarc011    TCTATGCAAT CATTCACCCA AGATAC
Trache012    TTTATGCCAT AACTCATCCC AAATAC
Xenopu013    TCTATGGAAT AATACACCCA AAATAC
Danio014     TCTACGCCAT CACACACCCC AAATAC
Danio_015    TCTACGCCAT TACGCATCCA AAGTAT
Anolis016    TTTATGCCAT TGTTCATCCC AAATAC
Bos017  CT   TACGCCATCA CCCACCCCAA GTAC..
Callor018    TCTACGCAAT AATTCACCCC AACTAC
Canis019     TTTACGCCAT CACGCACCCC AAGTAC
Cynogl020    TCTACGCCAT CACACACCCC AAGTAC
Dipodo021    TTTATGCCAT CACCCACCCC AAGTAC
Esox022  A   ATACGCCATC ACCCACCCCA AATAC.
Haliae023    TTTATGCCAT CACTCACCCC AAATAC
Macaca024    TTTACGCCAT CACCCACCCC AAGTAC
Nototh025    TATATGCCAT AACACATCCC AAATAC
Pygosc026    TTTATGCTAT CACTCACCCC AAATAC
Rutilu027    TCTACGCAAT TACACATCCA AAGTAT
Taenio028    TTTATGCTAT CACTCACCCC AAATAC
Taenio029    ---------- ---------- ------
Tarsiu030    TTTACGCCAT CACCCACCCC AAGTAC
Tinamu031    TTTATGCCAT CGGTCACCCC AAATAC
Trache032    ---------- ---------- ------
Xenopu033    TATATGCTAT TACACATCCC AAATAT
Bjar_O034    TTTATGCTAT TATTCACCCA AGTTAC
Cbic_O035    TTTATGCTAT TATTCACCCA AGTTAC
Cdur_O036    TTTATGCTAT TATTCACCCA AGTTAC
Dips_O037    TTTATGCTAT TATTCACCCA AGTTAC
Eceph_038    TTTATGCTAT TATTCACCCA AGTTAC
Eund_O039    TTTATGCTAT TATTCACCCA AGTTAC
Ery_OP040    TTTATGCTAT TATTCACCCA AGTTAC
Hel_OP041    TTTATGCTAT TATTCACCCA AGTTAC
Lmil_O042    TTTATGCTAT TATTCACCCA AGTTAC
Mcor_O043    TTTATGCTAT TATCCACCCA AGTTAC
Mlem_O044    TTTATGCTAT TATCCACCCA AGTTAC
Oguib_045    TTTATGCTAT TATTCACCCA AGTTAC
Arizon046    TTTATGCTAT TATTCACCCA AGTTAC
Cemoph047    TTTATGCTAT TATTCACCCA AGTTAC
Hypsig048    TTTATGCTAT TATTCACCCA AGTTAC
Lampro049    TTTATGCTAT TATTCACCCA AGTTAC
Pgutat050    TTTATGCTAT TATTCACCCA AGTTAC
Mastic051    TTTATGCTAT TATTCACCCA AGTTAC
Ppat_O052    TTTATGCTAT TATTCACCCA AGTTAC
Smik_O053    TTTATGCTAT TATTCACCCA AGTTAC
Snew_O054    TTTATGCTAT TATTCACCCA AGTTAC
Tae_OP055    TTTATGCTAT TATTCACCCA AGTTAC
Tham_O056    TTTATGCTAT TATTCACCCA AGTTAC
Tdor_O057    TTTATGCTAT TATTCACCCA AGTTAC
Anolis058    TCTATGCAAT CATTCACCCA AGATAC
Pogona059    TCTATGCAAT TATTCATCCA AGATAC
Protob060    TTTATGCTAT TATTCACCCA AGTTAC
Thamno061    TTTATGCTAT TATTCACCCA AGTTAC
Alliga062    TCTATGCAAT AATTCACCCA AAATAC
Athene063    TTTATGCCAT CACTCACCCC AAATAC
Callor064    TCTACGCCAT CACCCATCCT AAGTAT
Chelon065    TTTATGCCAT AACTCATCCC AAATAC
Chelon066    TCTATGCAAT AATTCACCCA AGATAC
Chryse067    TTTATGCCAT AACTCATCCC AAATAC
Danio_068    TCTACGCCAT TACGCATCCA AAGTAT
Danio_069    TCTACGCCAT CATACGCTCC AAATAC
Danio_070    TCTATGCCAT CACTCATCCC AAATAC
Danio_071    TATACGCCAT AATTCACAAC AAATAC
Egrett072    TTTATGCCAT CACTCACCCC AAATAC
Empido073    TTTATGCCAT CACTCACCCC AAATAC
Eptatr074    TGTACGCAAT CACTCACCCG AAATAC
Eptesi075    TTTACGCCAT CACCCACCCC AAGTAC
Erpeto076    TCTACGCAAT AACACATCCC AAATAC
Falco_077    TTTATGCCAT CACTCACCCC AAATAC
Gekko_078    TCTACGCAAT CATTCATCCA AGATAC
Hippog080    TCTACGCCAT TACACACCCC AAGTAC
Hippog081    TATACGCTAT TACACATCCA AAATAC
Hippog082    TCTACGCCAT CATCCACGCC AAATAC
Hippog083    TCTATGCAAT CATACATAAC AAATAC
Ictalu084    TTTATGCCAT CACGCATCCA AAATAC
Ictalu085    TCTATGCTAT TACACACCCT AAATAC
Larimi086    TCTACGCCAT CACACACCCG AAATAC
Lethen087    TGTACGCCAT CACACACCCC AAGTAC
Mastac088    TCTACGCCAT CACGCACCCA AAGTAC
Meleag089    TATATGCAAT AATTCACCCG AGATAC
Meleag090    TTTATGCCAT CACTCACCCT AAATAT
Notech091    TTTATGCTAT TATCCACCCA AGTTAC
```

```
Opisth092    TTTATGCCAT CACTCACCCC AAATAC
Pelodi093    TTTATGCCAT AACTCATCCC AAATAC
Phyllo094    TTTACGCCAT CACCCACCCT AAGTAC
Physet095    TTTATGCCAT CACCCACCCC AAGTAC
Pipra_096    TTTATGCCAT CACTCACCCC AAATAC
Podarc097    TCTATGCAAT CATTCACCCA AGATAC
Podarc098    TTTATGCCAT TGCCCACCCC AAATAC
Pseudo099    TTTATGCTAT TATCCACCCA AGTTAC
Python100    TTTACGCTAT CATTCACCCA AGTTAC
Salmo_101    TCTATGCCAT CACCCACCCA AAGTAC
Salmo_102    TTTATGCCAT TATACACACA AAGTAC
Salmo_103    TCTATGCCAT CATCCACTCT AAGTAC
Salmo_104    TCTATGCCAT CATCCACTCC AAATAC
Stegas105    TCTACGCCAT CACACACCCA AAGTAC
Terrap106    TTTATGCCAT AACTCATCCC AAATAC
Xenopu107    TCTATGCAAT TACACATCCC AAATAT
```
