# Supplementary material for: Characterization of the melanopsin gene (Opn4x) of diurnal and nocturnal snakes
Source: BMC Evol Biol. 2019 Aug 28;19:174. doi: 10.1186/s12862-019-1500-6 (PMC6714106; doi:10.1186/s12862-019-1500-6)
Supplement: Supplementary file 4 — Nucleotide alignment of melanopsin sequences of snakes used for random-site, branch-site and CmC models. (PDF 21 kb) [file 12862_2019_1500_MOESM4_ESM.pdf]

Hauzman et al. Additional File 4

```
          ....|....| ....|....| ....|....| ....|....| ....|....| ....|....| ....|....| ....|....| ....|....| ....|....| ....|....|
                 10         20         30         40         50         60         70         80         90        100        110
Pbivi     GGGAATCTCC TTGTTCTCTA TGCATTTTAC AGCAACAAGA GACTGAGGAC ACCTCCAAAT TATTTCATCA TGAATTTAGC CGTGAGTGAT TTTCTGATGT GTGCAACTCA
Pmucr     GGAAATCTCC TTGTCCTCTA TGCATTTTAC AGCAACAAGA GGCTGAGGAC ACCTGCAAAT TATTTCATTA TGAATTTAGC TGCAAGTGAC TTTCTGATGT CTGCAACTCA
Bjara     GgGaAtCtCc TtGtCcTcTa TgCaTtTtAc AgCaAcAaGa AgCtGaGaAc AcCtGcAaAt TaTtTcAtTa TgAaTtTaGc TgCaAgTgAc TtTcTgAtGt CtGcAaCtCa
Cduri     GGGAATCTCC TTGTCCTCTA TGCATTTTAC AGCAACAAGA GGCTGAGGAC ACCTGCAAAT TATTTCATTA TGAATTTAGC TGCAAGTGAC TTTCTGATGT CTGCAACTCA
Nscut     GGAAATCTCC TTGTCCTCTA TGCATTTTAC AGCAACAAGA GGCTGAGGAC ACCTGCAAAT TATTTCATCA TGAATTTAGC TGCAAGTGAC TTTCTGATGT CTGCAACTCA
Ptext     GGAAATCTCC TTGTCCTCTA TGCATTTTAC AGCAACAAGA GGCTGAGGAC ACCTGCAAAT TATTTCATCA TGAATTTAGC TGCAAGTGAC TTTCTGATGT CTGCAACTCA
Mcora     GGGAATCTCC TTGTCCTCTA TGCATTTTAC AGCAACAAGA AGCTGAGGAC ACCTGCAAAT TATTTCATCA TGAATTTAGC TGCAAGTGAC TTTCTGATGT CTGCAACTCA
Mlemn     GGGAATCTCC TTGTCCTCTA TGCATTTTAC AGCAACAAGA AGCTGAGGAC ACCTGCAAAT TATTTCATCA TGAATTTAGC TGCAAGTGAC TTTCTGATGT CTGCAACTCA
Tsirt     ---------- ---------- ---------- ---------- ---------- ---------- ---------A TGAATTTAGC TGCAAGTGAC TTTCTGATGT CTGCAACACA
Aeleg     ---------- ---------- ---------- ---AACAAGA GGCTGAGGAC ACCTGCAAAT TATTTCATCA TGAATTTAGC TGCAAGTGAC TTTCTGATGT CTGCAACTCA
Ccocc     ---------- ---------- ---------- ---------- ---------- ---------- ---------A TGAATTTAGC TGCAAGTGAC TTTCTGATGT CTGCTACTCA
Lgetu     ---------- ---------- ---------- ---------- ---------- ---------- ---------A TGAATTTAGC TGCAAGTGAC TTTCTGATGT CTGCAACTCA
Pguta     GGAAATCTCC TTGTACTCTA TGCATTTTAC AGCAACAAGA GGCTGAGGAC ACCTGCAAAT TATTTCATCA TGAATTTAGC TGCAAGTGAC TTTCTGATGT CTGCTACTCA
Mflag     ---------- ---------- ---------- ---------- ---------- ---------- ---------- ---------- ---------- ---------- ----------
Cbica     GGGAATCTCC TTGTCCTCTA TGCATTTTAC AGCAACAAGA GGCTGAGGAC ACCTGCAAAT TATTTCATCA TGAATTTAGC TGCAAGTGAC TTTCTGATGT CTGCAACTCA
Htorq     ---------- ---------- ---------- ---------- ---------- ---------- ---------A TGAATTTGGC TGCAAGTGAC TTAATGATGT CTGCAACTCA
Dindi     GGGAATCTCC TTGTCCTCTA TGCATTTTAC AGCAACAAGA GGCTGAGGAC ACCTGCAAAT TATTTCATCA TGAATTTGGC TGCAAGTGAC TTAATGATGT CTGCAACTCA
Smika     GGGAATCTCC TTGTCCTCTA TGCATTTTAC AGCAACAAGA GGCTGAGGAC ACCTGCAAAT TATTTCATCA TGAATTTGGC TGCAAGTGAC TTAATGATGT CTGCAACTCA
Sneuw     GGGAATCTCC TTGTCCTCTA TGCATTTTAC AGCAACAAGA GGCTGAGGAC ACCTGCAAAT TATTTCATCA TGAATTTGGC TGCAAGTGAC TTAATGATGT CTGCAACTCA
Tpers     GGGAATCTCA TTGTCCTCTA TGCATTTTAC AGCAACAAGA GGCTGAGGAC ACCTGCAAAT TATTTCATCA TGAATTTGGC TGCAAGTGAC TTTATGATGT CTGCAACTCA
Eceph     GGGAATCTCA TTGTCCTCTA TGCATTTTAC AGCAACAAGA GGCTGAGGAC ACCTGCAAAT TATTTCATCA TGAATTTRGC TGCAAGTGAC TTTATGATGT CTGCAACTCA
Eundu     GGGAATCTCA TTGTCCTCTA TGCATTTTAC AGCAACAAGA GGCTGAGGAC ACCTGCAAAT TATTTCATCA TGAATTTGGC TGCAAGTGAC TTTATGATGT CTGCAACTCA
Eaesc     GGGAATCTCC TTGTCCTCTA TGCATTTTAC AGCAACAAGA GGCTGAGGAC ACCTGCAAAT TATTTCATCA TGAATTTGGC TGCAAGTGAC CTTATGATGT CTGCAACTCA
Hmode     ---------- --------TA TGCATTTTAC AGCAACAAGA GGCTGAGGAC ACCTGCAAAT TATTTCATCA TGAATTTGGC TGCAAGTGAC TTTATGATGT CTGCAACTCA
Emili     GGGAATCTCC TTGTCCTCTA TGCATTTTAC AGCAACAAGA AGCTGAGGAC ACCTGCAAAT TATTTCATCA TGAATTTGGC TGCAAGTGAC TTTATGATGT CTGCAACTCA
Oguib     GGGAATCTCC TTGTCCTCTA TGCATTTTAT AGCAATAAGA AGTTGAGGAC ACCTGCAAAT TATTTCATCA TGAATTTGGC TGCAAGTGAC TTTATGATGT CTGCAACTCA
Ppata     GGGAATCTCC TTGTCCTCTA TGCATTTTAC AGCAACAAGA GGCTGAGGAC ACCTGCAAAT TATTTCATCA TGAATTTGGC TGCAAGTGAC TTTATGATGT CTGCAACTCA
Thypo     GGGAATCTCA TTGTCCTCTA TGCATTTTAC AGCAACAAGA GGCTGAGGAC ACCTGCAAAT TATTTCATCA TGAATTTGGC TGCAAGTGAC TTAATGATGT CTGCAACTCA
Tdors     ------CTCC TTGTCCTCTA TGCATTTTAC AGCAACAAGA GGCTGAGGAC ACCTGCAAAT TATTTCATCA TGAATTTGGC TGCAAGTGAC TTAATGATGT CTGCAACTCA


          ....|....| ....|....| ....|....| ....|....| ....|....| ....|....| ....|....| ....|....| ....|....| ....|....| ....|....|
                120        130        140        150        160        170        180        190        200        210        220
Pbivi     AGCTCCAGTC TGCTTTTTCA ACAGTATACA TAAGGAGTGG GTACTTGGAG ACACAGGTTG TAACTTCTAT GCTTTTTGTG GAGCACTCTT TGGAATAACC TCAATGATGA
Pmucr     AGCTCCAGTC TGCTTTTTCA ACAGTATGCA CAAGGAGTGG GTACTTGGAG ACACAGGTTG TAACTTTTAT GCTTTTTGTG GAGCACTGTT TGGAATAACC TCAATGATGA
Bjara     AgCtCcAgTc TgCtTtTtCa AcAgTaTgCa CaAgGaGtGg GtAcTtGgAg AcAcAgGtTg TaAcTtTtAt GcTtTtTgTg GaGcAcTkTt TgGaAtAaCc TcAaTgAtGa
Cduri     AGCTCCAGTC TGCTTTTTCA ACAGTATGCA CAAGGAGTGG GTACTTGGAG ACACAGGTTG TAACTTTTAT GCTTTTTGTG GAGCACTGTT TGGAATAACC TCAATGATGA
Nscut     AGCTCCAGTC TGCTTTTTCA ACAGTATGCA TAAGGAGTGG GTACTTGGAG ACACAGGTTG TAACTTCTAT GCTTTTTGTG GAGCTCTCTT CGGAATAACC TCAATGATGA
Ptext     AGCTCCAGTC TGCTTTTTCA ACAGTATGCA TAAGGAGTGG GTACTTGGAG ACGCAGGTTG TAACTTCTAT GCTTTTTGTG GTGCTCTCTT CGGAATAACC TCAATGATGA
Mcora     AGCTCCAGTC TGCTTTTTCA ACAGTATGCA TAAGGAGTGG GTACTTGGAG ACACAGGTTG TAGCTTCTAT GCTTTTTGTG GAGCTCTCTT CGGAATAACC TCAATGATGA
Mlemn     AGCTCCAGTC TGCTTTTTCA ACAGTATGCA TAAGGAGTGG GTACTTGGAG ACACAGGTTG TAGCTTCTAT GCTTTTTGTG GAGCTCTCTT CGGAATAACC TCAATGATGA
Tsirt     AGCTCCAGTC TGCTTTTTCA ACAGTCTGTA TACGGAGTGG GTTCTTGGAG ATGCAGGTTG TAACTTCTAT GCTTTTTGTG GAGCACTCTT TGGAATAACC TCAATGATGA
Aeleg     AGCTCCAGTC TGCTTTTTCA ACAGTATGCA TAAGGAGTGG GTACTTGGAG ACACAGGTTG TAACTTCTAT GCTTTTTGTG GAGCCCTCTT TGGAATAACC CCAATGATGA
Ccocc     AGCTCCAATC TGCTTTTTCA ACAGTATGCA TAAGGAGTGG GTACTTGGAG ACATAGGTTG TAACTTCTAT GCTTTTTGTG GAGCCCTCTT TGGAATAACT TCAATGATGA
Lgetu     AGCTCCAGTC TGCTTTTTCA ACAGTATGCA TAAGGAGTGG GTACTTGGAG ACATAGGTTG TAACTTCTAT GCTTTTTGTG GAGCACTCTT TGGAATAACC TCAATGATGA
Pguta     AGCTCCAGTC TGCTTTTTCA ACAGTATGCA TAAGGAGTGG GTACTTGGAG ACATAGGTTG TAACTTCTAT GCTTTTTGTG GAGCACTCTT TGGAATAACC TCAATGATGA
Mflag     ---------- ---------- ---------- ---------- ---------- ---------- ---------- ---------- ---------- ---------- ----------
Cbica     AGCTCCAGTC TGCTTTTTCA ACAGTATGCA TAAGGAGTGG GTACTTGGAG ACACAGGTTG TAACTTCTAT GCTTTTTGTG GAGCACTCTT TGGAATAACC TCAATGATGA
Htorq     AGCTCCAGTC TGCTTTTTCA ACAGTATGCA TAAGGAATGG GTACTTGGAG ACATAGGTTG TAACTTCTAT GCTTTTTGTG GAGCACTCTT TGGAATAACC TCAATGATGA
Dindi     AGCTCCAATC TGCTTTTTCA ACAGTATGCA TAAGGAGTGG GTTCTTGGAG ACATAGGTTG TAACTTCTAC GCTTTTTGTG GAGCACTCTT TGGAATAACC TCAATGATGA
Smika     AGCTCCAATC TGCTTTTTCA ACAGTATGCA TAAGGAGTGG ATTCTTGGAG ACATAGGTTG TAACTTCTAC GCTTTTTGTG GAGCACTCTT TGGAATAACC TCAATGATGA
Sneuw     AGCTCCAATC TGCTTTTTCA ACAGTATGCA TAAGGAGTGG GTTCTTGGAG ACATAGGTTG TAACTTCTAC GCTTTTTGTG GAGCACTCTT TGGAATAACC TCAATGATGA
Tpers     AGCTCCAGTC TGCTTTTTCA ACAGTATGAA AACAGAGTGG GTACTTGGAG ACATAGGTTG TAACTTCTAT GCTTTTTGTG GAGCACTCTT TGGAATAACC TCAATGATGA
Eceph     AGCTCCAGTC TGCTTTTTCA ACAGTATGAA AACAGAGTGG GTACTTGGAG ACATAGGTTG TAACTTCTAT GCTTTTTGTG GAGCACTCTT TGGAATAACC TCAATGATGA
Eundu     AGCTCCAGTC TGCTTTTTCA ACAGTATGAA AACAGAGTGG GTACTTGGAG ACATAGGTTG TAACTTCTAT GCTTTTTGTG GAGCACTCTT TGGAATAACC TCAATGATGA
Eaesc     AGCTCCAGTC TGCTTTTTCA ACAGTATGCA TACGGAGTGG GTACTTGGAG ACATAGGTTG TAACTTCTAT GCTTTTTGTG GAGCACTCTT TGGAATAACC TCAATGATGA
Hmode     AGCTCCAATC TGCTTTTTCA ACAGTATGCA AACAGAGTGG GTACTTGGAG ACATAGGTTG TAACTTCTAT GCTTTTTGTG GAGCACTCTT TGGAATAACC TCAATGATGA
Emili     AGCTCCAGTC TGCTTTTTCA ACAGTATGCA TACGGAGTGG GTACTTGGAG ACATAGGTTG TAACTTCTAT GCTTTTTGTG GAGCACTCTT TGGAATAACC TCAATGATGA
Oguib     AGCTCCAGTC TGCTTTTTCA ACAGTATGCG AACAGAGTGG GTACTTGGAG ACATAGGTTG TAACTTCTAT GCTTTTTGTG GAGCACTCTT TGGAATAACC TCAATGATGA
Ppata     AGCTCCAGTC TGCTTTTTCA ACAGTATGCA TACAGAGTGG GTACTTGGAG ACATAGGTTG TAACTTCTAT GCCTTTTGTG GAGCACTCTT TGGAATAACC TCAATGATGA
Thypo     AGCTCCAGTC TGCTTTTTCA ACAGCATGCA AACGGAGTGG GTACTTGGAG ACATAGGTTG TAACTTTTAT GCTTTTTGTG GAGCACTCTT TGGAATAACC TCAATGATGA
Tdors     AGCTCCAGTC TGCTTTTTCA ACAGTATGCA AACGGAGTGG GTACTTGGAG ACATAGGTTG TAACTTCTAT GCTTTTTGTG GAGCACTCTT TGGAATAACC TCAATGATGA


          ....|....| ....|....| ....|....| ....|....| ....|....| ....|....| ....|....| ....|....| ....|....| ....|....| ....|....|
                230        240        250        260        270        280        290        300        310        320        330
Pbivi     CCTTGTTAGC TATTTCAGTT GATCGCTACT GTGTGATTAC TAAGCCTCTG CAGTCCATAA AAAGGAGTTC AAAGAAACGC TCATGTCTAA TCATTATGTT TGTCTGGCTT
Pmucr     CCTTGTTGGC TATTTCGGTG GATCGCTACT GTGTGATTAC AAAGCCTCTC CAGTCATTAA AAAGAAGTTC AAAGAAACGT TCATGTATAA TCATTATGTT TGTCTGGCTC
Bjara     CcTtGtTgGc TaTtTcGgTg GaTcGcTaCt GtGtGaTtAc CaAgCcTcTc CaGtCaTtAa AaAgAaGtTc AaAgAaAcGt TcGtGtAtAa TcAtTaTgTt TgTcTgGcTc
Cduri     CCTTGTTGGC TATTTCGGTG GATCGCTACT GTGTGATTAC CAAGCCTCTC CAGTCATTAA AAAGAAGTTC AAAAAAACGT TCATGTATAA TCATTATGTT TGTCTGGCTC
Nscut     CCTTGCTAGC TATTTCGGTG GATCGCTACT GTGTGATCAC AAAACCTCTC CAGTCCTTAA AAAGAAGTAC AAAGAAACGC TCTTATATAA TCATCATGTT TGTCTGGCTG
Ptext     CCTTGTTAGC TATTTCGGTG GATCGCTACT GTGTGATCAC AAAACCTCTC CAGTCCTTAA AAAGAAGTAC AAAGAAACGC TCTTATATAA TCATCATGTT TGTCTGGCTG
Mcora     CCTTGTTAGC TATTTCGGTG GATCGCTACT GTGTGATCAC AAAACCTCTC CAGTCCTTAA AAAGAAGTAC AAAGAAACGC TCTTGTATAA TCATCATGTT TGTCTGGCTC
Mlemn     CCTTGTTAGC TATTTCGGTG GATCGCTACT GTGTGATCAC AAAACCTCTC CAGTCCTTAA AAAGAAGTAC AAAGAAACGC TCTTGTATAA TCATCATGTT TGTCTGGCTC
Tsirt     CCTTGTTAGC TATTTCGGTG GATCGCTACT GTGTGATTAC AAAGCCTCTC CAGTCCTTAA AAAGAAGTAC AAAGAAACGT TCTTGTATAA TCATTATATT TGTCTGGCTC
Aeleg     CCTTGTTAGC TATTTCGGTG GATCGCTACT GTGTGATTAC GAAGCCTCTC CAGTCCTTAA AGAGAAGTTC AAAGAAACGT TCTTGTATAA TCATTATATT TGTCTGGCTC
Ccocc     CCTTGTTAGC TATTTCGGTG GATCGCTACT GTGTGATTAC GAAGCCTCTC CAGTCCTTAA AAAGAAGTTC AAAGAAACGT TCTTGCATAA TCATTATATT TGTCTGGCTC
Lgetu     CCTTGTTAGC TATTTCGGTG GATCGCTACT GTGTGATTAC GAAGCCTCTC CAGTCCTTAA AAAGAAGTTC AAAGAAACGT TCTTGTATAA TCATTATATT TGTCTGGCTC
Pguta     CCTTGTTAGC TATTTCGGTG GATCGCTACT GTGTGATTAC GAAGCCTCTC CAGTCCTTAA AAAGAAGTTC AAAGAAACGT TCTTGTATAA TCATTATATT TGTCTGGCTC
Mflag     ---------- ---------- ---------- ---------- ---------- ---------- ---------- ---------- ---------- ---------- ----------
Cbica     CCTTGTTAGC TATTTCGGTC GATCGCTACT GTGTGATTAC GAAGCCTCTC CAGTCCTTAA AAAGAAGTTC AAAGAAACGT TCTTGTATAA TCATTATATT TGTCTGGCTC
Htorq     CCTTGTTAGC TATTTCGGTG GATCGCTACT GTGTAATTAC AAAGCCTCTC CAGTCCTTAA AAAGAAGTTC AAAGAAACGT TCTTGTATAA TCATTATATT TGTCTGGCTC
Dindi     CCTTGTTAGC TATTTCAGTG GATCGCTACT GTGTGATTAC AAAGCCTCTC CAGTCCTTAA AAAGAAGTTC AAAGAAACGT TCTTGTATAA TCATTATATT TGTCTGGCTC
Smika     CCTTGTTAGC TATTTCGGTG GATCGCTACT GTGTGATTAC AAAGCCTCTC CAGTCCTTAA AAAGAAATTC AAAGAAACGT TCTTGTATAA TCATTATATT TGTCTGGCTC
Sneuw     CCTTGTTAGC TATTTCGGTG GATCGCTACT GTGTGATTAC AAAGCCTCTC CAGTCCTTAA AAAGAAGTTC AAAGAAACGT TCTTGTATAA TCATTATATT TGTCTGGCTC
Tpers     CCTTGTTAGC TATTTCGGTG GATCGCTACT GTGTGATTAC AAAGCCTCTC CAGTCYTTAA AAAGAAGTTC AAAGAAACGT TCTTGTATAA TCATTATATT TGTCTGGCTC
Eceph     CCTTGTTAGC TATTTCGGTG GATCGCTACT GTGTGATTAC AAAGCCTCTC CAGTCCTTAA AAAGAAGTTC AAAGAAACGT TCTTGTATAA TMATTATATT TGTCTGGCTC
Eundu     CCTTGTTAGC TATTTCGGTG GATCGCTACT GTGTGATTAC AAAGCCTCTC CAGTCCTTAA AAAGAAGTTC AAAGAAACGT TCTTGTATAA TCATTATATT TGTCTGGCTC
Eaesc     CCTTGTTAGC TATTTCGGTG GATCGCTACT GTGTGATTAC AAAGCCTCTC CAGTCCTTAA AAAGAAGTTC AAAGAAACGT TCTTGTATAA TCATTATACT TGTCTGGCTC
Hmode     CCTTGTTAGC TATTTCAGTG GATCGCTACT GTGTGATTAC AAAGCCTCTC CAGTCCTTAA AAAGAAGTTC AAAGAAACGT TCTTGCATAA TCATTATATT TGTCTGGCTC
Emili     CCTTGTTAGC AATTTCGGTG GATCGCTACT GTGTGATTAC AAAACCTCTC CAGTCCTTAA AAAGAAGTTC AAAGAAACGT TCTTGTATAA TCATTATACT TGTCTGGCTC
Oguib     CCTTGTTAGC TATTTCGGTG GATCGCTACT GTGTGATTAC AAAGCCTCTC CAGTCCTTAA AAAGAAGTTC AAAGAAACGT TCTTGTATAA TCATTATATT TGTCTGGCTC
Ppata     CCTTGTTAGC TATTTCGGTG GATCGCTACT GTGTGATTAC AAAGCCTCTC CAGTCCTTAA AAAGAAGTTC AAAGAAACGT TCTTGTATAA TCATTATATT TGTCTGGCTC
Thypo     CCTTGTTAGC TATTTCCGTG GATCGCTACT GTGTGATTAC AAAGCCTCTC CAGTCCTTAA AAAGAAGTTC AAAGAAACGT TCTTGTATAA TCATTATATT TGTCTGGCTC
Tdors     CCTTGTTAGC TATTTCCGTG GATCGCTACT GTGTGATTAC AAAGCCTCTC CAGTCCTTAA AAAGAAGTTC AAAGAAACGT TCTTGTATAA TCATTATATT TGTCTGGCTC


          ....|....| ....|....| ....|....| ....|....| ....|....| ....|....| ....|....| ....|....| ....|....| ....|....| ....|....|
                340        350        360        370        380        390        400        410        420        430        440
Pbivi     TACTCACTGG GTTGGAGTGT GTGTCCATTA TTTGGGTGGA GTTCCTATAT CCCAGAAGGT CTGATGATAT CTTGTACTTG GGACTATGTA ACCTATTCTC CAGCAAACAG
Pmucr     TACTCACTGG CTTGGAGTGT TTGTCCATTA TTCGGGTGGA GTTCCTATAT CCCAGAAGGT TTGATGATAT CCTGTACATG GGACTATGTA AGCTATTCTC CAGCAAACAG
Bjara     TaCtCaCtGg CtTgGaGtGt TtGtCcAaTa TtCgGgTgGa GtTcCtAtAt CcCaGaAgGt TtGaTgAtAt CcTgTaCaTg GgAcTaTaTa AgCtAtTcTc CaGcAaAcAg
```

```
Cduri   TACTCACTGG CTTGGAGTGT TTGTCCATTA TTTGGGTGGA GTTCCTATAT CCCAGAAGGT TTGATGATAT CCTGTACATG GGACTATGTA AGCTATTCTC CAGCAAACAG
Nscut   TACTCACTGG GTTGGAGCGT ATGTCCATTA TTCGGGTGGA GTTCCTATAT CCCAGAAGGT TTGATGATAT CCTGTACATG GGACTATGTA AGCTATTCTC CATCAAACAG
Ptext   TACTCACTGG GTTGGAGCGT ATGTCCATTA TTTGGGTGGA GTTCCTATAT CCCAGAAGGT TTGATGATAT CCTGTACATG GGACTATGTA AGCTATTCTC CATCAAACAG
Mcora   TACTCACTGG GTTGGAGCGT ATGTCCATTA TTCGGGTGGA GTTCCTATAT CCCCGAAGGT TTGATGATAT CCTGTACATG GGACTATGTA AGCTATTCTC CAGCAAACAG
Mlemn   TACTCACTGG GTTGGAGCGT ATGTCCATTA TTCGGGTGGA GTTCCTATAT CCCAGAAGGT TTGATGATAT CCTGTACATG GGACTATGTA AGCTATTCTC CAGCAAACAG
Tsirt   TACTCACTGG GTTGGAGTGT ATGTCCATTA TTCGGATGGA GTTCCTATAT CCCAGAAGGT TTGATGATAT CCTGTACATG GGACTATGTA AGCTATTCTC CCGGAAACAG
Aeleg   TACTCACTGG GTTGGAGTGT GTGTCCATTA TTCGGGTGGA GTTCCTATAT CCCAGAAGGT TTGATGATAT CCTGTACATG GGACTATGTA AGCTATTCTC CAGGAAACAG
Ccocc   TACTCACTGG GTTGGAGTGT GTGTCCATTA TTCGGGTGGA GTTCCTATAT CCCAGAAGCT TTGATGATAT CCTGTACATG GGACTATGTA AGCTATTCTC CAGGAAACAG
Lgetu   TACTCACTGG GTTGGAGTGT GTGTCCATTA TTCGGGTGGA GTTCCTATAT CCCAGAAGGT TTGATGATAT CCTGTACATG GGACTATGTA AGCTATTCTC CAGGAAACAG
Pguta   TACTCACTGG GTTGGAGTGT GTGTCCATTA TTCGGGTGGA GTTCCTATAT CCCAGAAGGT TTGATGATAT CCTGTACATG GGACTATGTA AGCTATTCTC CAGGAAACAG
Mflag   ---------- ---------- ---------- ---------- --TCCTATAT CCCAGAAGGT TTGATGATAT CCTGTACATG GGACTATGTA AGCTATTCTC CAGGAAACAG
Cbica   TACTCACTGG GTTGGAGTGT ATGTCCATTA TTCGGGTGGA GTTCCTATAT CCCAGAAGGT TTGATGATAT CCTGTACATG GGACTATGTA AGCTATTCTC CAGGAAACAG
Htorq   TACTCACTGG CTTGGAGTGT ATGTCCATTA TTCGGGTGGA GTTCCTATGT CCCAGAAGGT TTGATGATAT CCTGTACATG GGACTATGTA AGCTATTCCC CAGCAAACAG
Dindi   TACTCACTGG CTTGGAGTGT ATGTCCATTA TTCGGGTGGA GTTCCTATAT CCCAGAAGGT TTGATGATAT CCTGTACATG GGACTATGTA AGCTATTCTC CAGCAAACAG
Smika   TACTCACTGG CTTGGAGTRT ATGTCCATTA TTCGGGTGGA GTTCCTATAT CCCAGAAGGT TTGATGATAT CCTGTACATG GGACTATGTA AGCTATTCTC CAGCAAACAG
Sneuw   TACTCACTGG CTTGGAGTGT ATGTCCATTA TTCGGGTGGA GTTCCTATAT CCCAGAAGGT TTGATGATAT CCTGTACATG GGACTATGTA AGCTATTCTC CAGCAAACAG
Tpers   TACTCACTGG GTTGGAGTGT ATGTCCATTA TTCGGGTGGA GTTCCTATAT CCCAGAAGGT TTGATGATAT CCTGTACATG GGACTATGTA AGCTATTCTC CAGGAAACAG
Eceph   TACTCACTGG GTTGGAGTGT ATGTCCATTA TTYGGGTGGA GTTCCTATAT CCCAGAAGGT TTGATGATAT CCTGTACATG GGACTATGTA AGCTATTCTC CAGGAAACAG
Eundu   TACTCACTGG GTTGGAGTGT ATGTCCATTA TTCGGGTGGA GTTCCTATAT CCCAGAAGGT TTGATGATAT CCTGTACATG GGACTATGTA AGCTATTCCC CAGGAAACAG
Eaesc   TACTCGCTGG GTTGGAGCGT GTGTCCATTA TTTGGGTGGA GTTCCTATAT CCCAGAAGGT TTAATGATAT CCTGTACATG GGACTATGTA AGCTATTCTC CAGGAAACAG
Hmode   TACTCACTGG GTTGGAGTGT ATGTCCATTA TTCGGGTGGA GTTCCTATAT CCCAGAAGGT TTGATGATAT CCTGTACATG GGACTATGTA AGCTATTCTC CAGCAAACAG
Emili   TACTCGCTGG GTTGGAGCGT ATGTCCATTA TTTGGGTGGA GTTCCTATAT CCCAGAAGGT TTGATGATAT CCTGTACGTG GGACTATGTA AGCTATTCTC CAGGAAACAG
Oguib   TACTCACTGG GTTGGAGTGT ATGTCCATTA TTCGGGTGGA GTTCCTATAT CCCAGAAGGT TTGATGATAT CCTGTACATG GGACTATGTA AGCTATTCTC CAGCAAACAG
Ppata   TACTCACTGG GTTGGAGTGT ATGTCCATTA TTCGGGTGGA GTTCCTATAT CCCAGAAGGT TTGATGATAT CCTGTACATG GGACTATGTA AGCTATTCTC CAGGAAACAG
Thypo   TACTCACTGG GTTGGAGTGT ATGTCCATTA TTCGGGTGGA GTTCCTATAT CCCAGAAGGT TTGATGATAT CCTGTACATG GGACTATGTA AGCTATTCTC CAGCAAACAG
Tdors   TACTCACTGG GTTGGAGTGT ATGTCCATTA TTCGGGTGGA GTTCCTATAT CCCAGAAGGT TTGATGATAT CCTGTACATG GGACTATGTA AGCTATTCTC CAGCAAACAG

        ....|....| ....|....| ....|....| ....|....| ....|....| ....|....| ....|....| ....|....| ....|....| ....|....| ....|....|
                450        460        470        480        490        500        510        520        530        540        550
Pbivi   AAGTTACACC ATGTTGCTAT GTTGCTGTGT GTTCTTCATT CCACTGATAA TAATCTTCCA CTGCTATTTA TGCATGTTTC TAGCCATAAG AAATACTGGC AGGAATGTTC
Pmucr   AAGTTACACC ATGTTGCTAT GTTGGTGTGT GTTCTTCATT CCTCTTATAA TAATCTTCCA TTGCTATTTA TTCATGTTCC TCACCATAAG AAGTACAGGC AGGAATGTTC
Bjara   AaGtTaCaCc AtGtTgCtAt GtTgGtGtGt GtTcTtCaTt CcTcTcAtAa TaAtCtTcCa TtGcTaTtTa TtCaTgTtCc TcAcCaTaAg AaGgAcAgGc AgGaAtGtTc
Cduri   AAGTTACACC ATGTTGCTAT GTTGGTGTGT GTTCTTCATT CCTCTCATAA TAATCTTCCA TTGCTATTTA TTCATGTTCC TCACCATAAG AAGTACAGGC AGGAATGTTC
Nscut   AAGTTACACC ATGTTGCTAT GTTGGTGTGT GTTCTTCATT CCTCTTATGA TAATCTTCCA TTGCTATTTA TTCATGTTCC TCACCATAAG AAGTACAGGC AGGAATGTTC
Ptext   AAGTTACACC ATGTTGCTAT GTTGGTGTGT GTTCTTCATT CCTCTTATGA TAATCTTCCA TTGCTATTTA TTCATGTTCC TCACCATAAG AAGTACAGGC AGGAATGTTC
Mcora   AAGTTACACC ATGTTGCTAT GTTGGTGTGT GTTCTTCATT CCTCTTATGA TAATCTTCCA TTGCTATTTA TACATGTTCC TCACCGTAAG AAGTACAGGC AGGAACGTTC
Mlemn   AAGTTACACC ATGTTGCTAT GTTGGTGTGT GTTCTTCATT CCTCTTATGA TAATCTTTCA TTGCTATTTA TTCATGTTCC TCACCATAAG AAGTACAGGC AGGAATGTTC
Tsirt   AAGTTACACC ATGTTGCTAT GTTGGTGTGT GTTCTTCATT CCTCTTATGA TAATCTTCCA TTGCTATTTA TTCATGTTCC TCACCATCAG AAGTACAGGC AGGAATGTTC
Aeleg   AAGTTACACC ATGTTGCTAT GTTGGTGTGT GTTCTTCATT CCTCTTATGA TAATCTTCCA TTGCTATTTA TTCATGTTCC TCACCATCAG AAGTACAGGC AGGAATGTTC
Ccocc   AAGTTACACC ATGTTGCTAT GTTGGTGTGT GTTCTTCATT CCTCTTATGA TAATCTTCCA TTGCTATTTA TTCATGTTCC TCACCATCAG AAGTACAGGC AGGAATGTTC
Lgetu   AAGTTACACC ATGTTGCTAT GTTGGTGTGT GTTCTTCATT CCTCTTATGA TAATCTTCCA TTGCTATTTA TTCATGTTCC TCACCATCAG AAGTACAGGC AGGAATGTTC
Pguta   AAGTTACACC ATGTTGCTAT GTTGGTGTGT GTTCTTCATT CCTCTTATGA TAATCTTCCA TTGCTATTTA TTCATGTTCC TCACCATCAG AAGTACAGGC AGGAATGTTC
Mflag   AAGTTACACC ATGTTGCTAT GTTGGTGTGT GTTCTTCATT CCTCTTATGA TAATCTTCCA TTGCTATTTA TTCATGTTCC TCACCATCAG AAGTACAGGC AGGAATGTTC
Cbica   AAGTTACACC ATGTTGCTAT GTTGGTGTGT GTTCTTTATT CCTCTTATGA TAATCTTCCA TTGCTATTTA TTCATGTTCC TCACCATCAG AAGTACAGGC AGGAATGTTC
Htorq   AAGTTATACC ATGTTGCTAT GTTGGTGTGT GTTCTTCATT CCTCTTATGA TAATCTTCCA TTGCTATTTA TTCATGTTCC TCACCATCAG AAGTACAGGC AGGAATGTTC
Dindi   AAGTTATACC ATGTTGCTAT GTTGGTGTGT GTTCTTCATT CCACTTATGA TAATCTTCCA TTGCTATTTA TTCATGTTCC TCACCATCAG AAGTACAGGC AGGAATGTTC
Smika   AAGTTATACC ATGTTGCTAT GTTGGTGTGT GTTCTTCATT CCACTTATGA TAATCTTCCA TTGCTATTTA TTCATGTTCC TCACCATCAG AAGTACAGGC AGGAATGTTC
Sneuw   AAGTTATACC ATGCTGCTAT GTTGGTGTGT GTTCTTCATT CCACTTATGA TAATCTTCCA TTGCTATTTA TTCATGTTCC TCACCATCAG AAGTACAGGC AGGAATGTTC
Tpers   AAGCTACACT ATGTTGCTAT GTTGGTGTGT STTCTTCATT CCTCTTATGA TAATCTTCCA TTGCTATTTA TTCATGTTCC TCACCATCAG AAGTACAGGC AGGAATGTTC
Eceph   AAGTTACACG ATGTTGCTAT GTTGGTGTGT GTTCTTCATT CCTCTTATGA TAATCTTCCA TTGCTATTTA TTCATGTTCC TCACCATCAG AAGTACAGGC AGGAATGTTC
Eundu   AAGTTACACG ATGTTGCTAT GTTGGTGTGT GTTCTTCATT CCTCTTATGA TAATCTTCCA TTGCTATTTA TTCATGTTCC TCACCATCAG AAGTACAGGC AGGAATGTTC
Eaesc   AAGTTACACT ATGTTGCTAT GTTGGTGTGT GTTCTTCATT CCTCTTATGA TCATCTTCCA TTGCTATTTA TTCATGTTCC TCACCATCAG AAGTACAGGC AGGAATGTTC
Hmode   AAGTTACACT ATGTTGCTAT GTTGGTGTGT GTTCTTCATT CCTCTTATGA TAATCTTCCA TTGCTATTTA TTCATGTTCC TCACCATCAG AAGTACAGGC AGGAATGTTC
Emili   AAGTTACACT ATGTTGCTAT GTTGGTGTGT GTTCTTCATT CCTCTTATGA TCATCTTCCA TTGCTATTTA TTCATGTTCC TCACCATCAG AAGTACAGGC AGGAATGTTC
Oguib   AAGTTACACT ATGTTGCTAT GCTGGTGTGT GTTCTTCATT CCTCTTATGA TAATCTTCCA TTGCTATTTA TTCATGTTTC TCACCATCAG AAGTACAGGC AGGAATGTTC
Ppata   AAGTTACACT ATGTTGCTAT GTTGGTGTGT GTTCTTCATT CCTCTTATGA TAATCTTCCA TTGCTATTTA TTCATGTTCC TCACCATCAG AAGTACAGGC AGGAATGTTC
Thypo   AAGTTACACT ATGTTGCTAT GTTGGTGTGT GTTCTTCATT CCTCTTATGA TAATCTTCCA TTGCTATTTA TTCATGTTCC TCACCATCAG AAGTACAGGC AGGAATGTTC
Tdors   AAGTTACACT ATGTTGCTAT GTTGGTGTGT GTTCTTCATT CCTCTTATGA TAATCTTCCA TTGCTATTTA TTCATGTTCC TCACCATCAG AAGTACAGGC AGGAATGTTC

        ....|....| ....|....| ....|....| ....|....| ....|....| ....|....| ....|....| ....|....| ....|....| ....|....| ....|....|
                560        570        580        590        600        610        620        630        640        650        660
Pbivi   AGAAATTAGG ATCCAGCTAT AAACGGAAAC ATTCAGCCTC ACAGAACATA AAAAGTGAAT GGAAACTGGC AAAAATTGCC TTTGTAGCCA TTGTGGTTTA TGTTGTATCC
Pmucr   AGAAATTAGG ATCCAGTTCT AAACGAAAAC AGTCAAGCTC ACAGAGCATA AAGAGTGAAT GGAAACTGGC AAAAGTTGCC TTCGTAGCCA TTGTGGTTTA TGTTGTATCC
Bjara   AgAaAtTaGg AtCcAgTtCt AaAcGaAaAc AgTcAaGcTc AcAgAgCaTa AaGaGtGaAt GgAaAcTgGc AaAaGtTgCc TtCgTaGcCa TtGtGgTtTa TgTtGtAtCc
Cduri   AGAAATTAGG ATCCAGTTCT AAACGAAAAC AGTCAAGCTC ACAGAGCATA AAGAGTGAAT GGAAACTGGC AAAAGTTGCC TTCGTAGCCA TTGTGGTTTA TGTTATATCC
Nscut   AGAAATTAGG ATCCACCTCT AAACGGAAAA AGTCAAGCTC ACAGAGCATA AAGAATGAAT GGAAACTAGC AAAAGTTGCC TTCGTAGCCA TTGTGGTTTA TGTTGTATCC
Ptext   AGAAATTAGG ATCCACCTCT AAACGGAAAA AGTCAAGCTC GCAGAGCATA AAGAATGAAT GGAAACTAGC AAAAGTTGCC TTCGTAGCCA TTGTGGTTTA TGTTGTATCC
Mcora   AGAAATTAGG ATCTAGCTCT AAACGGAAAA AATCAAGCTC ACAGAGCATA AAGAATGAAT GGAAACTAGC AAAAGTTGCC TTCGTAGCCA TTGTGGTTTA TGTTGTATCC
Mlemn   AGAAATTAGG ATCTAGCTCT AAACGGAAAA AGTCAAGCTC ACAGAGCCTA AAGAATGAAT GGAAACTAGC AAAAGTTGCC TTCGTAGCCA TTGTGGTTTA TGTTGTATCC
Tsirt   AGAAATTGGG ATCCAGCACT AAACGGAAAA AGTCAAGCTC ACACAGCATA AAGAATGAAT GGAAGCTGGC CAAGGTTGCC TTCGTAGCCA TTGTGGTTTA TGTTGTATCT
Aeleg   AGAAATTGGG ATCTAGCACT AAACGGAAAA AATCAAGCTC ACATAACATA AAGAATGAGT GGAAACTGGC AAAGGTTGCC TTTGTAGCTA TTGTGGTTTA CGTTGTATCC
Ccocc   AGAAATTGGG ATCTAGCACT AAACGGAAAA AATCAAGCTC ACAGAACATA AAGAATGAAT GGAAACTGGC AAAGGTTGCC TTCGTAGCTA TTGTGGTTTA CGTTGTATCC
Lgetu   AGAAATTGGG ATCTAGCACT AAACGGAAAA AATCAAGCTC ACAGAACATA AAGAATGAAT GGAAACTGGC AAAGGTTGCC TTCGTAGCTA TTGTGGTTTA CGTTGTATCC
Pguta   AGAAATTGGG ATCTAGCACT AAACGGAAAA AATCAAGCTC ACAGAACATA AAGAATGAAT GGAAACTGGC AAAGGTTGCC TTCGTAGCTA TTGTGGTTTA TGTTGTATCC
Mflag   AGAAATTGGG ATCTAGCACT AAACGGAAAA AATCAAGCTC ACAGAACATA AAGAATGAAT GGAAACTGGC AAAGGTTGCC TTCGTAGCTA TTGTGGTTTA CGTTGTATCC
Cbica   AGAAATTGGG ATCTAGCACT AAACGGAAAA AGTCAAGCTC ACAGAGCATA AAGAATGAAT GGAAACTGGC AAAGGTTGCC TTCGTAGCTA TTGTGGTTTA CGTTGTATCC
Htorq   AGAAATTGGG ATCCAGCTCT AAACGGAAAA AGTCAAGCTC ACAGAGTATA AAGAGTGAAT GGAAACTGGC AAAAGTTGCC TTCGTAGCCA TTGTGGTTTA CGTTGTATCC
Dindi   AGAAATTGGG ATCCAGCTCT AAACGGAAAA AGTCAAGCTC ACAGAGCATA AAGAGTGAAT GGAAACTAGC AAAAGTTGCC TTTGTAGCCA TTGTGGTTTA CGTTGTATCC
Smika   AGAAATTGGG ATCCAGCTCT AAGCGGAAAA AGTCAAGCTC ACAGAGCATA AAGAGTGAAT GGAAACTAGC AAAAGTTGCC TTAGTAGCCA TTGTGGTTTA CGTTGTATCC
Sneuw   AGAAATTGGG ATCCAGCTCT AAACGGAAAA AGTCAAGCTC ACAGAGCATA AAGAGTGAAT GGAAACTAGC AAAAGTTGCC TTAGTAGCCA TTGTGGTTTA CGTTGTATCC
Tpers   AGAAATTGGG ATCCAGCTCT AAAYGGAAAA AGTCAAGCTC ACAGAGCATA AAGAATGAAT GGAAACTGGC AAAAGTTGCC TTCGTAGCCA TTGTGGTTTA CGTTGTATCC
Eceph   AGAAATTGGG ATCCAGCTCT AAACGGAAAA AGTCAAGCTC ACAGAGCATA AAGAATGAAT GGAAACTGGC AAAAGTTGCY TTCGTAGCCA TTGTGGTTTA CGTTGTATCC
Eundu   AGAAATTGGG ATCCAGCTCT AAACGGAAAA AGTCAAGCTC ACAGAGCATA AAGAATGAAT GGAAACTGGC AAAAGTTGCC TTCGTAGCCA TTGTGGTTTA CGTTGTATCC
Eaesc   AGAAATTGGG ATCCAGCTCT AAACGGAAAA AGTCAAGCTC ACAAAGCATA AAGAATGAAT GGAAACTGGC CAAAGTTGCC TTCGTAGCCA TTGTGGTTTA CGTTGTATCC
Hmode   AGAAATTGGG ATCCAGCTCT AAACGGAAAA AGTCAAGCTC ACACAGCATA AAGAATGAAT GGAAACTGGC AAAAGTTGCC TTCGTAGCCA TTGTGGTTTT CGTTGTATCC
Emili   AGAAATTGGG ATCCAGCTCT AAACGGAAAA AGTCAAGCTC ACACAGCATA AAGAATGAAT GGAAACTGGC CAAAGTTGCC TTCGTAGCCA TTGTGGTTTA CGTTGTATCC
Oguib   AGAAATTGGG ATCCAGCTCT AAACGGAAAA AGTCAAGCTC ACAGAGCATA AAGAATGAAT GGAAACTGGC AAAAGTTGCC TTTGTAGCCA TTGTGGTTTA CGTTGTATCC
Ppata   AGAAATTGGG ATCCAGCTCT AAACGGAAAA AGTCAAGCTC ACAGAGCATA AAGAATGAAT GGAAACTGGC AAAAGTTGCC TTCGTAGCCA TTGTGGTTTA CGTTGTATCC
Thypo   AGAAATTGGG ATCCAGCTCT AAACGGAAAA AGTCAAGCTC ACAGAGCATA AAGAATGAAT GGAAACTGGC AAAAGTTGCC TTCGTAGCCA TTGTGGTTTA CGTTGTATCC
Tdors   AGAAATTGGG ATCCAGCTCT AAACGGAAAA AGTCAAGCTC ACAGAGCATA AAGAATGAAT GGAAACTGGC AAAAGTTGCC TTCGTGGCCA TTGTGGTTTA CGTTGTATCC

        ....|....| ....|....| ....|....| ....|....| ....|....| ....|....| ....|....| ....|....| ....|....| ....|....| ....|....|
                670        680        690        700        710        720        730        740        750        760        770
Pbivi   TGGTCTCCAT ATGCTTGTGT TACCTTGATT GCCTGGGCTG GCTATGCCAG GATCTTAACT CCATATTCTA AATCTGTGCC TGCTATTATT GCCAAAGCTT CTGCAATTTA
Pmucr   TGGTCTCCGT ACGCTTGTGT CACCCTGATT GCATGGGCTG GTTATGCCAG GGTCTTAACT CCGTATTCTA AATCTGTGCC TGCTGTTATT GCCAAAGCTT CAGCAATTCA
Bjara   TgGtCtCcAt AtGcTtGtGt CaCcCtGaTt GcAtGgGcTg GtTaTgCcAg AaTcTtAaCt CcGtAtTcTa AaTcTgTgCc TgCtAtTaTt GcTaAaGcTt CaGcAaTtCa
Cduri   TGGTCTCCGT ATGCTTGTGT CACCCTGATT GCATGGGCTG GTTATGCCAG GATCTTAACT CCATATTCTA AATCTGTGCC TGCTGTTATT GCTAAAGCTT CAGCAATTCA
Nscut   TGGTCTCCGT ATGCTTGTGT CACCCTGATT GCATGGGCTG GTTATGCCAG GGTCTTAACT CCATATTCTA AGTCTGTGCC TGCTGTTATT GCCAAAGCTT CAGCAATTCA
Ptext   TGGTCTCCGT ATGCTTGTGT CACCCTGATT GCATGGGCTG GTTATGCCAG GGTCTTAACT CCATATTCTA AGTCTGTGCC TGCTGTTATT GCCAAAGCTT CAGCAATTCA
Mcora   TGGTCTCCAT ATGCTTGTGT CACCCTGATT GCATGGGCTG GTTATGCCAG GGTCTTAACT CCATATTCTA AGTCTGTGCC TGCTGTTATT GCCAAAGCTT CAGCAATTCA
Mlemn   TGGTCTCCAT ATGCTTGTGT CACCCTGATT GCATGGGCTG GTTATGCCAG GGTCTTAACT CCATATTCTA AGTCTGTGCC TGCTGTTATT GCCAAAGCTT CAGCAATTCA
Tsirt   TGGTCTCCGT ATGCTTGTGT CACTCTGATT GCATGGGCCG GTTATGCCAG AATCTTAACT CCATATTCTA AGTCTGTGCC TGCTGTTATT GCCAAAGCTT CAGCAATTCA
Aeleg   TGGTCTCCGT ATGCTTGTGT CACTCTAATT GCATGGGCTG GTTATGCCAA GATCTTAACT CCATATTCTA AATCTGTGCC TGCTGTTATT GCCAAAGCTT CAGCAATTCA
Ccocc   TGGTCTCCGT ATGCTTGTGT CACTCTGATT GCATGGGCTG GTTATGCCAA GATCTTAACT CCATATTCTA AATCTGTGCC TGCTGTTATT GCCAAAGCTT CAGCAATTCA
```

```
Lgetu    TGGTCTCCGT ATGCTTGTGT CACTCTGATT GCATGGGCTG GTTATGCCAA GATCTTAACT CCATATTCTA AATCTGTGCC TGCTGTTATT GCCAAAGCTT CAGCAATTCA
Pguta    TGGTCTCCGT ATGCTTGTGT CACTCTGATT GCATGGGCTG GTTATGCCAA GATCTTAACT CCATATTCTA AATCTGTGCC TGCTGTTATT GCCAAAGCTT CAGCAATTCA
Mflag    TGGTCTCCGT ATGCTTGTGT CACTCTGATT GCATGGGCTG GTTATGCCAA GATCTTAACT CCATATTCTA AATCTGTGCC TGCTGTTATT GCCAAAGCTT CAGCAATTCA
Cbica    TGGTCTCCAT ATGCTTGTGT CACTCTGATT GCATGGGCTG GTTATGCCAA RATCTTAACT CCATATTCTA AATCTGTGCC TGCTGTTATT GCCAAAGCTT CAGCAATTCA
Htorq    TGGTCTCCTT ATGCTTGTGT CACTCTGATT GCATGGGCTG GTTATGCCAA AGTCTTAACT CCATATTCTA AGTCTGTGCC TGCTGTTATT GCCAAAGCTT CAGCAATTCA
Dindi    TGGTCTCCAT ATGCTTGTGT CACTCTGATT GCATGGGCTG GTTATGCCAA AATCTTAACT CCATATTCTA AGTCTTTGCC TGCTGTTATT GCCAAAGCTT CAGCAATTCA
Smika    TGGTCTCCTT ATGCTTGTGT CACTCTGATT GCGTGGGCTG GTTATGCCAG AATCTTAACT CCATATTCTA AGTCTTTGCC TGCTGTTATT GCCAAAGCTT CAGCAATTCA
Sneuw    TGGTCTCCGT ATGCTTGTGT CACTCTGATT GCATGGGCTG GTTATGCCAG AGTCTTAACT CCATATTCTA AGTCTCTGCC TGCTGTTATT GCCAAAGCTT CAGCAATTCA
Tpers    TGGTCTCCGT ATGCTTGTGT CACTCTGATT GCATGGGCTG GTTATGCCAG AGTCTTAACT CCATATTCTA AGTCTGTGCC TGCTGTTATT GCCAAAGCTT CAGCAATTCA
Eceph    TGGTCTCCAT ATGCTTGTGT CACTCTGATT GCATGGGCTG GTTATGCCAG AGTCTTAACT CCATATTCTA AGTCTGTGCG TGCTGTTATT GCCAAAGCTT CAGCAATTCA
Eundu    TGGTCTCCAT ATGCTTGTGT CACTCTGATT GCATGGGCTG GTTATGCCAA AGTCTTAACT CCATATTCTA AGTCTGTGCC TGCTGTTATT GCCAAAGCTT CAGCAATTCA
Eaesc    TGGTCTCCAT ATGCTTGTGT CACTCTGATT GCATGGGCTG GTTATTCCAG AGTCTTAACA CCATATTCCA AGTCTGTGCC TGCTGTTATT GCCAAAGCTT CAGCAATTCA
Hmode    TGGTCTCCTG ATGCTTGTGT CACTCTGATT GCATGGGCTG GTTATGCCAG AGTCTTAAAT CCATATTCAA AGTCTGTGCC TGCTGTTATT GCCAAAGCTT CAGCAATTCA
Emili    TGGTCTCCTG ATGCTTGCGT CACTCTGATT GCATGGGCTG GTTATTCCAG AGTCTTAACC CCATATTCCA AGTCTGTGCC TGCTGTTATC GCCAAAGCTT CAGCAATTCA
Oguib    TGGTCTCCAT ATGCTTGTGT CACTCTGATT GCGTGGGCTG GTTATGCCAG AGTCTTAAAT CCATATTCTA AGTCTGTGCC TGCTGTTATT GCCAAAGCTT CAGCAATTCA
Ppata    TGGTCTCCAT ATGCTTGTGT CACTCTGATT GCATGGGCTG GTTATGCCAG AGTCTTAACT CCATATTCTA AGTCTGTGCC TGCTGTTATT GCCAAAGCTT CAGCAATTCA
Thypo    TGGTCTCCTG ATGCTTGTGT CACTCTGATT GCATGGGCTG GTTATGCCAG AGTCTTAACT CCATATTCTA AGTCTGTGCC TGCTGTTATT GCCAAAGCTT CAGCAATTCA
Tdors    TGGTCTCCGT ATGCTTGTGT CACTCTGATT GCATGGGCTG GTTATGCCAG AGTCTTAAAT CCATATTCTA AGTCTGTGCC TGCTGTTATC GCCAAAGCTT CAGCAATTCA


         ....|....| ....|....| ....|....| ....|....| ....|....| ....|....| ....|....| ....|....| ....|....| ....|....| ....|....|
                780        790        800        810        820        830        840        850        860        870        880
Pbivi    CAATCCTATA ATTTACGCTA TCATTCACCC AAGTTACAGA AAAACCATTC GAAGAGCTGT TCCTTGTTTG AGATTTCTCA TACGAATATC AACAAGTGAA CTTTCCACAA
Pmucr    CAATCCTATA ATTTATGCTA TTATTCACCC AAGTTACAGG AAAACCATTC GAAAAGCTAT TCCTTGTTTG AGATTTCTCA TACGAATATC TGCAAGTGAA CTTTCCACAA
Bjara    CaAtCcTaTa AtTtAtGcTa TtAtTcAcCc AaGtTaCaGg AaAaCcAtTc GaAaAgCtAt TcCtTgTtTg AgAtTtCtCa TaCgAaTaTc TgCaAgTgAa CtTtCcAcAa
Cduri    CAATCCTATA ATTTATGCTA TTATTCACCC AAGTTACAGG AAAACCATTC GTACAGCTAT TCCTTGTTTG AGATTTCTCA TACGAATATC TGCAAGTGAA CTTTCCACAA
Nscut    CAATCCTATA ATTTATGCTA TTATCCACCC AAGTTACAGG AAAACCATTC GAAGAGCTAT TCCTTGTTTG AGATTTCTCA TACGAATATC CGCAAGCGAA GTTTCCACAA
Ptext    CAATCCTATA ATTTATGCTA TTATCCACCC AAGTTACAGG AAAACGATTC GAAGAGCTAT TCCTTGTTTG AGATTTCTCA TACGAATATC TGCAAGTGAA GTTTCCACAA
Mcora    CAATCCTATA ATTTATGCTA TTATCCACCC AAGTTACAGG AAAACCATTC GAAAAGCTAT TCCTTGTTTG AGATTTCTCA TACGAATATC CGCAAGCGAA GTTTCCACAA
Mlemn    CAATCCTATA ATTTATGCTA TTATCCACCC AAGTTACAGG AAAACCATTC GAAGAGCTAT TCCTTGTTTG AGATTTCTCA TACGAATATC CGCAAGCGAA GTTTCCACAA
Tsirt    CAATCCTATA ATTTATGCTA TTATTCACCC AAGTTACAGG AAAACCATTC GAAGAGCTAT TCCTTGTTTG AGATTTCTCA TACGAATATC CACAAGCGAA CTTTCTACAA
Aeleg    CGGGCCCATA ATTTATGCTA TTATTCACCC AAGTTACAGG AAAACCATTC GAAGAGCTAT TCCTTGTTTG AGATTTCTCA TACGAATATC CGCAAGCGAA CTTTCCACAA
Ccocc    CAATCCCATA ATTTATGCTA TTATTCACCC AAGTTACAGG AAAACCATTC GAAGAGCTAT TCCTTGTTTG AGATTTCTCA TACGAATATC CGCAAGCGAA CTTTCCACAA
Lgetu    CAATCCCATA ATTTATGCTA TTATTCACCC AAGTTACAGG AAAACCATTC GAAGAGCTAT TCCTTGTTTG AGATTTCTCA TACGAATATC CGCAAGCGAA CTTTCCACAA
Pguta    CAATCCCATA ATTTATGCTA TTATTCACCC AAGTTACAGG AAAACCATTC GAAGAGCTAT TCCTTGTTTG AGATTTCTCA TACGAATATC CGCAAGCGAA CTTTCCACAA
Mflag    CAATCCCATA ATTTATGCTA TTATTCACCC AAGTTACAGG AAAACCATTC GAAGAGCTAT TCCTTGTTTG AGATTTCTCA TACGAATATC CGCAAGCGAA CTTTCCACAA
Cbica    CAATCCCATA ATTTATGCTA TTATTCACCC AAGTTACAGG AAAACCATTC GAAGAGCTAT TCCTTGTTTG AGATTTCTCA TACGAATATC TGCAAGCGAA CTTTCCACAA
Htorq    CAATCCTATA ATTTATGCTA TTATTCACCC AAGTTACAGG AAAACCATTC GAAGAGCTAT TCCTTGTTTG AGATTCCTCA TACGATTATC CACAAGCGAA CTTTCCACAA
Dindi    CAATCCTATA ATTTATGCTA TTATTCACCC AAGTTACAGG AAAACCATTC AAAAAGCTAT TCCTTGTTTG AGATTTCTCA TACGAGTATC CACAAGCGAA ATTTCCACAA
Smika    CAATCCTATA ATTTATGCTA TTATTCACCC AAGTTACAGG AAAACCATTC GAAAAGCTAT TCCTTGTTTG AGATTTCTCA TACGAGTATC CACAAGCGAA ATTTCCACAA
Sneuw    CAATCCTATA ATTTATGCTA TTATTCACCC AAGTTACAGG AAAACCATTC GAAAAGCTAT TCCTTGTTTG AGATTTCTCA TACGAGTATC CACAAGCGAA ATTTCCACAA
Tpers    CAATCCTATA ATTTATGCTA TTATTCACCC AAGTTACAGG AAAACCATTC ATAGATCTAT TCCTTGTCTG AGATTTCTCA TACGAATATC CTCAAGCGAA CTTTCCACAA
Eceph    CAATCCTATA ATTTATGCTA TTATTCACCC AAGTTACAGG AAAACCATTC ATAGATCTAT TCCTTGTTTG AGATTTCTCA TACGAATATC CACAAGCGAA CTTACCACAA
Eundu    CAATCCTATA ATTTATGCTA TTATTCACCC AAGTTACAGG AAAACCATTC ATAGATCTAT TCCTTGTTTG AGATTTCTCA TACGAATATC CACAAGCGAA CTTACCACAA
Eaesc    CAATCCTATA ATTTATGCTA TTATTCACCC AAGTTACAGG AAAACCATCG CAAGAGCTAT TCCTTGTTTG AGATTTCTCA TACGGATATC TGCAAGCGAA CTTTCCACAA
Hmode    CAATCCTATA ATTTATGCTA TTATTCACCC AAGTTACAGG AAAACCATTC ATAGAACTAT TCCTTGTTTG AGATTTCTCA TACGAATATC CACAAGCGAA CTTTCCACAA
Emili    CAACCCTATC ATTTATGCTA TTATTCACCC AAGTTACAGG AGAACCATTG CAAGAGCTAT TCCTTGTTTG AGATTTCTCA TACGGATATC TCCAAGCGAA CTTTCCACAA
Oguib    CAATCCTATA ATTTATGCTA TTATTCACCC AAGTTACAGG AAAACCATTC ATAAAGCTAT TCCTTGTTTG AGATTTCTCA TACGAATATC TGCAAGTGAA CTTTCCACAA
Ppata    CAATCCTATA ATTTATGCTA TTATTCACCC AAGTTACAGG AAAACCATTC GAAGAGCTAT TCCTTGTTTG AGATTTCTCA TACGAATATC CACAAGCGAA CTTTCCACAA
Thypo    CAATCCTATA ATTTATGCTA TTATTCACCC AAGTTACAGG AGAACCATTC ATAGAGCTAT TCCTTGTTTG AGATTTCTCA TACGAATATC CACAAGCGAA CTTTCCACAA
Tdors    CAATCCTATA ATTTATGCTA TTATTCACCC AAGTTACAGR AAAACCATTC ACAGAGCTAT TCCTTGTTTG AGATTTCTCA TACGAATATC CACAAGCGAA CTTTCCACAA


         ....|....| ....|....| ....|....| ....|....| ....|....| ....|....| ....|....| ....|....| ....|....| ....|....| ....|....|
                890        900        910        920        930        940        950        960        970        980        990
Pbivi    GTACTGCAAA TGAGTCTTCA TTCAAGACGT CT---ATATC TAGACGTATC TCTTTCGTTT CCAGAAGCAA AAGCAGTGGC ATTTCTTCCA TTTCTGCTGC AGGAAAAACT
Pmucr    GTCTTGCAAA T---TCTTCC TTCAGGTCTT CT---ATGTC TAGTCGTGTC TCTTTTATTT CCAAGAACAA AAGCAATGAC ATTTCTTCCA TTTCTGCTAC AGAAAAAACT
Bjara    GtCtTgCaAa T---TcTtCc TtCaGgTcTt CT---aTgTc TaGtCgTgTc TcTtTcAtTt CcAaGaAcAa AaGcAaTgAc AtTtCtTcCa TtTcTgCtAc AgGaAaAaCt
Cduri    CTCTTGCAAA T---TCTTCC TTCAGGTCTT CT---ATGTC TAGTCGTGTC TCTTTCATTT CCAAGAACAA AAGCAATGAC ATTTCTTCCA TTTCTGCTAC AGGAAAAACT
Nscut    GTCTTGCAAC T---TCTTCC TTCCGGTCTT CT---ATGTC TAGTCGTGTC TCTTTCGTAT CCAAGAACAA AAGCAGTGAC ATTTCCGCCA TTTCTGCTAC AGAAAAAACT
Ptext    GTTTTGCAAC T---TCTTCC TTCCGGTCTT CT---ATGTC TAGTCGTGTC TCTTTCGTAT CCAAGAACAA AAGCAGCGAC ATTTCCGCCA TTTCTGCTAC AGAAAAAACT
Mcora    GTCTTGCAAG T---TCTTCT TTCCGGTCTT CT---ATGTC TAGTCGTGTC TCTTTCGTGT CCAAGAACAA AAGCAGTGAT ATTTCTGCCA TTTCTGCTAC AGAAAAAACT
Mlemn    GTCTTGCAAA T---TCTTCC TTCCGGTCTT CT---ATGTC TAGTCGTGTC TCTTTCGTGT CCAAGAACAA AAGCAGTGAT ATTTCTGCCA TTTCTGCTAC AGAAAAAACT
Tsirt    GTCTTGCAAA T---TCTTCC TTCCGGTCTT CT---GTGTC TAGCCGTGTC TCTTTCATCT CCAAGAACCA AAACTGTGGC ATTTCTTCCA TTTCCGCTAC AGAAAAAACT
Aeleg    GTTTTGCAAA T---TCTTCC TTCCGGTCTT CT---TCTGT GAGTCGTGTC TCTTTTGTGT CCAAGAACAA AAGCAATGAC ATTTCTTGCA TTTCTGCTAC GGAAAAAACT
Ccocc    GTCTTGCAAA T---TCTTCC TTCCGGTCGT CTTCTATGTC TAGTCGTGTC TCTTTCGTGT CCAAGAACAA AAGCAAT--- ATTTCTTCCA TTTCTGCTAC GGAAAAAACT
Lgetu    GTTTTGCAAA T---TCTTCC TTCCGGTCTT CT---TCGTC TAATCGTGTC TCTTTCGTGT CCAAGAACAA AAGCAATGAC ATTTCTTCCA TTTCTGCTAC GGAAAAAACT
Pguta    GTCTTGCAAA T---TCTTCC TTCCGGTCGT CTTCTATGTC TAATCGTGTC TCTTTCGTGT CCAAGAACAA AAGCAATGAC ATTTCTTCCA TTTCTGCTAC GGAA---ACT
Mflag    GTCTTGCAAA T---TCTTCC TTCCGGTCTT CTTCTATGTC TAATCGTGTC TCTTTCGTGT CCAAGAACAA AAGCAATGAC ATTTCTTCCA TTTCTGCTAC GGAAAAGACT
Cbica    GTCTTGCAAA T---TCTTCC TTCCGGTCTT CTTCTATGTC TAGCCGTGTC TCTTTYGTGT CCAAGAACAA AAGCAATGAC ATTTCTTCCA TTTCTGCTAC GGAAAAAACT
Htorq    GTCTTGCAAA T---TCTTCC TTCCGGTCTT CT---ATGTC TAGCCGCGTC TCTTTTGTGT CCAAGAACAG AAACTGTGCC ATTTCTTCCA TTTCTGCTAC AGAAAAAACT
Dindi    GTCTTGCACA T---TCTTCC TTCCGGTCTT CT---ATGTC TAGCCGTGTC TCTTTCGAGT CCAAGAACAG AAGCAGTGCC ATTTCTTCCA TTTCTGCTGC AGAAAAAACT
Smika    GTCTTGCAAA T---TCTTCC TTCCGGTCTT CT---ATGTC TAGCCGTRTC TCTTTCGTGT CCAAGAACRG AAGCAGTGCC ATTTCTTCCA TTTCTGCTGC AGAAAAAACT
Sneuw    GTCTTGCAAA T---TCTTCC TTCCGCTCTT CT---ATGTC TAGCCGTRTC TCTTTCGTGT CCAAGAACAG AAGCAGTGCC ATTTCTTCCA TTTCTGCTGC AGAAAAAACT
Tpers    GCCTTGCAAA T---TCTTCC TTCCGGTCTT CT---ATGTC TAGTCGTGTC TCTTTCGTGT CCAAGAACAA AAACAGTGGC ATTTCTTCCA TTTCTGCTAC AGAAAAAACT
Eceph    GTCTTGCAAG T---TCTTCC TTCCGGTCTT CT---ATGTC TAGTCGTGTC TCTTTCGTGT CCAAGAACAA AAACAGCGGC ATTTCTTCCA TTTCTGCTAC AGAAAAAACT
Eundu    GTCTTGCAAG T---TCTTCC TTCCGGTCTT CT---ATGTC TAGTCGTGTC TCTTTCGTGT CCAAGAACAA AAACAGCGGC ATTTCTTCCA TTTCTGCTAC AGAAAAAACT
Eaesc    GTCTTGCAAG T---TCTTCC TTCCGCTCTT CT-------- -ACTCGTGCC TCTTTCGTGT CCAAGAACAA AAGCAGTGAC ATTTCTTCCA TTTCTGCTAC AGAAAAAAGT
Hmode    GTCTTGCAAA T---TCTTCC TTCCGGTCTT CT---ATGTC TAGTCGTGTC TCTTTTGTGT CGAAGAACAA AACCAGTGGC ATTTCTTCCA TTTCTGCTAC AGAAAAAACT
Emili    GTCTTGCAAG T---TCTTCC TTCCGCTCTT CT-------- -ACTCGTGCG TCTTTCGTCT CCAAGAACAA AAGCAGTGAC ATTTCTTCCA TTTCTGCTGC AGAAAAAACT
Oguib    GTCTTGCAAA T---TCTTCC TTCCGGTCTT CT---ATGTC TAGTCGTGTC TCTTTCGTGT CCAAGAACAA AAGTAGTGGC ATTTGTTCCA TTTCTGCTAC AGAAAAAACT
Ppata    GTTTTGCAAG T---TCTTCC TTCCGGTCTT CT---ATGTC TAGTCGTGTC TCTTTCGTGT CCAAGAACAA AAGCAGTGCC ATTGCTTCCA TTTCTGCTAC AGAAAAAACT
Thypo    GTCTTGCAAA T---TCTTCC TTCCGGTCTT CT---GTGTC TAGTCGTGTC TCTTTCATGT CCAAGAACAA AACCAGTGGC ATTTCTTCCA TTTCTGCTAC AGAAAAAACT
Tdors    GCCTTGCAAA T---TCTTCC TTCAGGTCTT CT---GTGTC TAGTCGTGTC TCTTTCGTGT CCAAGAACAA AACCAGTGGC ATTTCTTCCA TTTCTGCTAC AGAAAAAACT


         ....|....| ....|....| ....|....| ....|....| ....|....| ....|....| ....|....| ....|....| ....|....| ....|....| ....|....|
               1000       1010       1020       1030       1040       1050       1060       1070       1080       1090       1100
Pbivi    TGGAACAATG TAGAGCTTGA TCCTGTGGAG TCAGTCCATA CAAAACTGCA GCCACCTCAA AGTAACTCTT TTTCAACAAA TGCAGAAGAA AAAAGTGAAC TGCCTATGAA
Pmucr    TGGAATGACG TAGAGCTTGA TCCTGTGGAG ACAGTCCATA TAAAATTGCA ATTACCTGAA AGTAACACTT TTTCAACAAA CACAGAAGGA AAAAGTGAAT TGCCTACAAA
Bjara    TgGaAtGaCg TaGaGcTtGa TcCtGtGgAg AcAgTcCaTa TaAaAtTgCa GtTaCcTgAa AgTaAcTcTt TtTcAaCaAa CaCaGaAgGa aGaAgTgAaT tGcCtAcAaA
Cduri    TGGAATGATG TAGAGCTTGA TCCTGTGGAG GCAGTCCATA TAAAATTGCA GTTACGTGAA AGTAACTCTT TTTCAACAAA CACAGTAGGA AGAAGTGAAT TGCCTACAAA
Nscut    TGGAGTGATG TAGAGCTTGA TCCTGTGGAG ACAGTCCATA CAAAATTGCA GCCACCTGAA AATAACTCTT TTTCAACAAA TGCAGAAGAA AAAAGTGAAT TGCCCATCAA
Ptext    TGGAGTGATG TAGAGCTTGA TCCTGTGGAG ACAGTCCATA CAAAATTGCA GCCACCTGAA AATAACTCGT TTTCAACAAA TGCAGAAGAA AAAAGCGAAC TGCCCATCAA
Mcora    TGGAATGATA TAGAGCTTGA CCCTGTGGAA ACAGTCCATA CAAAATTGCA GCCACTTGAA AATAACTCTT TTTCGACAAA TGCAGAAGAA AAAAGTGAAT TGCCCATCAA
Mlemn    TGGAATGATA TAGAGCTTGA CCCTGTGGAA ACAGTCCATA CAAAATTGCA GCCACCTGAA AATAACTCTT TTTCAACAAA TGCAKAAGAA AAATGTGAAT TGCCCATCAA
Tsirt    TGGAATGATG TAGAGCTTGA TCCTGTGGAG ACAGTCCATG CAAAATTGCA GCCACCTGAA AATAACTCTT TTTCAACAAA TGCAGAAGAA AGAAGTGAAT TGCCCACAAG
Aeleg    TGGAATGATG TAGAGCTTGA TCCTGTGGAG ACAGTCCATG CAAAATTGCA GCTACCTGAA AGTAATTCTT TTTCAACAAA TGCAGAAGAA AAAAGTGAAT TGCCCACAAA
Ccocc    TGGAATGATG TAGAGCTTGA TCCTGTGGAG ACAGTCCATG CAAAATTGCA GCTACCTGAA AGTAATTCTT TTTCAACAAA TGCAGAAGAA AAAAGTGAAT TGCCCACAAA
Lgetu    TGGAATGATG TAGAGCTTGA TCCTGTGGAG ACAGTCCATG CAAAATTGCA GCTACCTGAA AGTAATTCTT TTTCAACAAA TGCAGAAGAA AAAAGTGAAT TGCCCACAAA
Pguta    TGGAATGATG TAGAGCTTGA TCCTGTGGAG ACAGTCCATG CAAAATTGCA GCTACCTGAA AGTAATTCTT TTTCAACAAA TGCAGAAGAA AAAAGTGAAT TGCCCACAAA
Mflag    TGGAATGATG TAGAGCTTGA TCCTGTGGAG ACAGTCCATG CAAAATTGCA GCTACCTGAA AGTAATTCTT TTTCAACAAA TGCAGAAGAA AAAAGTGAAT TGCCCACAAA
Cbica    TGGAATGATG TAGAGCTTGA TCCTGTGGAG ACAGTCCATG CAAAATTGCA GCTMCCTGAA AGTAATTCTT TTTCAACAAA TGCAGAAGAA AAAAGTGAAT TGCCCACAAA
Htorq    TGGAATGATG TAGAGCTTGA TCCTGTGGAG ACAGTCCATA CAAAATTGCA GCAAGTTGAA AGTAATTCTT TTTCAACAAA TGCAGAAGAA AAAAGTGAAT TGGCCACAAA
Dindi    TGGAATGATG TAGAGCTTGA TCCTGTGGAG ACAGTCCATA CAAAATTGCA GCAAGCTGAA AGTAATTCTT TTTCAACAAA TGCAGAAGAA AAAAGTGAAT TGGCCACAAA
Smika    TGGAATGATG TAGAGCTTGA TCCTGKGGAG ACAGTCCATA CAAAATTGCA GCAAGCTGAA AGTAATTCTT TTTCAACAAA TGCAGAAGAA AAAAGTGAAT TGGCCGCAAA
Sneuw    TGGAATGATG TAGAGCTTGA TCCTGTGGAG ACAGTCCATA CAAAATTGCA GCAAGCTGAA AGTCATTCTT TTTCAACAAA TGCAGAAGAA AAAAGTGAAT TGGCCACAAA
```

```
Tpers     TGGAATGATG TAGAGCTTGA TCCTGTGGAG ACAGTCCATR CAAAATTGCA GCCTCCTGAA AGTAATTCTT TTTCAACAAA TGCAGAAGAA AAAAGTGAAT TGCCCACAAA
Eceph     TGGAATGATG TAGAGCTTGA TCCTGTGGAG ACAGTCCATA CAAAATTGCA GCCACCTGAA AGTAATTCTT TTTCAACAAA TGCAGAAGAA AAAAGTGAAT TGCCCACAAA
Eundu     TGGAATGATG TAGAGCTTGA TCCTGTGGAG ACAGTCCATA CAAAATTGCA GCCACCTGAA AGTAATTCTT TTTCAACAAA TGCAGAAGAA AAAAGTGAAT TGCCCACAAA
Eaesc     TGGAATGATG TAGAGCTTGA TCCTGTGGAA ACAGTCCATA CAAAATTGCA GCTTCCTGAA AGTATTTCTT TTTCAACAAA TGCAGAAGAA AAAAGTGAAT TGCCCACAAA
Hmode     TGGAATGATG TAGAGCTTGA TCCTGTGGAA ACAGTCCATA CAAAATTGCA GCCACCTGAA AGTAATTCTT TTTCAACAAA TGCAGAAGAA AAAAGTGAAT TACCCACAAA
Emili     TGGAATGATG TAGAGCTTGA TCCTGTGGAA ACAGTCCATA CAAAATTGCA GCTTCCTGAA AGTAATTCTT TTTCAACAAA TGCAGAAGAA AAAAGTGAAT TGCCCACAAA
Oguib     TGGAATGATG TAGAGCTTGA TCCTGTGGAG ACAGTCCATA CAAAATTGCA GCCACCTGAA AGTAATTCTT TTTCAACAAA TGCAGAAGAA AAAAGTGAAT TGCCCACAAA
Ppata     TGGAATGATG TAGAGCTTGA TCCTGTGGAG ACAGTCCATA CAAAATTGCA GTCACCTGAA AGTAATTCTT TTTCAACAAA TGCAGAAGAA AAAAGTGAAT TGCCCACAAA
Thypo     TGGAATGATG TAGAGCTTGA TCCTGTGGAG ACAGTCCATA CAAAATTGCA GCCACCTGAA AGTAATTCTT TTTCAACAAA TGCAGAAGAA AAAAGTGAAT TGCCCACAAA
Tdors     TGGAATGATG TAGAGCTTGA TCCTGTGGAG ACAGTCCATA CAAAATTGCA GCCACCTGAA AGTAATTCTT TTTCAACAAA TGCAGAAGAA AAAAGTGAAT TGCCCACAAA

          ....|....| ....|....| ....|....| ....|....| ....|....| ....|....| ....|....| ....|....| ....|....| ....|....| ....|....|
                1110       1120       1130       1140       1150       1160       1170       1180       1190       1200       1210
Pbivi     GGCCCCGGGC TGTGATGTAC CAACTGAAGA AAAGTTTCCA CTATCTTCCA TCTGTCTAGA AGAACCACCT GAGAGAGCCC TTAAACAAAA GGCTCCAGAA CTGCTCCTCT
Pmucr     TATCCTGAGC TGCAATGCAC CAATTGAAGA AAAGCTTTCA GTATCT---- -----CCAGA AGAACCCCTC AAGAGAGCCT TTAAATTAAA TGCTCCAGAA CTGCTACTCT
Bjara     tAtTcTgAgC tGcAaTgCaT cAaTtGaAgA aAaGcTtTcA gTaTcT---- -----CcAgA aGaAcCcCtC aAgAgAgCcT tTaAaTtAaA tGcTcCaGaA cTgCtAcTcT
Cduri     TATCCCGAGC TGCAATGCAC CAATTGAAGA AAAGCTTTCA GTATCT---- -----CCAGA AGAACCCCTC AAGAGAGCCT TTAAATTAAA TGCTCCAGAG CTGCTACTCT
Nscut     T--------- ------GCAC CAATTGAAGA AAAGTTTTCA GTATCTCCCA TCTGTCCAGA AGAACCACTC AAAAGACGCC TTAAACTAAA TGCTCCAGAA CTGCTTCTCT
Ptext     T--------- ------GCAC CAATTGAAGA AAAGTTTTCA GTATCTCCCA TCTGTCCAGA AGAACCACTC AAAAGATCCC TTAAACTAAA TGCTCCAGAA CTGCTTCTCT
Mcora     T--------- ------GCAC CAATTGAAGA AAAGTTTTCA GTATCTCCCA TCTGTCCAGA AGAACCACTC AAAAGACCCC TTAAACTAAA TGCTCCAGAA CTGCTTCTCT
Mlemn     T--------- ------GCAC CAATTGAAGA AAAGTTTTCA GTATCTCCCA TCTGTCCAGA AGAACCACTC AAAAGACCCC TTAAACTAAA TGCTCCAGAA CTGCTTCTCT
Tsirt     TGCCCTGACC GGTGATGCAC CAATTGAAGA AAAGTTTTCA GTATCTTCCA TCTCTCCAGA AGAACAACTT GAAAGACCCC TTAAATTAAA TGCTCCAGAA CTGCTCCTCT
Aeleg     TGCCCTGAGC TGTGATGCAC CAATTGAAGA AAAGTTTGCA GCATCTCCCA TCTGTCCAGA AGAACCACTC GAAAGACCCC TTAAACTAAA TGCACCAGAA CTGCTACTCT
Ccocc     TGCCCTGAGC TGTGATGCAC CAATTGAAGA AAAGTTTGCA GTATCTCCCA TCTGTCCAGA AGAACCACTC AAAAGACCCC TTAAATTAAA TGCACCAGAA CTGCTACTCT
Lgetu     TGCCCTGAGC TGTGATGCAC CAATTGAAGA AAAGTTTGCA GTATCTCCCA TCTGTCCAGA AGAACCACTC AAAAGACCCC TTAAATTAAA TGCACCAGAA CTGCTACTCT
Pguta     TGCCCTGAGC TGTGATGCAC CAATTGAAGA AAAGTTTGCA GTATCTCCCA TCTGTCCAGA AGAACCACTC AAAAGACCCC TTAAACTAAA TGCACCAGAA CTGCTACTCT
Mflag     TGCCCTGAGC TGTGATGCAC CAATTGAAGA AAAGTTTGCA GTATCTCCCA TCTGTCCAGA AGAACCACTC AAAAGACCCC TTAAACTAAA TGCACCAGAA CTGCTACTCT
Cbica     TGCCCTGAGC TGTGATGCAC CAATTGAAGA AAAGTTTGCA GTATCTTCCA TCTGTCCAGA AGAACCACTY GAAAGACCCC TTAAACTAAA TGCTCCAGAA CTGCTACTCT
Htorq     TGCCCTGAGC TGTGATGCAC CAACTGAAGA AAAGTCTTCA GTATCTCCCG TCTGTCCAGA AGAACCGCTC GAAAGACCCC TTAAACTAAA TGCTCCAGAA CTGCTACTCT
Dindi     TGCCCTGAGC TGTGATGCAC CAACTGAAGA AAAGTCTTCA GTATCTCCCG TCTGTCCAGA AGAACCGCTC AAAAGACCTC TTAAACTGAA TGCTCCAGAA CTGCTACTCT
Smika     TGCCCTGAGC TGTGATGCAC CAACTGAAGA AAAGTCTTCA GTATCTCCCG TCTATCCAGA CGAACTTCTC AAAAGACCCC TTAAACTAAA CGCTCCAGAA CTGCTACTCT
Sneuw     YGCCCTGAGC TGTGATGCAC CAACTGAAGA AAAGTCTTCA GTATCTCCCG TCTGTCCAGA AGAACCGCTC AAAAGACCCC TTAAACTAAA CGCTCCAGAA CTGCTACTCT
Tpers     TGCCCTGAGC TGTGATGCAC CAACTGAAGA AAAGTTTTCG GTATCTCCCA TCTATCCAGA AGAACCACTC GAAAGGCCTC TTAAACTAAA TGCTCCGGAA CTGCTACTCT
Eceph     TGCCCTGAGC WGTGATGCAC CAACTGAAGA AAAGTTTTCG GTATCTCCCA TCTGTCCAGA AGAACCACTY GGAAGGCCTC TTAAACTAAA TGCTCCGGAA CTGCTACTCT
Eundu     TGCCCTGAGC WGTGATGCAC CAACTGAAGA AAAGTTTTCG GTATCTCCCA TCTGTCCAGA AGAACCACTY GGAAGGCCTC TTAAACTAAA TGCTCCGGAA CTGCTACTCT
Eaesc     TGGCCTGAGC TGCGATGCAC CAACTGAAGA AAAGTTATTG GTATCTCCCA TCTGTCCAGA AGAACCACCC CATAGGCCTC TTAAACTAAA TGCTCCAGAA CTGCTACTCT
Hmode     TGCCCTGAGC TGTGATGCAC CAACTGAAGA AAAGTTTTCG GTATCTCCCA TCTGTCCAGA AGAACCACTC GAAAGGCCTC TTAAACTAAA TGCTCCAGAA CTGCTACTCT
Emili     TGACTTGAGC TGTGATCCAC CAACTGAAGA AAAGTTATTG GTATCTCCCA TCTGTCCAGA AGAACCACCC CATAGGCCTC TTAAACTCAA TGCTCCAGAA CTGCTGCTCT
Oguib     TGCCCTGAGC TGTGATGCAC CAACTGAAGA AAAGTTTCCG GTATCTCCCA TCTGTCCAGA AGAACCGCTC GAAAGGCCTC TTAAACTAAA TGCTCCAGAA CTGCTACTCT
Ppata     TGTCCTGAGC TGTGATGCAC CAACTGAAGA AAAGTTTTCG GTATCTCCCA TCTGTCCAGA AGAAYCACTC GGAAGGCCTC TTAAACTAAA TGCTCCAGAA CTGCTACTCT
Thypo     TGCCCTGAGC TATGATGCAC CAACTGAAGA AAAGTTTTCG GTATCTCCCA TCTGTCCAGA AGAACCACTC GAAAGGCCTC TTAAACTAAA TGCTCCAGAA CTGCTACTCT
Tdors     TGCCCTGAAC TGTGATGCAC CAACTGAAGA AAAGTTTTCG GTATCTTCCA TCTGTCCAGA ARAACYACTC GAAAGGCCTC TTAAACTAAA TGCTCCAGAA CTGCTACTCT

          ....|....| ....|....| ....|....| ....|....| ....|....| ....|....| ....|....| ....|....| ....|....| ....|....| ....|....|
                1220       1230       1240       1250       1260       1270       1280       1290       1300       1310       1320
Pbivi     TAAGCAGCTC TTTGAGAACA TCGTCTATTC CATTTGACTT GAATACTAGC ---AGCAGAG GGAAGAGAAT TGACACTTCT CAAGCAGAAA TCCATGAAGA CCAAATAAAT
Pmucr     TAAGCAGCTC CTTGAGAGCA ---------- --TTTGACTT GAATGCTAGC ---AGCAGAG AGAAGAGTAT GGACACTTCT CAAGCAGAAA TCCATGAAGA CCAAATAAAT
Bjara     tAaGcAgGtC cTtGaGaGcA ---------- --tTtGaCtT gAaTgCtAgC ---AgTaGaG aGaAgAgTaT gGaCaCtTcT cAaGcAgAaA tCcAtGaAgA cCaAaTaAaT
Cduri     TAAGCAGGTC CTTGAGAGCA ---------- --TTTGACTT GAATGCTAGC ---TGCAGAG AGAAGAGTAT GGACATTTCT CAAACAGAAA TCCATGAAGA CCAAATAAAT
Nscut     TAACCAGCTC ATTGAGAGCA TCATCTCTTC CATTTGACTT GAATGCTAGC ---AGTAGAG GGAAAAGCAT GGACACCTCT CAAGCAGAAG TCCATGAAGA CCCAATA---
Ptext     TAGCCAGCTC ATTGAGAGCA TCATCTCTTC CATTTGACTT GAATGCTAGC ---AGTAGAG AGAAAAGCAT GGACACCTCT CAAGCAGAAG TCCATGAAGA CCCAGTAAAT
Mcora     TAACCAGCTC ATTGAGAGCA TCATCTCTTC CATTTGACTT GAATGCTAGC ---AGTAGAG TGAAAAGCAT GGACCCCACT CAAGCAGAAG TCCATGAAGA CCCAATAAAT
Mlemn     TAACCAGCTC ATTGAGAGCA TCATCTCTTC CATTTGACTT GAATGCTAGC ---AGTAGAG GGAAAAGCAT GGACCCCACT CAAGCAGAAG TCCATGAAGA CCCAATAAAT
Tsirt     TAACCAGCTC CTTGAGGGCA ACATCTCTTC CATTTGACTT GAATGCCAGC ---AGCAGAG GGAAGAGCCT GGACACCTCT CAAGCAGAAA TCCACGAAGG CCAAATCAAT
Aeleg     TAACCAGCTC CTTGAGAGCA TCATCTCTTC CATTTGACTT GAATGCTAGC ---AGCAGAG GGAAGAGCAT GGACACCTCT CATGCAGAAA TCCATGAAGA CCAAATAAAT
Ccocc     TAACCAGCTC CTTGAGAGCA TCATCTCTTC CATTTGACTT GAATGCTAGC ---AGCAGAG GGAAGAGCAT GGACACCTCT CAAGCAGAAA TCCACGAAGA CGAAATAAAT
Lgetu     TAACCAGCTC CTTGAGAGCA TCATCTCTTT CATTTGACTT GAATGCTAGC ---AGCAGAG GGAAGAGCAT GGACACCTCT CAAGCAGAAA TCCACGAAGA CGAAATAAAT
Pguta     TAACCAGCTC CTTGAGAGCA TCATCTCTTC CATTTGACTT GAATGCTAGC ---AGCAGAG GGAAGAGCAT AGACACCTCT CAAGCAGAAA TCCACGAAGA CGAAATAAAT
Mflag     TAACCAGCTC CTTGAGAGCA TCATCTCTTC CATTTGACTT GAATGCTAGC ---AGCAGAG GGAAGAGCAT GGACACCTCT CAAGCAGAAA TCCACGAAGA CGAAATAAAT
Cbica     TAACCAGCTC CTTGAGAGCA TCATCTCTTC CATTTGACTT GAATGCTAGC ---AGCAGAG GGAACAGCGT GGACACCTCT CAAGCAGAAA TCCACGAAGA CCAAATAAAT
Htorq     TAACCAGCTG CTTAAGAGCA TCATCTCTTC CATTTGACTT GAATGCTAGC ---AGCAGAG GGAAGAGTAT GGACACGTCT CAAGCAGAAA TCCATGAAGA CCAAATAAAT
Dindi     TAACCAGTTG CTTAAGAGCA TCCTCTCTTC CATTTGACTT GAATGCTAGC ---AGCAGAG GGAAGAGCAT GGACRCCTCT CAAGCAGAAA TCCACGAAGG CCAAATAAAT
Smika     TAACCAGTTG CTTAAGAGCA TCCTCTCTTC CATTTGACTT GAATGCTAGC ---AGCAGAG GGAAGAGCGT GGACACCTCT CAAGCAGAAA TCCACGAAGG CCGAATAAAT
Sneuw     TAACCAGTTG CTTAAGAGCA TCCTCTCTTC CATTTGACTT GAATGCTAGC ---AGCAGAG GGAAGAGCAT GGACACCTCT CAAGCAGAAA TCCACGAAGG CCAAATAAAT
Tpers     TAACCAGCTG CTTGAGAGCA TCATCTCTTC CATTTGACTT GAATGCTAAT ---GGCAGAG GAAAGAGCAT GGACACCTCT CAAGCAGAAA TCCACGAAGA CCAAATAAAT
Eceph     TAACCAGCTA CTTGAGAGCA TCATCTCTTC CATTTGACTT GAATGCTAGC ---AGCAGAG GGGAAAGCAT GGATACCTCT CAAGCAGAAA TCCACGAAGA CCAAATAAAT
Eundu     TAACCAGCTA CTTGAGAGCA TCATCTCTTC CATTTGACTT GAATGCTAGC ---AGCAGAG GGGAAAGCAT GGATACCTCT CAAGCAGAAA TCCACGAAGA CCAAATAAAT
Eaesc     TAACCAGCTG CTTGAAAGCA TCGTCTCTTC CATTTGACTT GAATGCTAGC AGCAGCAGAG AGAAGGGTAT GGACACCTCT CAATCAGAAA GCCACGAAGA CCAAATAAGT
Hmode     TAAGCAGCTG CTTGAGAGCA ACATCTCTTC CATTTGACTT GAATGCTAGC ---AGCAGAG GGAAGAGCAC AGATACCTCT CAAGCAGAAA TCCACGAAGA CCAAATAAAT
Emili     TAACCAGCTG CTTGAAAGCA TCGTCTCTTC CATTTGACTT GAATGCTACC AGCAGCAGAG AGAAGAGTAT GGACACCTCT CAATCAGAAA GCCACGAAGA CCAAATAAGT
Oguib     TAACCAGCTG CTTGAGAGCA TCATCTCTTC CATTTTACTT GAATGCTGGC ---AGCAGAG GGAAGGGCAC GGACATCTCT CAAGCAGAAA TCCACGAAGA CCAAATAAAT
Ppata     TAACCAGCTG CTTGAGAGCA TCATCTCTYC CATTTGACTT GAATGCTAGC ---AGCAGAG GGAAGAGCAT GGACACCTCT CAAGCAGAAA TCCATGAAGA CCAAATAAAT
Thypo     TAACCAGCTG CTTGAGAGCA ACAACTCTTC CATTTGACTT GAATGCTAGC ---AGCAGAG GGACGAGCAA GGACACCTCT CAAGCAGAAA TCCACGAAGA CCAAATAAAT
Tdors     TAACCAGCTG CTTGAGAGCA ACAACTCTTC CATTTGACTT GAATGCTAGC ---AGCAGAG GGGCGAGCAA GGACACCTCT CAAGCRGAAA TCCACGAAGA YCAAATAAAT

          ....|....| ....|....| ....|....| ....|....| ....|....| ....|....| ....|....| ....|....| ....|....| ....|....| ....|....|
                1330       1340       1350       1360       1370       1380       1390       1400       1410       1420       1430
Pbivi     AGTAGCCAGG GTTCCTTGGG AAGTCCCACT CTTCCACACA TC-------- ---------- ---ATTATCA TTCCTACCTC AGAAACCACT CTGTCTGAAG AACAAGCTGA
Pmucr     AATAGCCAGG ATTCCTTGGA AAGT------ CTTCCGCGCA TC-------- ---------- ---ATTATCA TTCCTACCTC AGAAACCAGT CTGTCTGAAG AGCAATCTTT
Bjara     aAyAgCcAgG aTtCcTtGgA aAgT------ CTtCcGcGcA tC-------- ---------- ---AtTaTcA tTcCtAcCtC aGaA------ ---------- ----------
Cduri     AATAGCCAAG ATTCTTTGGA AAGT------ CTTCCGCGCA TC-------- ---------- ---ATTATCA TTCCTACCTC AGAAACCAGT CTGTCTGAAG AGCAATCTTT
Nscut     AGTAGTCAGG ATTCCTTGGA AACTCCCATT CTCCCATTCT CCCACACTCC CATTCTCCCA CACTCCCATT CTCTTACCTC AGAAACCACT CTGTCTGAGG AGCAATCTTT
Ptext     ---AGCCAGG ATTCCTTGGA AACTCCCATT CTCCCGCAC- ---------- ---------- ---TCCCATT CTCTTACCTC AGAAACTACT CTGTCTGAAG AGCAATCTTT
Mcora     AGTAGCCAGG ATTCCTTGGA AACTCCCATT CCCCCACACA TC-------- ---------- ---ATCATCA TTCCTACCTC AGAAGCCACT CTGTCTGAAG AGCAATCTTT
Mlemn     AGTAGCCAGG ATTCCTTGGG AACTCCCATT CCCCCACACA TC-------- ---------- ---ATCATCA TTCCTACCTC AGAAGCCACT CTGTCTGAAG AGCAATCTTT
Tsirt     AGTAGCCAGG ATTCCTTGGA AACTCCCATT CTTCCACACA TC-------- ---------- ---ATCATCA TTCCTACCTC AGAAACCACT CTGTCTGAAG AGCAATCTTT
Aeleg     AGTAGCTGGG ATTCCTTGGA AACTCCCATT GTTCCACACA TC-------- ---------- ---ATTATCA TTCCTACCTC AGAAACCACT CTGTCTGAAG AGCAATCTTT
Ccocc     AGTAGCCGGG ATTCCTTGGA AACCCCCATT CTTCCACACA TC-------- ---------- ---ATTATCA TTCCTACCTC AGAAACCACT CTGTCTGAAG AGCAATCTTT
Lgetu     AGTAGCCGGG ATTCCTTGGA AACCCCCATT CTTCCACACA TC-------- ---------- ---ATTATCA TTCCTACCTC AGAAACCACT CTGTCTGAAG AGCAATCTTT
Pguta     AGTAGCCGGG ATTCCTTGGA AACCCCCATT CTTCCACACA TC-------- ---------- ---GTTATCA TTCCTACCTC AGAAACCACT CTGTCTGAAG AGCAATCTTT
Mflag     AGTAGTCGGG ATTCCTTGGA AACCCCCATT CTTCCACACA TC-------- ---------- ---GTTATCA TTCCTACCTC AGAAACCACT CTGTCTGAAG AGCAATCTTT
Cbica     AGTAGCCGGG ATTCCTTGGA AACTCCCATT CTTCCACACA TC-------- ---------- ---ATTATCA TTCCTACCTC AGAAACCACT CTGTCTGAAG AGCAATCTTT
Htorq     AGTAGCCAGG ATTCCTTGGA AACTCCCATT CTTCCACACA TC-------- ---------- ---GTTATCA TTCCTACCTC AGAAACCACT CTGTCTGAAG GGCAATCTTT
Dindi     AGTAGCCAGG ATTCCTTGGA AACTCCCATT TTTCCACACA TC-------- ---------- ---GTTATCA TTCCTACCTC AGAAACCACT CTGTCCGAAG AGCAATCTTT
Smika     AGTAGCCAGG ATTCCTTGGA AACTCCCATT TTTCCACACA TC-------- ---------- ---GTTATCA TTCCTACCGC AGAAATCACT CTGTCCGAAG AGCAATCTTT
Sneuw     AGTAGCCAGG ATTCCTTGGA AACTCCCATT TTTCCACACA TC-------- ---------- ---GTTATCA TTCCTACCTC AGAAACCACT CTGTCCGAAG AGCAATCTTT
Tpers     AGTAGCCAGG ATTCCTTGGA AACTCCCATT CTTCCACACA TC-------- ---------- ---GTTATCA TTCCTACCTC AGAATCCACT CTGTCTGAAG AGCAACCTTT
Eceph     AGTAGCCARG ATTCCTTGGA AACTCCCATT CTTCCACACA TC-------- ---------- ---GTYATCA TTCCTACCTC AGAAACCACT CTGTCTGAAG AGCARTCTTT
Eundu     AGTAGCCARG ATTCCTTGGA AACTCCCATT CTTCCACACA TC-------- ---------- ---GTCATCA TTCCTACCTC AGAAACC--- ---------- ----------
Eaesc     AGTAGCCAGG ATTCCTTGGA AATTCCCATT CTTCCACACA TC-------- ---------- ---------A TTCCTATCTC AGAAACCACT CCGTCAGAAG AGCAATCTTT
Hmode     AGTAGCCAGG ATTCCTTGGA AACTCCCATT CTTCCACACA TC-------- ---------- ---GTTATCA TTCCTACCTC AGAAACCACT CTGTCTGAAG A---------
Emili     AGTAGCCAGG ATTCCTTGGA AATTCCCATT CTTCCGCGCA TC-------- ---------- ---------A TTCCTATCTC AGAAACCACT CTGTCAGAAG AGCAATCTTT
Oguib     AGTAGCCAGG ATTCCTTGGA AACTCCCATT CTTCCACACA TC-------- ---------- ---GTTATCA TTCCTACCTC AGAAACCACT CTGTCTGAAG AGCAATCTTT
Ppata     AGTAGCCAGG ATTCCTTGGA AACTCCCATT CTTCCACACA TC-------- ---------- ---GTTATCA TTCCTACCTC AGAAACCACT CTATCTGAAG AGCAATCTTT
```

```
Thypo   AGTAGCCAGG ATTCCTTGGA AACTCCCATT CTTCCACACA TC-------- ---------- ---GTTATCA TTCCTACCTC AGAAACCACG CTGTCTGAAG AGCAATCTTT
Tdors   AGTAGCCAGG ATTCCTTGGA AACTCCCATT CTTCCACACA TC-------- ---------- ---GTTATCA TTCCTACCTC AGAAACCACG CTGTCTGAAG AGCAATCTTT


        ....|....| ....|....| ....|....| ....|....| ....|....| ....|....| .
                1440       1450       1460       1470       1480       1490
Pbivi   GCCAGAAAAT AGAAAGGAAG AAAACACTGA TTTATTCTTT GCTCAAGAGA AGAACCACCT G
Pmucr   GACAGAAAAT ATAGAGGAAG AAAACATTGA TCCGTACTCT GCTCAAGAAA GGAACCACCT G
Bjara   ---------- ---------- ---------- ---------- ---------- ---------- -
Cduri   GACAGAAAAT ATAGAGGAAA GAAAA----- ---------- ---------- ---------- -
Nscut   GACTGAAAAT ATAGAGGAAG AAAACACTGG TCTGTACTCT GCTCCAGACA AGAACTACCT G
Ptext   GACTGAAAAT ATAGAGGAAG AAAACACTGG TCTGTACTCT GCTCCAGACA AAAACTACCT G
Mcora   GACCGGAAAT ATAGAGGAAG AAAACACTGG TCTATACTCT GCTCCAGACA TGAACTACCT G
Mlemn   GACTGAAAAT ATAGAGGAAG AAAACACTGG TCTATACTCT GCTCCAGACA TGAACTACCT G
Tsirt   GGCCGAAAGC ATAGAGGAAG AAAACACTGA TCTGTACTCT GCTCCAGATA AGAACTACCT G
Aeleg   GACCGAAAAT ATAGAAGAAG AAAACGTTAG TCTGTACTCT GCTCCAGACA AGAACTACCT G
Ccocc   GACCGAAAAT ATAGAAGAAG AAAACGTTAG TCTGTACTCT GCTCCAGACA AGAACTACCT G
Lgetu   GACCGAAAAT ATAGAAGAAG AAAACGTTAG TCTGTACTCT GCTCCAGACA AGAACTACCT G
Pguta   GACCGAAAAT ATAGAAGAAG AAAACGTTAG TCTGTACTCT GCTCCAGACA AGAACTACCT G
Mflag   GACCGAAAAT ATAGAAGAAG AAAACGTTAG TCTGTACTCT GCTCCAGACA AGAACTACCT G
Cbica   GACCGAAAAT ATAGAAGAAG AAAACRTTAG TCTGTACTCT TCTCCAGACA AGAACTACCT G
Htorq   GACCGAAAAT ATAGAGGAAG AAAACACTGA TCTGTATTCT GCTCCAGACA AGAACTACCT G
Dindi   GACCGAA--- ---------- ---------- ---------- ---------- ---------- -
Smika   GACCRAAAAT ATAGAGGAAG AAAACACCCA TCTG------ ---------- ---------- -
Sneuw   GACCGAAAAT ATAGAGGAAG AAAACACCCA TCTGTATTCT GCTCCAGACA AGAACTACCT G
Tpers   GACCCAAAAT ATAGAGGAAG AAAACACGGA TCTGTACTCT GCTCCAGACA AGAACTACCT G
Eceph   GACCGAAAAT ATAGAGGAAG AAAACACTGA TCTGTACTCT GCTCCAGACA AGAACTACCT G
Eundu   ---------- ---------- ---------- ---------- ---------- ---------- -
Eaesc   GACCGACAAT ATAGAGGAAG ACAACGCTGG TCTGTATTCT GCTCCAGAGA AGAACTACCT G
Hmode   ------AAAT ATAGAGGAAG AAAACACTGA TCTGTACTCT GCACCGGACA AGAACTACCT G
Emili   GACCGAAAAT ATAGAGGAAG ACAACGCTGG TCTGTATTCT GCTCCAGAGA AGAACTACCT G
Oguib   GACCGAAAAT ATAGAGGAAG AAAACACTGA TCTGTACTCT GCTCCAGACA AGAACTACCT G
Ppata   GACCGAAATT ATAGAGGAAG AAAACACTGA TCTGTACTCT GATCCAGACA AGAACTACCT G
Thypo   GACCGAAAGT ATAGAGGAAG AAAACACTGA TCTGTACTCT GCTCCAGACA AGAACTACCT G
Tdors   GACCGAAAGT ATAGAGGAAG AAAACACTGA TCTGTACTCT TCTCCAGACA AGAACTACCT G
```
